# Supplementary material for: Discovery and application of insertion-deletion (INDEL) polymorphisms for QTL mapping of early life-history traits in Atlantic salmon
Source: BMC Genomics. 2010 Mar 8;11:156. doi: 10.1186/1471-2164-11-156 (PMC2838853; doi:10.1186/1471-2164-11-156)
Supplement: Additional file 2 — Information on developed 76 locus single-run INDEL panel in Atlantic salmon. Information on fluorescence labeling, primer concentrations, PCR pooling and links to alignments, INDEL motifs and GENESCAN (Burge and Karlin 1997) predictions of genes/exons are available in html format. [file 1471-2164-11-156-S2.ZIP › Additionalfile2/table1309.html]

Contig Alignment

```
Base	A B C D E F G H I J K L M N O P Q R S T U V W X Y Z a b c d e f g h i j k l m n o p q r s t u v w x y z A B C D E F G H I J K L M N O P Q R S T U V W X Y Z   
1	. . G . . . . . . G G . G . . . . . . . . . . . . . . . . . . . . . . . . . . . . . . . . . . . . . . . . . . . . . . . . . . . . . . . . . . . . . . . . .    
2	G . G . . . . . . G G . G . . . . . . . . . . . . . . . . . . . . . . . . . . . . . . . . . . . . . . . . . . . . . . . . . . . . . . . . . . . . . . . . .    
3	G . G . . . . . . G G . G . . . . . . . . . . . . . . . . . . . . . . . . . . . . . . . . . . . . . . . . . . . . . . . . . . . . . . . . . . . . . . . . .    
4	G . G . . . . . . G G . G . . . . . . . . . . . . . . . . . . . . . . . . . . . . . . . . . . . . . . . . . . . . . . . . . . . . . . . . . . . . . . . . .    
5	G . G . . . . . . G G . G . . . . . . . . . . . . . . . . . . . . . . . . . . . . . . . . . . . . . . . . . . . . . . . . . . . . . . . . . . . . . . . . .    
6	A . A . . . . . . A A . A . . . . . . . . . . . . . . . . . . . . . . . . . . . . . . . . . . . . . . . . . . . . . . . . . . . . . . . . . . . . . . . . .    
7	G . G G . . . . . G G . G . . . . . . . . . . . . . . . . . . . . . . . . . . . . . . . . . . . . . . . . . . . . . . . . . . . . . . . . . . . . . . . . .    
8	G . G G . . . . . G G . G . . . . . . . . . . . . . . . . . . . . . . . . . . . . . . . . . . . . . . . . . . . . . . . . . . . . . . . . . . . . . . . . .    
9	G . G G . . . . . G G . G . . . . . . . . . . . . . . . . . . . . . . . . . . . . . . . . . . . . . . . . . . . . . . . . . . . . . . . . . . . . . . . . .    
10	A . A A . . . . . A A . A . . . . . . . . . . . . . . . . . . . . . . . . . . . . . . . . . . . . . . . . . . . . . . . . . . . . . . . . . . . . . . . . .    
11	C . C C . . . . . C C . C . . . . . . . . . . . . . . . . . . . . . . . . . . . . . . . . . . . . . . . . . . . . . . . . . . . . . . . . . . . . . . . . .    
12	C . C C . . . . . C C . C . . . . . . . . . . . . . . . . . . . . . . . . . . . . . . . . . . . . . . . . . . . . . . . . . . . . . . . . . . . . . . . . .    
13	A A A A . . . A A A A . A . . . . . . . . . . . . . . . . . . . . . . . . . . . . . . . . . . . . . . . . . . . . . . . . . . . . . . . . . . . . . . . . .    
14	T T T T . . . T T T T . T . . . . . . . . . . . . . . . . . . . . . . . . . . . . . . . . . . . . . . . . . . . . . . . . . . . . . . . . . . . . . . . . .    
15	G G G G . . . G G G G . G . . . . . . . . . . . . . . . . . . . . . . . . . . . . . . . . . . . . . . . . . . . . . . . . . . . . . . . . . . . . . . . . .    
16	T T T T . . . T T T T . T . . . . . . . . . . . . . . . . . . . . . . . . . . . . . . . . . . . . . . . . . . . . . . . . . . . . . . . . . . . . . . . . .    
17	C C C C . . . C C C C . C . . . . . . . . . . . . . . . . . . . . . . . . . . . . . . . . . . . . . . . . . . . . . . . . . . . . . . . . . . . . . . . . .    
18	A A A A A A . A A A A . A . . . . . . . . . . . . . . . . . . . . . . . . . . . . . . . . . . . . . . . . . . . . . . . . . . . . . . . . . . . . . . . . .    
19	T T T T T T . T T T T . T . . . . . . . . . . . . . . . . . . . . . . . . . . . . . . . . . . . . . . . . . . . . . . . . . . . . . . . . . . . . . . . . .    
20	C C C C C C . C C C C . C . . . . . . . . . . . . . . . . . . . . . . . . . . . . . . . . . . . . . . . . . . . . . . . . . . . . . . . . . . . . . . . . .    
21	T T T T T T . T T T T . T . . . . . . . . . . . . . . . . . . . . . . . . . . . . . . . . . . . . . . . . . . . . . . . . . . . . . . . . . . . . . . . . .    
22	G G G G G G . G G G G . G . . G . . . . . . . . . . . . . . . . . . . . . . . . . . . . . . . . . . . . . . . . . . . . . . . . . . . . . . . . . . . . . .    
23	A A A A A A . A A A A . A . . A . . . . . . . . . . . . . . . . . . . . . . . . . . . . . . . . . . . . . . . . . . . . . . . . . . . . . . . . . . . . . .    
24	A A A A A A . A A A A . A . . A . . . . . . . . . . . . . . . . . . . . . . . . . . . . . . . . . . . . . . . . . . . . . . . . . . . . . . . . . . . . . .    
25	G G G G G G . G G G G . G . . G . . . . . . . . . . . . . . . . . . . . . . . . . . . . . . . . . . . . . . . . . . . . . . . . . . . . . . . . . . . . . .    
26	C C C C C C . C C C C . C . . C . . . . . . . . . . . . . . . . . . . . . . . . . . . . . . . . . . . . . . . . . . . . . . . . . . . . . . . . . . . . . .    
27	A A A A A A . A A A A . A . . A . . . . . . . . . . . . . . . . . . . . . . . . . . . . . . . . . . . . . . . . . . . . . . . . . . . . . . . . . . . . . .    
28	G G G G G G . G G G G . G . . G . . . . . . . . . . . . . . . . . . . . . . . . . . . . . . . . . . . . . . . . . . . . . . . . . . . . . . . . . . . . . .    
29	G G G G G G . G G G G . G . . G . . . . . . . . . . . . . . . . . . . . . . . . . . . . . . . . . . . . . . . . . . . . . . . . . . . . . . . . . . . . . .    
30	G G G G G G . G G G G . G . . G . . . . . . . . . . . . . . . . . . . . . . . . . . . . . . . . . . . . . . . . . . . . . . . . . . . . . . . . . . . . . .    
31	C C C C C C . C C C C . C . . C . . . . . . . . . . . . . . . . . . . . . . . . . . . . . . . . . . . . . . . . . . . . . . . . . . . . . . . . . . . . . .    
32	A A A A A A . A A A A . A A . A . . . . . . . . . . . . . . . . . . . . . . . . . . . . . . . . . . . . . . . . . . . . . . . . . . . . . . . . . . . . . .    
33	G G G G G G . G G G G . G G . G . . . . . . . . . . . . . . . . . . . . . . . . . . . . . . . . . . . . . . . . . . . . . . . . . . . . . . . . . . . . . .    
34	G G G G G G . G G G G . G G . G . . . . . . . . . . . . . . . . . . . . . . . . . . . . . . . . . . . . . . . . . . . . . . . . . . . . . . . . . . . . . .    
35	C C C C C C . C C C C . C C . C . . . . . . . . . . . . . . . . . . . . . . . . . . . . . . . . . . . . . . . . . . . . . . . . . . . . . . . . . . . . . .    
36	T T T T T T . T T T T . T T . T . . . . . . . . . . . . . . . . . . . . . . . . . . . . . . . . . . . . . . . . . . . . . . . . . . . . . . . . . . . . . .    
37	A A A A A A . A A A A . A A . A . . . . . . . . . . . . . . . . . . . . . . . . . . . . . . . . . . . . . . . . . . . . . . . . . . . . . . . . . . . . . .    
38	T T T T T T . T T T T . T T . T . . . . . . . . . . . . . . . . . . . . . . . . . . . . . . . . . . . . . . . . . . . . . . . . . . . . . . . . . . . . . .    
39	A A A A A A . A A A A . A A . A . . . . . . . . . . . . . . . . . . . . . . . . . . . . . . . . . . . . . . . . . . . . . . . . . . . . . . . . . . . . . .    
40	A A A A A A . A A A A . A A . A . . . . . . . . . . . . . . . . . . . . . . . . . . . . . . . . . . . . . . . . . . . . . . . . . . . . . . . . . . . . . .    
41	A A A A A A . A A A A . A A . A . . . . . . . . . . . . . . . . . . . . . . . . . . . . . . . . . . . . . . . . . . . . . . . . . . . . . . . . . . . . . .    
42	A A A A A A . A A A A . A A . A . . . . . . . . . . . . . . . . . . . . . . . . . . . . . . . . . . . . . . . . . . . . . . . . . . . . . . . . . . . . . .    
43	C C C C C C . C C C C . C C . C . . . . . . . . . . . . . . . . . . . . . . . . . . . . . . . . . . . . . . . . . . . . . . . . . . . . . . . . . . . . . .    
44	A A A A A A . A A A A . A A . A . . . . . . . . . . . . . . . . . . . . . . . . . . . . . . . . . . . . . . . . . . . . . . . . . . . . . . . . . . . . . .    
45	C C C C C C . C C C C . C C . C . . . . . . . . . . . . . . . . . . . . . . . . . . . . . . . . . . . . . . . . . . . . . . . . . . . . . . . . . . . . . .    
46	T T T T T T . T T T T . T T . T . . . . . . . . . . . . . . . . . . . . . . . . . . . . . . . . . . . . . . . . . . . . . . . . . . . . . . . . . . . . . .    
47	C C C C C C . C C C C . C C . C . . . . . . . . . . . . . . . . . . . . . . . . . . . . . . . . . . . . . . . . . . . . . . . . . . . . . . . . . . . . . .    
48	T T T T T T . T T T T . T T . T . . . . . . . . . . . . . . . . . . . . . . . . . . . . . . . . . . . . . . . . . . . . . . . . . . . . . . . . . . . . . .    
49	C C C C C C . C C C C . C C . C . . . . . . . . . . . . . . . . . . . . . . . . . . . . . . . . . . . . . . . . . . . . . . . . . . . . . . . . . . . . . .    
50	C C C C C C . C C C C . C C . C . . . . . . . . . . . . . . . . . . . . . . . . . . . . . . . . . . . . . . . . . . . . . . . . . . . . . . . . . . . . . .    
51	G G G G G G . G G G G . G G . G . . . . . . . . . . . . . . . . . . . . . . . . . . . . . . . . . . . . . . . . . . . . . . . . . . . . . . . . . . . . . .    
52	A A A A A A . A A A A . A A . A . . . . . . . . . . . . . . . . . . . . . . . . . . . . . . . . . . . . . . . . . . . . . . . . . . . . . . . . . . . . . .    
53	C C C C C C . C C C C . C C . C . . . . . . . . . . . . . . . . . . . . . . . . . . . . . . . . . . . . . . . . . . . . . . . . . . . . . . . . . . . . . .    
54	A A A A A A . A A A A . A A . A . . . . . . . . . . . . . . . . . . . . . . . . . . . . . . . . . . . . . . . . . . . . . . . . . . . . . . . . . . . . . .    
55	C C C C C C . C C C C . C C . C . . . . . . . . . . . . . . . . . . . . . . . . . . . . . . . . . . . . . . . . . . . . . . . . . . . . . . . . . . . . . .    
56	C C C C C C . C C C C . C C . C . . . . . . . . . . . . . . . . . . . . . . . . . . . . . . . . . . . . . . . . . . . . . . . . . . . . . . . . . . . . . .    
57	A A A A A A A A A A A . A A . A . . . . . . . . . . . . . . . . . . . . . . . . . . . . . . . . . . . . . . . . . . . . . . . . . . . . . . . . . . . . . .    
58	G G G G G G G G G G G . G G . G . . . . . . . . . . . . . . . . . . . . . . . . . . . . . . . . . . . . . . . . . . . . . . . . . . . . . . . . . . . . . .    
59	T T T T T T T T T T T . T T . T . . . . . . . . . . . . . . . . . . . . . . . . . . . . . . . . . . . . . . . . . . . . . . . . . . . . . . . . . . . . . .    
60	G G G G G G G G G G G . G G . G . . . . . . . . . . . . . . . . . . . . . . . . . . . . . . . . . . . . . . . . . . . . . . . . . . . . . . . . . . . . . .    
61	A A A A A A A A A A A . A A . A . . . . . . . . . . . . . . . . . . . . . . . . . . . . . . . . . . . . . . . . . . . . . . . . . . . . . . . . . . . . . .    
62	A A A A A A A A A A A . A A . A . . . . . . . . . . . . . . . . . . . . . . . . . . . . . . . . . . . . . . . . . . . . . . . . . . . . . . . . . . . . . .    
63	C C C C C C C C C C C . C C . C . . . . . . . . . . . . . . . . . . . . . . . . . . . . . . . . . . . . . . . . . . . . . . . . . . . . . . . . . . . . . .    
64	G G G G G G G G G G G . G G . G G . . . . . . . . . . . . . . . . . . . . . . . . . . . . . . . . . . . . . . . . . . . . . . . . . . . . . . . . . . . . .    
65	G G G G G G G G G G G . G G . G G . . . . . . . . . . . . . . . . . . . . . . . . . . . . . . . . . . . . . . . . . . . . . . . . . . . . . . . . . . . . .    
66	A A A A A A A A A A A . A A . A A . . . . . . . . . . . . . . . . . . . . . . . . . . . . . . . . . . . . . . . . . . . . . . . . . . . . . . . . . . . . .    
67	A A A A A A A A A A A . A A . A A . . . . . . . . . . . . . . . . . . . . . . . . . . . . . . . . . . . . . . . . . . . . . . . . . . . . . . . . . . . . .    
68	A A A A A A A A A A A . A A . A A . . . . . . . . . . . . . . . . . . . . . . . . . . . . . . . . . . . . . . . . . . . . . . . . . . . . . . . . . . . . .    
69	T T T T T T T T T T T . T T . T T . . . . . . . . . . . . . . . . . . . . . . . . . . . . . . . . . . . . . . . . . . . . . . . . . . . . . . . . . . . . .    
70	C C C C C C C C C C C . C C . C C . . . . . . . . . . . . . . . . . . . . . . . . . . . . . . . . . . . . . . . . . . . . . . . . . . . . . . . . . . . . .    
71	A A A A A A A A A A A . A A . A A . . . . . . . . . . . . . . . . . . . . . . . . . . . . . . . . . . . . . . . . . . . . . . . . . . . . . . . . . . . . .    
72	A A A A A A A A A A A . A A . A A . . . . . . . . . . . . . . . . . . . . . . . . . . . . . . . . . . . . . . . . . . . . . . . . . . . . . . . . . . . . .    
73	T T T T T T T T T T T . T T . T T . . . . . . . . . . . . . . . . . . . . . . . . . . . . . . . . . . . . . . . . . . . . . . . . . . . . . . . . . . . . .    
74	T T T T T T T T T T T . T T . T T . . . . . . . . . . . . . . . . . . . . . . . . . . . . . . . . . . . . . . . . . . . . . . . . . . . . . . . . . . . . .    
75	G G G G G G G G G G G . G G . G G . . . . . . . . . . . . . . . . . . . . . . . . . . . . . . . . . . . . . . . . . . . . . . . . . . . . . . . . . . . . .    
76	C C C C C C C C C C C . C C . C C . . . . . . . . . . . . . . . . . . . . . . . . . . . . . . . . . . . . . . . . . . . . . . . . . . . . . . . . . . . . .    
77	T T T T T T T T T T T . T T . T T . . . . . . . . . . . . . . . . . . . . . . . . . . . . . . . . . . . . . . . . . . . . . . . . . . . . . . . . . . . . .    
78	T T T T T T T T T T T . T T . T T . . . . . . . . . . . . . . . . . . . . . . . . . . . . . . . . . . . . . . . . . . . . . . . . . . . . . . . . . . . . .    
79	T T T T T T T T T T T . T T . T T . . . . . . . . . . . . . . . . . . . . . . . . . . . . . . . . . . . . . . . . . . . . . . . . . . . . . . . . . . . . .    
80	T T T T T T T T T T T . T T . T T . . . . . . . . . . . . . . . . . . . . . . . . . . . . . . . . . . . . . . . . . . . . . . . . . . . . . . . . . . . . .    
81	C C C C C C C C C C C . C C . C C . . . . . . . . . . . . . . . . . . . . . . . . . . . . . . . . . . . . . . . . . . . . . . . . . . . . . . . . . . . . .    
82	A A A A A A A A A A A A A A . A A A . . . . . . . . . . . . . . . . . . . . . . . . . . . . . . . . . . . . . . . . . . . . . . . . . . . . . . . . . . . .    
83	T T T T T T T T T T T T T T . T T T . . . . . . . . . . . . . . . . . . . . . . . . . . . . . . . . . . . . . . . . . . . . . . . . . . . . . . . . . . . .    
84	A A A A A A A A A A A A A A . A A A . . . . . . . . . . . . . . . . . . . . . . . . . . . . . . . . . . . . . . . . . . . . . . . . . . . . . . . . . . . .    
85	G G G G G G G G G G G G G G . G G G . . . . . . . . . . . . . . . . . . . . . . . . . . . . . . . . . . . . . . . . . . . . . . . . . . . . . . . . . . . .    
86	G G G G G G G G G G G G G G . G G G . . . . . . . . . . . . . . . . . . . . . . . . . . . . . . . . . . . . . . . . . . . . . . . . . . . . . . . . . . . .    
87	A A A A A A A A A A A A A A . A A A . . . . . . . . . . . . . . . . . . . . . . . . . . . . . . . . . . . . . . . . . . . . . . . . . . . . . . . . . . . .    
88	G G G G G G G G G G G G G G . G G G . . . . . . . . . . . . . . . . . . . . . . . . . . . . . . . . . . . . . . . . . . . . . . . . . . . . . . . . . . . .    
89	A A A A A A A A A A A A A A . A A A . . . . . . . . . . . . . . . . . . . . . . . . . . . . . . . . . . . . . . . . . . . . . . . . . . . . . . . . . . . .    
90	A A A A A A A A A A A A A A . A A A . . . . . . . . . . . . . . . . . . . . . . . . . . . . . . . . . . . . . . . . . . . . . . . . . . . . . . . . . . . .    
91	A A A A A A A A A A A A A A . A A A . . . . . . . . . . . . . . . . . . . . . . . . . . . . . . . . . . . . . . . . . . . . . . . . . . . . . . . . . . . .    
92	A A A A A A A A A A A A A A . A A A . . . . . . . . . . . . . . . . . . . . . . . . . . . . . . . . . . . . . . . . . . . . . . . . . . . . . . . . . . . .    
93	A A A A A A A A A A A A A A . A A A . . . . . . . . . . . . . . . . . . . . . . . . . . . . . . . . . . . . . . . . . . . . . . . . . . . . . . . . . . . .    
94	G G G G G G G G G G G G G G . G G G . . . . . . . . . . . . . . . . . . . . . . . . . . . . . . . . . . . . . . . . . . . . . . . . . . . . . . . . . . . .    
95	G G G G G G G G G G G G G G . G G G . . . . . . . . . . . . . . . . . . . . . . . . . . . . . . . . . . . . . . . . . . . . . . . . . . . . . . . . . . . .    
96	T T T T T T T T T T T T T T . T T T . . . . . . . . . . . . . . . . . . . . . . . . . . . . . . . . . . . . . . . . . . . . . . . . . . . . . . . . . . . .    
97	C C C C C C C C C C C C C C . C C C . . . . . . . . . . . . . . . . . . . . . . . . . . . . . . . . . . . . . . . . . . . . . . . . . . . . . . . . . . . .    
98	A A A A A A A A A A A A A A . A A A . . . . . . . . . . . . . . . . . . . . . . . . . . . . . . . . . . . . . . . . . . . . . . . . . . . . . . . . . . . .    
99	A A A A A A A A A A A A A A . A A A . . . . . . . . . . . . . . . . . . . . . . . . . . . . . . . . . . . . . . . . . . . . . . . . . . . . . . . . . . . .    
100	C C C C C C C C C C C C C T . C C C . . . . . . . . . . . . . . . . . . . . . . . . . . . . . . . . . . . . . . . . . . . . . . . . . . . . . . . . . . . .    
101	C C C C C C C C C C C C C C . C C C . . . . . . . . . . . . . . . . . . . . . . . . . . . . . . . . . . . . . . . . . . . . . . . . . . . . . . . . . . . .    
102	C C C C C C C C C C C C C C C C C C . . . . . . . . . . . . . . . . . . . . . . . . . . . . . . . . . . . . . . . . . . . . . . . . . . . . . . . . . . . .    
103	A A A A A A A A A A A A A A A A A A . . . . . . . . . . . . . . . . . . . . . . . . . . . . . . . . . . . . . . . . . . . . . . . . . . . . . . . . . . . .    
104	G G G G G G G G G G G G G G G G G G . . . . . . . . . . . . . . . . . . . . . . . . . . . . . . . . . . . . . . . . . . . . . . . . . . . . . . . . . . . .    
105	G G G G G G G G G G G G G G G G G G . . . . . . . . . . . . . . . . . . . . . . . . . . . . . . . . . . . . . . . . . . . . . . . . . . . . . . . . . . . .    
106	A A A A A A A A A A A A A A A A A A . . . . . . . . . . . . . . . . . . . . . . . . . . . . . . . . . . . . . . . . . . . . . . . . . . . . . . . . . . . .    
107	C C C C C C C C C C C C C C C C C C . . . . . . . . . . . . . . . . . . . . . . . . . . . . . . . . . . . . . . . . . . . . . . . . . . . . . . . . . . . .    
108	G G G G G G G G G G G G G G G G G G . . . . . . . . . . . . . . . . . . . . . . . . . . . . . . . . . . . . . . . . . . . . . . . . . . . . . . . . . . . .    
109	C C C C C C C C C C C C C C C C C C . . . . . . . . . . . . . . . . . . . . . . . . . . . . . . . . . . . . . . . . . . . . . . . . . . . . . . . . . . . .    
110	G G G G G G G G G G G G G G G G G G . . . . . . . . . . . . . . . . . . . . . . . . . . . . . . . . . . . . . . . . . . . . . . . . . . . . . . . . . . . .    
111	A A A A A A A A A A A A A A A A A A . . . . . . . . . . . . . . . . . . . . . . . . . . . . . . . . . . . . . . . . . . . . . . . . . . . . . . . . . . . .    
112	C C C C C C C C C C C C C C C C C C . . . . . . . . . . . . . . . . . . . . . . . . . . . . . . . . . . . . . . . . . . . . . . . . . . . . . . . . . . . .    
113	A A A A A A A A A A A A A A A A A A . . . . . . . . . . . . . . . . . . . . . . . . . . . . . . . . . . . . . . . . . . . . . . . . . . . . . . . . . . . .    
114	G G G G G G G G G G G G G G G G G G . . . . . . . . . . . . . . . . . . . . . . . . . . . . . . . . . . . . . . . . . . . . . . . . . . . . . . . . . . . .    
115	A A A A A A A A A A A A A A A A A A . . . . . . . . . . . . . . . . . . . . . . . . . . . . . . . . . . . . . . . . . . . . . . . . . . . . . . . . . . . .    
116	C C C C C C C C C C C C C C C C C C . . . . . . . . . . . . . . . . . . . . . . . . . . . . . . . . . . . . . . . . . . . . . . . . . . . . . . . . . . . .    
117	C C C C C C C C C C C C C C C C C C . . . . . . . . . . . . . . . . . . . . . . . . . . . . . . . . . . . . . . . . . . . . . . . . . . . . . . . . . . . .    
118	C C C C C C C C C C C C C C C C C C . . . . . . . . . . . . . . . . . . . . . . . . . . . . . . . . . . . . . . . . . . . . . . . . . . . . . . . . . . . .    
119	A A A A A A A A A A A A A A A A A A . . . . . . . . . . . . . . . . . . . . . . . . . . . . . . . . . . . . . . . . . . . . . . . . . . . . . . . . . . . .    
120	G G G G G G G G G G G G G G G G G G . . . . . . . . . . . . . . . . . . . . . . . . . . . . . . . . . . . . . . . . . . . . . . . . . . . . . . . . . . . .    
121	A A A A A A A A A A A A A A A A A A . . . . . . . . . . . . . . . . . . . . . . . . . . . . . . . . . . . . . . . . . . . . . . . . . . . . . . . . . . . .    
122	A A A A A A A A A A A A A A A A A A . . . . . . . . . . . . . . . . . . . . . . . . . . . . . . . . . . . . . . . . . . . . . . . . . . . . . . . . . . . .    
123	C C C C C C C C C C C C C C C C C C . . . . . . . . . . . . . . . . . . . . . . . . . . . . . . . . . . . . . . . . . . . . . . . . . . . . . . . . . . . .    
124	A A A A A A A A A A A A A A A A A A . . . . . . . . . . . . . . . . . . . . . . . . . . . . . . . . . . . . . . . . . . . . . . . . . . . . . . . . . . . .    
125	T T T T T T T T T T T T T T T T T T . . . . . . . . . . . . . . . . . . . . . . . . . . . . . . . . . . . . . . . . . . . . . . . . . . . . . . . . . . . .    
126	T T T T T T T T T T T T T T T T T T . . . . . . . . . . . . . . . . . . . . . . . . . . . . . . . . . . . . . . . . . . . . . . . . . . . . . . . . . . . .    
127	A A A A A A A A A A A A A A A A A A . . . . . . . . . . . . . . . . . . . . . . . . . . . . . . . . . . . . . . . . . . . . . . . . . . . . . . . . . . . .    
128	T T T T T T T T T T T T T T T T T T . . . . . . . . . . . . . . . . . . . . . . . . . . . . . . . . . . . . . . . . . . . . . . . . . . . . . . . . . . . .    
129	C C C C C C C C C C C C C C C C C C . . . . . . . . . . . . . . . . . . . . . . . . . . . . . . . . . . . . . . . . . . . . . . . . . . . . . . . . . . . .    
130	A A A A A A A A A A A A A A A A A A . . . . . . . . . . . . . . . . . . . . . . . . . . . . . . . . . . . . . . . . . . . . . . . . . . . . . . . . . . . .    
131	T T T T T T T T T T T T T T T T T T . . . . . . . . . . . . . . . . . . . . . . . . . . . . . . . . . . . . . . . . . . . . . . . . . . . . . . . . . . . .    
132	C C C C C C C C C C C C C C C C C C . . . . . . . . . . . . . . . . . . . . . . . . . . . . . . . . . . . . . . . . . . . . . . . . . . . . . . . . . . . .    
133	T T T T T T T T T T T T T T T T T T . . . . . . . . . . . . . . . . . . . . . . . . . . . . . . . . . . . . . . . . . . . . . . . . . . . . . . . . . . . .    
134	G G G G G G G G G G G G G G G G G G . . . . . . . . . . . . . . . . . . . . . . . . . . . . . . . . . . . . . . . . . . . . . . . . . . . . . . . . . . . .    
135	A A A A A A A A A A A A A A A A A A . . . . . . . . . . . . . . . . . . . . . . . . . . . . . . . . . . . . . . . . . . . . . . . . . . . . . . . . . . . .    
136	G G G G G G G G G G G G G G G G G G . . . . . . . . . . . . . . . . . . . . . . . . . . . . . . . . . . . . . . . . . . . . . . . . . . . . . . . . . . . .    
137	A A A A A A A A A A A A A A A A A A . . . . . . . . . . . . . . . . . . . . . . . . . . . . . . . . . . . . . . . . . . . . . . . . . . . . . . . . . . . .    
138	G G G G G G G G G G G G G G G G G G . . . . . . . . . . . . . . . . . . . . . . . . . . . . . . . . . . . . . . . . . . . . . . . . . . . . . . . . . . . .    
139	C C C C C C C C C C C C C C C C C C . . . . . . . . . . . . . . . . . . . . . . . . . . . . . . . . . . . . . . . . . . . . . . . . . . . . . . . . . . . .    
140	C C C C C C C C C C C C C C C C C C . . . . . . . . . . . . . . . . . . . . . . . . . . . . . . . . . . . . . . . . . . . . . . . . . . . . . . . . . . . .    
141	T T T T T T T T T T T T T T T T T T . . . . . . . . . . . . . . . . . . . . . . . . . . . . . . . . . . . . . . . . . . . . . . . . . . . . . . . . . . . .    
142	C C C C C C C C C C C C C C C C C C . . . . . . . . . . . . . . . . . . . . . . . . . . . . . . . . . . . . . . . . . . . . . . . . . . . . . . . . . . . .    
143	G G G G G G G G G G G G G G G G G G . . . . . . . . . . . . . . . . . . . . . . . . . . . . . . . . . . . . . . . . . . . . . . . . . . . . . . . . . . . .    
144	G G G G G G G G G G G G G G G G G G . . . . . . . . . . . . . . . . . . . . . . . . . . . . . . . . . . . . . . . . . . . . . . . . . . . . . . . . . . . .    
145	C C C C C C C C C C C C C C C C C C . . . . . . . . . . . . . . . . . . . . . . . . . . . . . . . . . . . . . . . . . . . . . . . . . . . . . . . . . . . .    
146	A A A A A A A A A A A A A A A A A A . . . . . . . . . . . . . . . . . . . . . . . . . . . . . . . . . . . . . . . . . . . . . . . . . . . . . . . . . . . .    
147	T T T T T T T T T T T T T T T T T T . . . . . . . . . . . . . . . . . . . . . . . . . . . . . . . . . . . . . . . . . . . . . . . . . . . . . . . . . . . .    
148	G G G G G G G G G G G G G G G G G G . . . . . . . . . . . . . . . . . . . . . . . . . . . . . . . . . . . . . . . . . . . . . . . . . . . . . . . . . . . .    
149	T T T T T T T T T T T T T T T T T T . . . . . . . . . . . . . . . . . . . . . . . . . . . . . . . . . . . . . . . . . . . . . . . . . . . . . . . . . . . .    
150	C C C C C C C C C C C C C C C C C C . . . . . . . . . . . . . . . . . . . . . . . . . . . . . . . . . . . . . . . . . . . . . . . . . . . . . . . . . . . .    
151	A A A A A A A A A A A A A A A A A A . . . . . . . . . . . . . . . . . . . . . . . . . . . . . . . . . . . . . . . . . . . . . . . . . . . . . . . . . . . .    
152	T T T T T T T T T T T T T T T T T T . . . . . . . . . . . . . . . . . . . . . . . . . . . . . . . . . . . . . . . . . . . . . . . . . . . . . . . . . . . .    
153	A A A A A A A A A A A A A A A A A A . . . . . . . . . . . . . . . . . . . . . . . . . . . . . . . . . . . . . . . . . . . . . . . . . . . . . . . . . . . .    
154	T T T T T T T T T T T T T T T T T T . . . . . . . . . . . . . . . . . . . . . . . . . . . . . . . . . . . . . . . . . . . . . . . . . . . . . . . . . . . .    
155	G G G G G G G G G G G G G G G G G G . . . . . . . . . . . . . . . . . . . . . . . . . . . . . . . . . . . . . . . . . . . . . . . . . . . . . . . . . . . .    
156	A A A A A A A A A A A A A A A A A A . . . . . . . . . . . . . . . . . . . . . . . . . . . . . . . . . . . . . . . . . . . . . . . . . . . . . . . . . . . .    
157	A A A A A A A A A A A A A A A A A A . . . . . . . . . . . . . . . . . . . . . . . . . . . . . . . . . . . . . . . . . . . . . . . . . . . . . . . . . . . .    
158	C C C C C C C C C C C C C C C C C C . . . . . . . . . . . . . . . . . . . . . . . . . . . . . . . . . . . . . . . . . . . . . . . . . . . . . . . . . . . .    
159	G G G G G G G G G G G G G G G G G G . . . . . . . . . . . . . . . . . . . . . . . . . . . . . . . . . . . . . . . . . . . . . . . . . . . . . . . . . . . .    
160	C C C C C C C C C C C C C C C C C C . . . . . . . . . . . . . . . . . . . . . . . . . . . . . . . . . . . . . . . . . . . . . . . . . . . . . . . . . . . .    
161	A A A A A A A A A A A A A A A A A A . . . . . . . . . . . . . . . . . . . . . . . . . . . . . . . . . . . . . . . . . . . . . . . . . . . . . . . . . . . .    
162	T T T T T T T T T T T T T T T T T T . . . . . . . . . . . . . . . . . . . . . . . . . . . . . . . . . . . . . . . . . . . . . . . . . . . . . . . . . . . .    
163	T T T T T T T T T T T T T T T T T T . . . . . . . . . . . . . . . . . . . . . . . . . . . . . . . . . . . . . . . . . . . . . . . . . . . . . . . . . . . .    
164	T T T T T T T T T T T T T T T T T T . . . . . . . . . . . . . . . . . . . . . . . . . . . . . . . . . . . . . . . . . . . . . . . . . . . . . . . . . . . .    
165	T T T T T T T T T T T T T T T T T T . . . . . . . . . . . . . . . . . . . . . . . . . . . . . . . . . . . . . . . . . . . . . . . . . . . . . . . . . . . .    
166	G G G G G G G G G G G G G G G G G G . . . . . . . . . . . . . . . . . . . . . . . . . . . . . . . . . . . . . . . . . . . . . . . . . . . . . . . . . . . .    
167	T T T T T T T T T T T T T T T T T T . . . . . . . . . . . . . . . . . . . . . . . . . . . . . . . . . . . . . . . . . . . . . . . . . . . . . . . . . . . .    
168	C C C C C C C C C C C C C C C C C C . . . . . . . . . . . . . . . . . . . . . . . . . . . . . . . . . . . . . . . . . . . . . . . . . . . . . . . . . . . .    
169	C C C C C C C C C C C C C C C C C C . . . . . . . . . . . . . . . . . . . . . . . . . . . . . . . . . . . . . . . . . . . . . . . . . . . . . . . . . . . .    
170	A A A A A A A A A A A A A A A A A A . . . . . . . . . . . . . . . . . . . . . . . . . . . . . . . . . . . . . . . . . . . . . . . . . . . . . . . . . . . .    
171	T T T T T T T T T T T T T T T T T T . . . . . . . . . . . . . . . . . . . . . . . . . . . . . . . . . . . . . . . . . . . . . . . . . . . . . . . . . . . .    
172	C C C C C C C C C C C C C C C C C C . . . . . . . . . . . . . . . . . . . . . . . . . . . . . . . . . . . . . . . . . . . . . . . . . . . . . . . . . . . .    
173	C C C C C C C C C C C C C C C C C C . . . . . . . . . . . . . . . . . . . . . . . . . . . . . . . . . . . . . . . . . . . . . . . . . . . . . . . . . . . .    
174	A A A A A A A A A A A A A A A A A A . . . . . . . . . . . . . . . . . . . . . . . . . . . . . . . . . . . . . . . . . . . . . . . . . . . . . . . . . . . .    
175	A A A A A A A A A A A A A A A A A A . . . . . . . . . . . . . . . . . . . . . . . . . . . . . . . . . . . . . . . . . . . . . . . . . . . . . . . . . . . .    
176	A A A A A A A A A A A A A A A A A A . . . . . . . . . . . . . . . . . . . . . . . . . . . . . . . . . . . . . . . . . . . . . . . . . . . . . . . . . . . .    
177	G G G G G G G G G G G G G G G G G G . . . . . . G . . . . . . . . . . . . . . . . . . . . . . . . . . . . . . . . . . . . . . . . . . . . . . . . . . . . .    
178	T T T T T T T T T T T T T T T T T T . . . . . . T . . . . . . . . . . . . . . . . . . . . . . . . . . . . . . . . . . . . . . . . . . . . . . . . . . . . .    
179	T T T T T T T T T T T T T T T T T T . . . . . . T . . . . . . . . . . . . . . . . . . . . . . . . . . . . . . . . . . . . . . . . . . . . . . . . . . . . .    
180	T T T T T T T T T T T T T T T T T T . . . . . . T . . . . . . . . . . . . . . . . . . . . . . . . . . . . . . . . . . . . . . . . . . . . . . . . . . . . .    
181	G G G G G G G G G G G G G G G G G G . . . . . . G G . . . . . . . . . . . . . . . . . . . . . . . . . . . . . . . . . . . . . . . . . . . . . . . . . . . .    
182	A A A A A A A A A A A A A A A A A A . . . . . . A A . . . . . . . . . . . . . . . . . . . . . . . . . . . . . . . . . . . . . . . . . . . . . . . . . . . .    
183	G G G G G G G G G G G G G G G G G G . . . . . . G G G . . . . . . . . . . . . . . . . . . . . . . . . . . . . . . . . . . . . . . . . . . . . . . . . . . .    
184	T T T T T T T T T T T T T T T T T T . . . . . . T T T . . . . . . . . . . . . . . . . . . . . . . . . . . . . . . . . . . . . . . . . . . . . . . . . . . .    
185	G G G G G G G G G G G G G G G G G G . . . . . . G G G . . . . . . . . . . . . . . . . . . . . . . . . . . . . . . . . . . . . . . . . . . . . . . . . . . .    
186	A A A A A A A A A A A A A A A A A A . . . . . . A A A . . . . . . . . . . . . . . . . . . . . . . . . . . . . . . . . . . . . . . . . . . . . . . . . . . .    
187	G G G G G G G G G G G G G G G G G G . . . . . . G G G . . . . . . . . . . . . . . . . . . . . . . . . . . . . . . . . . . . . . . . . . . . . . . . . . . .    
188	G G G G G G G G G G G G G G G G G G . . . . . . G G G . . . . . . . . . . . . . . . . . . . . . . . . . . . . . . . . . . . . . . . . . . . . . . . . . . .    
189	T T T T T T T T T T T T T T T T T T . . . . . . T T T . . . . . . . . . . . . . . . . . . . . . . . . . . . . . . . . . . . . . . . . . . . . . . . . . . .    
190	T T T T T T T T T T T T T T T T T T . . . . . . T T T . . . . . . . . . . . . . . . . . . . . . . . . . . . . . . . . . . . . . . . . . . . . . . . . . . .    
191	G G G G G G G G G G G G G G G G G G . . . . . . G G G . . . . . . . . . . . . . . . . . . . . . . . . . . . . . . . . . . . . . . . . . . . . . . . . . . .    
192	A A A A A A A A A A A A A A A A A A . . . . . . A A A . . . . . . . . . . . . . . . . . . . . . . . . . . . . . . . . . . . . . . . . . . . . . . . . . . .    
193	G G G G G G G G G G G G G G G G G G . . . . . . G G G . . . . . . . . . . . . . . . . . . . . . . . . . . . . . . . . . . . . . . . . . . . . . . . . . . .    
194	A A A A A A A A A A A A A A A A A A . . . . . . A A A . . . . . . . . . . . . . . . . . . . . . . . . . . . . . . . . . . . . . . . . . . . . . . . . . . .    
195	A A A A A A A A A A A A A A A A A A . . . . . . A A A . . . . . . . . . . . . . . . . . . . . . . . . . . . . . . . . . . . . . . . . . . . . . . . . . . .    
196	C C C C C C C C C C C C C C C C C C . . . . . . C C C . . . . . . . . . . . . . . . . . . . . . . . . . . . . . . . . . . . . . . . . . . . . . . . . . . .    
197	C C C C C C C C C C C C C C C C C C . . . . . . C C C . . . . . . . . . . . . . . . . . . . . . . . . . . . . . . . . . . . . . . . . . . . . . . . . . . .    
198	C C C C C C C C C C C C C C C C C C . . . . . . C C C . . . . . . . . . . . . . . . . . . . . . . . . . . . . . . . . . . . . . . . . . . . . . . . . . . .    
199	A A A A A A A A A A A A A A A A A A . . . . . . A A A . . . . . . . . . . . . . . . . . . . . . . . . . . . . . . . . . . . . . . . . . . . . . . . . . . .    
200	T T T T T T T T T T T T T T T T T T . . . . . . T T T . . . . . . . . . . . . . . . . . . . . . . . . . . . . . . . . . . . . . . . . . . . . . . . . . . .    
201	G G G G G G G G G G G G G G G G G G . . . . . . G G G . . . . . . . . . . . . . . . . . . . . . . . . . . . . . . . . . . . . . . . . . . . . . . . . . . .    
202	G G G G G G G G G G G G G G G G G G . . . . . . G G G . . . . . . . . . . . . . . . . . . . . . . . . . . . . . . . . . . . . . . . . . . . . . . . . . . .    
203	A A A A A A A A A A A A A A A A A A . . . . . . A A A . . . . . . . . . . . . . . . . . . . . . . . . . . . . . . . . . . . . . . . . . . . . . . . . . . .    
204	A A A A A A A A A A A A A A A A A A . . . . . . A A A . . . . . . . . . . . . . . . . . . . . . . . . . . . . . . . . . . . . . . . . . . . . . . . . . . .    
205	A A A A A A A A A A A A A A A A A A . . . . . . A A A . . . . . . . . . . . . . . . . . . . . . . . . . . . . . . . . . . . . . . . . . . . . . . . . . . .    
206	A A A A A A A A A A A A A A A A A A . . . . . . A A A . . . . . . . . . . . . . . . . . . . . . . . . . . . . . . . . . . . . . . . . . . . . . . . . . . .    
207	G G G G G G G G G G G G G G G G G G . . . . . . G G G . . . . . . . . . . . . . . . . . . . . . . . . . . . . . . . . . . . . . . . . . . . . . . . . . . .    
208	C C C C C C C C C C C C C C C C C C . . . . . . C C C . . . . . . . . . . . . . . . . . . . . . . . . . . . . . . . . . . . . . . . . . . . . . . . . . . .    
209	A A A A A A A A A A A A A A A A A A . . . . . . A A A . . . . . . . . . . . . . . . . . . . . . . . . . . . . . . . . . . . . . . . . . . . . . . . . . . .    
210	T T T T T T T T T T T T T T T T T T . . . . . . T T T . . . . . . . . . . . . . . . . . . . . . . . . . . . . . . . . . . . . . . . . . . . . . . . . . . .    
211	A A A A A A A A A A A A A A A A A A . . . . . . A A A . . . . . . . . . . . . . . . . . . . . . . . . . . . . . . . . . . . . . . . . . . . . . . . . . . .    
212	A A A A A A A A A A A A A C A A A A . . . . . . A A A . . . . . . . . . . . . . . . . . . . . . . . . . . . . . . . . . . . . . . . . . . . . . . . . . . .    
213	C C C C C C C C C C C C C C C C C C . . . . . . C C C . . . . . . . . . . . . . . . . . . . . . . . . . . . . . . . . . . . . . . . . . . . . . . . . . . .    
214	T T T T T T T T T T T T T T T T T T . . . . . . T T T T . . . . . . . . . . . . . . . . . . . . . . . . . . . . . . . . . . . . . . . . . . . . . . . . . .    
215	C C C C C C C C C C C C C C C C C C . . . . . . C C C C . . . . . . . . . . . . . . . . . . . . . . . . . . . . . . . . . . . . . . . . . . . . . . . . . .    
216	T T T T T T T T T T T T T T T T T T . . . . . . T T T T . . . . . . . . . . . . . . . . . . . . . . . . . . . . . . . . . . . . . . . . . . . . . . . . . .    
217	G G G G G G G G G G G G G G G G G G . . . . . . G G G G . . . . . . . . . . . . . . . . . . . . . . . . . . . . . . . . . . G . G . . . . . . . . . . . . .    
218	A A A A A A A A A A A A A A A A A A . . . . . . A A A A . . . . . . . . . . . . . . . . . . . . . . . . . . . . . . . . . . A . A . . . . . . . . . . . . .    
219	A A A A A A A A A A A A A A A A A A . . . . . . A A A A . . . . . . . . . . . . . . . . . . . . . . . . . . . . . . . . . . A . A . . . . . . . . . . . . .    
220	C C C C C C C C C C C C C C C C C C . . . . . C C C C C . . . . . . . . . . . . . . . . . . . . . . . . . . . . . . . . . . C . C . . . . . . . . . . . . .    
221	C C C C C C C C C C C C C C C C C C . . . . . C C C C C . . . . . . . . . . . . . . . . . . . . . . . . . . . . . . . . . . C . C . . . . . . . . . . . . .    
222	G G G G G G G G G G G G G G G G G G . . . . . G G G G G . . . . . . . . . . . . . . . . . . . . . . . . . . . . . . . . . . G . G . . . . . . . . . . . . .    
223	T T T T T T T T T T T T T T T T T T . . . . . T T T T T . . . . . . . . . . . . . . . . . . . . . . . . . . . . . . . . . . T . T . . . . . . . . . . . . .    
224	T T T T T T T T T T T T T T T T T T . . . . . T T T T T . . . . . . . . . . . . . . . . . . . . . . . . . . . . . . . . . . T . T . . . . . . . . . . . . .    
225	G G G G G G G G G G G G G G G G G G . . . . . G G G G G . . . . . . . . . . . . . . . . . . . . . . . . . . . . . . . . . . G . G . . . . . . . . . . . . .    
226	C C C C C C C C C C C C C C C C C C . . . . . C C C C C . . . . . . . . . . . . . . . . . . . . . . . . . . . . . . . . . . C . C . . . . . . . . . . . . .    
227	A A A A A A A A A A A A A A A A A A . . . . . A A A A A . . . . . . . . . . . . . . . . . . . . . . . . . . . . . . . . . . A . A . . . . . . . . . . . . .    
228	T T T T T T T T T T T T T T T T T T . . . . . T T T T T . . . . . . . . . . . . . . . . . . . . . . . . . . . . . . . . . . T . T . . . . . . . . . . . . .    
229	T T T T T T T T T T T T T T T T T T . . . . . T T T T T . . . . . . . . . . . . . . . . . . . . . . . . . . . . . . . . . . T . T . . . . . . . . . . . . .    
230	G G G G G G G G G G G G G G G G G G . . . . . G G G G G . . . . . . . . . . . . . . . . . . . . . . . . . . . . . . . . . . G . G . . . . . . . . . . . . .    
231	T T T T T T T T T T T T T T T T T T . . . . . T T T T T . . . . . . . . . . . . . . . . . . . . . . . . . . . . . . . . . . T T T . . . . . . . . . . . . .    
232	G G G G G G G G G G G G G G G G G G . . . . . G G G G G . . . . . . . . . . . . . . . . . . . . . . . . . . . . . . . . . . G G G . . . . . . . . . . . . .    
233	T T T T T T T T T T T T T T T T T T . . . . . T T T T T . . . . . . . . . . . . . . . . . . . . . . . . . . . . . . . . . . T T T . . . . . . . . . . . . .    
234	T T T T T T T T T T T T T T T T T T . . . . . T T T T T . . . . . . . . . . . . . . . . . . . . . . . . . . . . . . . . . . T T T . . . . . . . . . . . . .    
235	A A A A A A A A A A A A A A A A A A . . . . . A A A A A . . . . . . . . . . . . . . . . . . . . . . . . . . . . . . . . . . A A A . . . . . . . . . . . . .    
236	G G G G G G G G G G G G G G G G G G . . . . . G G G G G . . . . . . . . . . . . . . . . . . . . . . . . . . . . . . . . . . G G G . . . . . . . . . . . . .    
237	C C C C C C C C C C C C C C C C C C . . . . . C C C C C . . . . . . . . . . . . . . . . . . . . . . . . . . . . . . . . . . C C C . . . . . . . . . . . . .    
238	A A A A A A A A A A A A A A A A A A . . . . . A A A A A . . . . . . . . . . . . . . . . . . . . . . . . . . . . . . . . . . A A A . . . . . . . . . . . . .    
239	A A A A A A A A A A A A A A A A A A . . . . . A A A A A . . . . . . . . . . . . . . . . . . . . . . . . . . . . . . . . . . A A A . . . . . . . . . . . . .    
240	T T T T T T T T T T T T T T T T T T . . . . . T T T T T . . . . . . . . . . . . . . . . . . . . . . . . . . . . . . . . . . T T T . . . . . . . . . . . . .    
241	C C C C C C C C C C C C C C C C C C . . . . . C C C C C . . . . . . . . . . . . . . . . . . . . . . . . . . . . . . . . . . C C C . . . . . . . . . . . . .    
242	A A A A A A A A A A A A A A A A A A . . . . . A A A A A . . . . . . . . . . . . . . . . . . . . . . . . . . . . . . . . . . A A A . . . . . . . . . . . . .    
243	T T T T T T T T T T T T T T T T T T . . . . . T T T T T . . . . . . . . . . . . . . . . . . . . . . . . . . . . . . . . . . T T T . . . . . . . . . . . . .    
244	C C C C C C C C C C C C C C C C C C . . . . . C C C C C . . . . . . . . . . . . . . . . . . . . . . . . . . . . . . . . . . C C C . . . . . . . . . . . . .    
245	A A A A A A A A A A A A A A A A A A . . . . . A A A A A . . . . . . . . . . . . . . . . . . . . . . . . . . . . . . . . . . A A A . . . . . . . . . . . . .    
246	T T T T T T T T T T T T T T T T T T . . . . . T T T T T . . . . . . . . . . . . . . . . . . . . . . . . . . . . . . . . . . T T T . . . . . . . . . . . . .    
247	T T T T T T T C T T T T T T T T T T . . . . . C T T T T . . . . . . . . . . . . . . . . . . . . . . . . . . . . . . . . . . T T T . . . . . . . . . . . . .    
248	G G G G G G G G G G G G G G G G G G . . . . . G G G G G . . . . . . . . . . . . . . . . . . . . . . . . . . . . . . . . . . G G G . . . . . . . . . . . . .    
249	T T T T T T T T T T T T T T T T T T . . . . . T T T T T . . . . . . . . . . . . . . . . . . . . . . . . . . . . . . . . . . T T T . . . . . . . . . . . . .    
250	G G G G G G G G G G G G G G G G G G . . . . . G G G G G . . . . . . . . . . . . . . . . . . . . . . . . . . . . . . . . . . G G G . . . . . . . . . . . . .    
251	C C C C C C C C C C C C C C C C C C . . . . . C C C C C . . . . . . . . . . . . . . . . . . . . . . . . . . . C . . . . . . C C C . . . . . . . . . . . . .    
252	T T T T T T T T T T T T T T T T T T . . . . . T T T T T . . . . . . . . . . . . . . . . . . . . . T . . . . . T . . . . . . T T T . . . . . . . . . . . . .    
253	T T T T T T T T T T T T T T T T T T . . . . . T T T T T . . . . . . . . . . . . . . . . . . . . . T . . . . . T . . . . . . T T T . . . . . . . . . . . . .    
254	G G G G G G G G G G G G G G G G G G . . . . . G G G G G . . . . . . . . . . . . . . . . . . . . . G . . . . . G . . . . . . G G G . . . . . . . . . . . . .    
255	T T T T T T T T T T T T T T T T T T . . . . . T T T T T . . . . . . . . . . . . . . . . . . . . . T . . . . . T . . . . . . T T T . . . . . . . . . . . . .    
256	G G G G G G G G G G G G G G G G G G . . . . . G G G G G . . . . . . . . . . . . . . . . . . . . . G . . . . . G . . . . . . G G G . . . . . . . . . . . . .    
257	A A A A A A A A A A A A A A A A A A . . . . . A A A A A . . . . . . . . . . . . . . . . . . . . . A . . . . . A A . . . . . A A A . . . . . . . . . . . . .    
258	G G G G G G G G G G G G G G G G G G . . . . . G G G G G . . . . . . . . . . . . . . . . . . . . . G . . . . . G G . . . . . G G G . . . . . . . . . . . . .    
259	C C C C C C C C C C C C C C C C C C . . . . . C C C C C . . . . . . . . . . . . . . . . . . . . . C . . . . . C C . . . . . C C C . . . . . . . . . . . . .    
260	T T T T T T T T T T T T T T T T T T . . . . . T T T T T . . . . . . . . . . . . . . . . . . . . . T . . . . . T T . . . . . T T T . . . . . . . . . . . . .    
261	C C C C C C C C C C C C C C C C C C . . . . . C C C C C . . . . . . . . . . . . . . . . . . . . . C . . . . . C C . . . . . C C C . . . . . . . . . . . . .    
262	A A A A A A A A A A A A A A A A A A . . . . . A A A A A . . . . . . . . . . . . . . . . . . . . . A . . . . . A A . . . . . A A A . . . . . . . . . . . . .    
263	G G G G G G G G G G G G G G G G G G . . . . . G G G G G . . . . . . . . . . . . . . . . . . . . . G . . . . . G G . . . . . G G G . . . . . . . . . . . . .    
264	G G G G G G G G G G G G G G G G G G . . . . . G G G G G . . . . . . . . . . . . . . . . . . . . . G . . . . . G G . . . . . G G G . . . . . . . . . . . . .    
265	C C C C C C C C C C C C C C C C C C . . . . . C C C C C . . . . . . . . . . . . . . . . . . . . . C . . . . . C C . . . . . C C C . . . . . . . . . . . . .    
266	G G G G G G G G G G G G G G G G G G . . . . . G G G G G . . . . . . . . . . . . . . . . . . . . . G . . . . . G G . . . . . G G G . . . . . . . . . . . . .    
267	T T T T T T T T T T T T T T T T T T . . . . . T T T T T . . . . . . . . . . . . . . . . . . . . . T . . . . . T T . . . . . T T T . . . . . . . . . . . . .    
268	G G G G G G G G G G G G G G G G G G . . . . . G G G G G . . . . . . . . . . . . . . . . . . . . . G . . . . . G G . . . . . G G G . . . . . . . . . . . . .    
269	A A A A A A A A A A A A A A A A A A . . . . . A A A A A . . . . . . . . . . . . . . . A . . . . . A . . A . . A A . . . . A A A A . . . . . . . . . . . . .    
270	A G A A A A A A A A A A A A A A A A . . . . . A A A A A . . . . . . . . . . . . . . . A . . . . . A . . A . . A A . . . . A A A A . . . . . . . . . . . . .    
271	T T T T T T T T T T T T T T T T T T . . . . . T T T T T . . . . . . . . . . . . . . . T . . . . . T . . T . . T T . . . . T T T T . . . . . . . . . . . . .    
272	G G G G G G G G G G G G G G G G G G . . . . . G G G G G . . . . . . . . . . . . . . . G . . . . . G . . G . . G G . . . . G G G G . . . . . . . . . . . . .    
273	A A A A A A A A A A A A A A A A A A . . . . . A A A A A . . . . . . . . . . . . . . . A . . . . . A . . A . A A A . . . . A A A A . . . . . . . . . . . . .    
274	A A A A A A A A A A A A A A A A A A . . . . . A A A A A . . . . . . . . . . . . . . . A . . . . . A . . A . A A A . . . . A A A A . . . . . . . . . . . . .    
275	G G G G G G G G G G G G G G G G G G . . . . . G G G G G . . . . . . . . . . . . . . . G . . . . . G . . G . G G G . . . . G G G G . . . . . . . . . . . . .    
276	T T T T T T T T T T T T T T T T T T . . . . . T T T T T . . . . . . . . . . . . . . . T . . . . . T . . T . T T T . . . . T T T T . . . . . . . . . . . . .    
277	A A A A A - A A A A A A A A A A A A . . . . . A A A A A . . . . . . . . . . . . . . . A . . . . . A . . A . A A A . . . . A A A A . . . . . . . . . . . . .    
278	C C C C C C C C C C C C C C C C C C . . . . . C C C C C . . . . . . . . . . . . . . . C . . . . . C . . C . C C C . . . . C C C C . . . . . . . . . . . . .    
279	A A A A A A A A A A A A A A A A A A . . . . . A A A A A . . . . . . . . . . . . . . . A . . . . . A . . A . A A A . . . . A A A A . . . . . . . . . . . . .    
280	T T T T T T T T T T T T T T T T T T . . . . . T T T T T . . . . . . . . . . . . . . . T . . . . . T . . T . T T T . . . . T T T T . . . . . . . . . . . . .    
281	G G G G G G G G G G G G G G G G G G . . . . . G G G G G . . . . . . . . . . . . . . . G . . . . . G . . G . G G G . . . . G G G G . . . . . . . . . . . . .    
282	A A A A A A A A A A A A A A A A A A . . . . . A A A A A . . . . . . . . . . . . . . . A . . . . . A . . A . A A A . . . . A A A A . . . . . . . . . . . . .    
283	T T T T T T T T T T T T T T T T T T . . . . . T T T T T . . . . . . . . . . . . . . . T . . . . . T . . T . T T T . . . . T T T T . . . . . . . . . . . . .    
284	G G G G G G G G G G G G G G G G G G . . . . . G G G G G . . . . . . . . . . . . . . . G . . . . . G . . G . G G G . . . . G G G G . . . . . . . . . . . . .    
285	C C C C C C C C C C C C C C C C C C . . . . . C C C C C . . . . . . . . . . . . . . . C . . . . . C . . C . C C C . . . . C C C C . . . . . . . . . . . . .    
286	T T T T T T T T T T T T T T T T T T . . . . . T T T T T . . . . . . . . . . . . . . . T . . . . . T . . T . T T T . . . . T T T T . . . . . . . . . . . . .    
287	C C C C C C C C C C C C C C C C C C . . . . . C C C C C . . . . . . . . . . . . . . . C . . . . . C . . C . C C C . . . . C C C C . . . . . . . . . . . . .    
288	T T T T T T T T T T T T T T T T T T . . . . . T T T T T . . . . . . . . . . . . . . . T . . . . . T . . T . T T T . . . . T T T T . . . . . . . . . . . . .    
289	G G G G G G G G G G G G G G G G G G . . . . . G G G G G . . . . . . . . . . . . . . . G . . . . . G . . G . G G G . . . . G G G G . . . . . . . . . . . . .    
290	G G G G G G G G G G G G G G G G G G . . . . . G G G G G . . . . . . . . . . . . . . . G . . . . . G . . G . G G G . . . . G G G G . . . . . . . . . . . . .    
291	A A A A A A A A A A A A A A A A A A . . . . . A A A A A . . . . . . . . . . . . . . . A . . . . . A . . A . A A A . . . . A A A A . . . . . . . . . . . . .    
292	T T T T T T T T T T T T T T T T T T . . . . . T T T T T . . . . . . . . . . . . . . . T . . . . . T . . T . T T T . . . . T T T T . . . . . . . . . . . . .    
293	G G G G G G G G G G G G G G G G G G . . . . . G G G G G . . . . . . . . G . . . . . . G . . . . . G . . G . G G G . . . . G G G G . . . . . . . . . . . . .    
294	T T T T T T T T T T T T T T T T T T . . . . . T T T T T . . . . . . . . T . . . . . . T . . . . . T . . T . T T T . . . . T T T T . . . . . . . . . . . . .    
295	A A A A A A A A A A A A A A A A A A . . . . . A A A A A . . . . . . . . A . . . . . . A . . . . . A . . A . A A A . . . . A A A A . . . . . . . . . . . . .    
296	T T T T T T T T T T T T T T T T T T . . . . . T T T T T . . . . . . . . T . . . . . . T . . . . . T . . T . T T T . . . . T T T T . . . . . . . . . . . . .    
297	T T T T T T T T T T T T T T T T T T . . . . . T T T T T . . . . . . . . T . . . . . . T . . . . . T . . T . T T T . . . . T T T T . . . . . . . . . . . . .    
298	T T T T T T T T T T T T T T T T T T . . . . . T T T T T . . . . . . . . T . . . . . . T . . . . . T . . T . T T T . . . . T T T T . . . . . . . . . . . . .    
299	C C C C C C C C C C C C C C C C C C . . . . . C C C C C . . . . . . . . C . . . . . . C . . . . . C . . C . C C C . . . . C C C C . . . . . . . . . . . . .    
300	T T T T T T T T T T T T T T T T T T . . . . . T T T T T . . . . . . . . T . . . . . . T . . . . . T . . T . T T T . . . . T T T T . . . . . . . . . . . . .    
301	A A A A A A A A A A A A A A A A A A . . . . . A A A A A . . . . . . . . A . . . . . . A . . . . . A . . A . A A A . . . . A A A A . . . . . . . . . . . . .    
302	G G G G G G G G G G G G G G G G G G . . . . . G G G G G . . . . . . . . G . . . . . . G . . . . . G . . G . G G G . . . . G G G G . . . . . . . . . . . . .    
303	A A A A A A A A A A A A A A A A A A . . . . . A A A A A . . . . . . . . A . . . . . . A . . . . . A . . A . A A A . . . . A A A A . . . . . . . . . . . . .    
304	G G G G G G G G G G G G G G G G G G . . . . . G G G G G . . . . . . . . G . . . . . . G . . . . . G . . G . G G G . . . . G G G G . . . . . . . . . . . . .    
305	G G G G G G G G G G G G G G G G G G . . . . . G G G G G . . . . . . . . G . . . . . . G . . . . . G . . G . G G G . . . . G G G G . . . . . . . . . . . . .    
306	A A A A A A A A A A A A A A A A A A . . . . . A A A A A . . . . . . . . A . . . . . . A . . . . . A . . A . A A A . . . . A A A A . . . . . . . . . . . . .    
307	G G G G G G G G G G G G G G G G G G . . . . . G G G G G . . . . . . . . G . . . . . . G . . . . . G . . G . G G G . . . . G G G G . . . . . . . . . . . . .    
308	A A A A A A A A A A A A A A A A A A . . . . . A A A A A . . . . . . . . A . . . . . . A . . . . . A . . A . A A A . . . . A A A A . . . . . . . . . . . . .    
309	A A A A A A A A A A A A A A A A A A . . . . . A A A A A . . . . . . . . A . . . . . . A . . . . . A . . A . A A A . . . . A A A A . . . . . . . . . . . . .    
310	T T T T T T T T T T T T T T T T T T . . . . . T T T T T . . . . . . . . T . . . . . . T . . . . . T . . T . T T T . . . . T T T T . . . . . . . . . . . . .    
311	G G G G G G G G G G G G G G G G G G . . . . . G G G G G . . . . . . . . G . . . . . . G . . . . . G . . G . G G G . . . . G G G G . . . . . . . . . . . . .    
312	A A A A A A A A A A A A A A A A A A . . . . . A A A A A . . . . . . . . A . . . . . . A . . . . . A . . A . A A A . . . . A A A A . . . . . . . . . . . . .    
313	T T T T T T T T T T T T T T T T T T . . . . . T T T T T . . . . . . . . T . . . . . . T . . . . . T . . T . T T T . . . . T C T T . . . . . . . . . . . . .    
314	C C T C C C C C C C C C C C C C C C . . . . . C C C C C . . . . . . . . C . . . . . . C . . . . . C . . C . C C C . . . . C C C C . . . . . . . . . . . . .    
315	T T T T T T T T T T T T T T T T T T . . . . . T T T T T . . . . . . . . T . . . . . . T . . . . . T . . T . T T T . . . . T T T T . . . . . . . . . . . . .    
316	T T T T T T T T T T T T T T T T T T . . . . . T T T T T . . . . . . . . T . . . . . . T . . . . . T . . T . T T T . . . . T T T T . . . . . . . . . . . . .    
317	A A A A A A A A A A A A A A A A A A . . . . . A A A A A . . . . . . . . A . . . . . . A . . . . . A . . A . A A A . . . . A A A A . . . . . . . . . . . . .    
318	C C C C C C C C C C C C C C C C C C . . . . . C C C C C . . . . . . . . C . . . . . . C . . . . . C . . C . C C C . . . . C C C C . . . . . . . . . . . . .    
319	T T T T T T T T T T T T T T T T T T . . . . . T T T T T . . . . . . . . T . . . . . . T . . . . . T . . T . T T T . . . . T T T T . . . . . . . . . . . . .    
320	C C C C C C C C C C C C C C C C C C . . . . . C C C C C . . . . . . . . C . . . . . . C . . . . . C . . C . C C C . . . C C C C C . . . . . . . . . . . . .    
321	A A A A A A A A A A A A A A A A A A . . . . . A A A A A . . . . . . . . A . . . . . . A . . . . . A . . A . A A A . . . A A A A A . . . . . . . . . . . . .    
322	G G G G G G G G G G G G G G G G G G . . . . . G G G G G . . . . . . . . G . . . . . . G . . . . . G . . G . G G G . . . G G G G G . . . . . . . . . . . . .    
323	A A A A A A A A A A A A A A A A A A . . . . . A A A A A . . . . . . . . A . . . . . . A . . . . . A . . A . A A A . . . A A A A A . . . . . . . . . . . . .    
324	T T T T T T T T T T T T T T T T T T . . . . . T T T T T . . . . . . . . T . . . . . . T . . . . . T . . T . T T T . . . T T T T T . . . . . . . . . . . . .    
325	G G G G G G G G G G G G G G G G G G . . . . . G G G G G . . . . . . . . G . . . . . . G . . . . . G . . G . G G G . . . G G G G G . . . . . . . . . . . . .    
326	T T T T T T T T T T T T T T T T T T . . . . . T T T T T . . . . . . . . T . . . . . . T . . . . . T . . T . T T T . . . T T T T T . . . . . . . . . . . . .    
327	T T T T T T T T T T T T T T T T T T . . . . . T T T T T . . . . . . . . T . . . . . . T . . . . . T . . T . T T T . . . T T T T T . . . . . . . . . . . . .    
328	A A A A A A A A A A A A A A A A A A . . . . . A A A A A . . . . . . . . A . . . . . . A . . . . . A . . A . A A A . . . A A A A A . . . . . . . . . . . . .    
329	C C C C C C C C C C C C C C C C C C . . . . . C C C C C . . . . . . . . C . . . . . . C . . . . . C . . C . C C C . . . C C C C C . . . . . . . . . . . . .    
330	T T T T T T T T T T T T T T T T T T . . . . . T T T T T . . . . . . . . T . . . . . . T . . . . . T . . T . T T T . . . T T T T T . . . . . . . . . . . . .    
331	T T T T T T T T T T T T T T T T T T . . . . . T T T T T . . . . . . . . T . . . . . . T . . . . . T . . T . T T T . . . T T T T T . . . . . . . . . . . . .    
332	G G G G G G G G G G G G G G G G G G G . . . . G G G G G . . . . . . . . G . . . . . . G . . . . . G . . G . G G G . . . G G G G G . . . . . . . . . . . . .    
333	G G G G G G G G G G G G G G G G G G G . . . . G G G G G . . . . . . . . G . . . . . . G . . . . . G . . G . G G G . . . G G G G G . . . . . . . . . . . . .    
334	A A A A A A A A A A A A A A A A A A A . . . . A A A A A . . . . . . . . A . . . . . . A . . . . . A . . A . A A A . . . A A A A A . . . . . . . . . . . . .    
335	G G G G G G G G G G G G G G G G G G G . . . . G G G G G . . . . . . . . G . . . . . . G . . . . . G . . G . G G G . . . G G G G G . . . . . . . . . . . . .    
336	G G G G G G G G G G G G G G G G G G G . . . . G G G G G . . . . . . . . G . . . . . . G . . . . . G . . G . G G G . . . G G G G G . . . . . . . . . . . . .    
337	C C C C C C C C C C C C C C C C C C C . . . . C C C C C . . . . . . . . C . . . . . . C . . . . . C . . C . C C C . . . C C C C C . . . . . . . . . . . . .    
338	A A A A A A A A A A A A A A A A A A A . . . . A A A A A . . . . . . . . A . . . . . . A . . . . . A . . A . A A A . . . A A A A A . . . . . . . . . . . . .    
339	C C C C C C C C C C C C C C C C T C C . . . . C C C C C . . . . . . . . C . . . . . . C . C . . . C . . C . C C C . . . C C C C C . . . . . . . . . . . . .    
340	C C C C C C C C C C C C C C C C C C C . . . . C C C C C . . . . . . . . C . . . . . . C . C . . . C . . C . C C C . . . C C C C C . . . . . . . . . . . . .    
341	G G G G G G G G G G G G G G G G G G G . . . . G G G G G . . . . . . . . G . . . . . . G . G . . . G . . G . G G G . . . G G G G G . . . . . . . . . . . . .    
342	G G G G G G G G G G G G G G G G G G G . . . . G G G G G . . . . . . . . G . . . . . . G . G . . . G . . G . G G G . . . G G G G G . . . . . . . . . . . . .    
343	T T T T T T T T T T T T T T T T T T T . . . . T T T T T . . . . . . . . T . . . . . . T . T . . . T . . T . T T T . . . T T T T T . . . . . . . . . . . . .    
344	C C C C C C C C C C C C C C C C C C C . . . . C C C C C . . . . . . . . C . . . . . . C . C . . . C . . C . C C C . . . C C C C C . . . . . . . . . . . . .    
345	T T T T T T T T T T T T T T T T T T T . . . . T T T T T . . . . . . . . T . . . . . . T . T . . . T . . T . T T T . . . T T T T T . . . . . . . . . . . . .    
346	A A A A A A A A A A A A A A A A A A A . . . . A A A A A . . . . . . . . A . . . . . . A . A . . . A . . A . A A A . . . A A A A A . . . . . . . . . . . . .    
347	C C C C C C C C C C C C C C C C C C C . . . . C C C C C . . . . . . . . C . . . . . . C . C . . . C . . C . C C C . . . C C C C C . . . . . . . . . . . . .    
348	A A A A A A A A A A A A A A A A A A A . . . . A A A A A . . . . . . . . A . . . . . . A . A . . . A . . A . A A A . . . A A A A A . . . . . . . . . . . . .    
349	A A A A A A A A A A A A A A A A A A A . . . . A A A A A . . . . . . . . A . . . . . . A . A . . . A . . A . A A A . . . A A A A A . . . . . . . . . . . . .    
350	C C C C C C C C C C C C C C C C C C C . . . . C C C C C . . . . . . . . C . . . . . . C . C C . . C . . C C C C C . . . C C C C C . . . . . . . . . . . . .    
351	C C C C C C C C C C C C C C C C C C C . . . . C C C C C . . . . . . . . C . . . . . . C . C C . . C . . C C C C C . . . C C C C T . . . . . . . . . . . . .    
352	A A A A A A A A A A A A A A A A A A A . . . . A A A A A . . . . . . . . A . . . . . . A . A A . . A . . A A A A A . . . A A A A A . . . . . . . . . . . . .    
353	G G G G G G G G G G G G G G G G G G G . . . . G G G G G . . . . . . . . G . . . . . . G . G G . . G . . G G G G G . . . G G G G G . . . . . . . . . . . . .    
354	A A A A A A A A A A A A A A A A A A A . . . . A A A A A . . . . . . . . A . . . . . . A . A A . . A . . A A A A A . . . A A A A A . . . . . . . . . . . . .    
355	C C C C C C C C C C C C C C C C C C C . . . . C C C C C . . . . . . . . C . . . . . . C . C C . . C . . C C C C C . . . C C C C C . . . . . . . . . . . . .    
356	G G G G G G G G G G G G G G G G G G G . . . G G G G G G . . . . . . . . G . . . . . . G . G G . . G . . G G G G G . G . G G G G G . . . . . . . . . . . . .    
357	C C C C C C C C C C C C C C C C C C C . . . C C C C C C . . . . . . . . C . . . . . . C . C C . . C . . C C C C C . C . C C C C C . . . . . . . . . . . . .    
358	C C C C C C C C C C C C C C C C C C C . . . C C C C C C . . . . . . . . C . . . . . . C . C C . . C . . C C C C C . C . C C C C C . . . . . . . . . . . . .    
359	A A A A A A A A A A A A A A A A A A A . . A A A A A A A . . . . . . . . A . . . . . . A . A A . . A . . A A A A A . A . A A A A A . . . . . . . . . . . . .    
360	T T T T T T T T T T T T T T T T T T T . . T T T T T T T . . . . . . . . T . . . . . . T . T T . . T . . T T T T T . T . T T T T T . . . . . . . . . . . . .    
361	A A A A A A A A A A A A A A A A A A A . . A A A A A A A . . . . . . . . A . . . . . . A . A A A . A . . A A A A A . A A A A A A A . . . . . . . . . . . . .    
362	G G G G G G G G G G G G G G G G G G G . . G G G G G G G . . . . . . . . G . . . . . . G . G G G . G . . G G G G G . G G G G G G G . . . . . . . . . . . . .    
363	C C C C C C C C C C C C C C C C C C C . . C C C C C C C . . . . . . . . C . . . . . . C . C C C . C . . C C C C C . C C C C C C C . . . . . . . . . . . . .    
364	T T T T T T T T T T T T T T T T T T T . . T T T T T T T . . . . . . . . T . . . . . . T . T T T . T . . T T T T T . T T T T T T T . . . . . . . . . . . . .    
365	C C C C C C C C C C C C C C C C C C C . . C C C C C C C . . . . . . . . C . . . . . . C . C C C . C . . C C C C C . C C C C C C C . . . . . . . . . . . . .    
366	A A A A A A A A A A A A A A A A A A A . . A A A A A A A . . . . . . . . A . . . . . . A . A A A . A . . A A A A A . A A A A A A A . . . . . . . . . . . . .    
367	G G G G G G G G G G G G G G G G G G G . . G G G G G G G . . . . . . . . G . . . . . . G . G G G . G . . G G G G G . G G G G G G G . . . . . . . . . . . . .    
368	C C C C C C C C C C C C C C C C C C C . . C C C C C C C . . . . . . . . C . . . . . . C . C C C . C . . C C C C C . C C C C C C C . . . . . . . . . . . . .    
369	C C C C C C C C C C C C C C C C C C C . . C C C C C C C . . . . . . . . C . . . . . . C . C C C . C . . C C C C C . C C C C C C C . . . . . . . . . . . . .    
370	T T T T T T T T T T T T T T T T T T T . . T T T T T T T . . . . . . . . T . . . . . . T . T T T . T . . T T T T T . T T T T T T T . . . . . . . . . . . . .    
371	G G G G G G G G G G G G G G G G G G G . . G G G G G G G . . . . . . . . G . . . . . . G . G G G . G . . G G G G G G G G G G G G G . . . . . . . . . . . . .    
372	C C C C C C C C C C C C C C C C C C C . . C C C C C C C . . . . . . . . C . . . . . . C . C C C . C . . C C C C C C C C C C C C C . . . . . . . . . . . . .    
373	A A A A A A A A A A A A A A A A A A A . . A A A A A A A . . . . . . . . A . . . . . . A . A A A . A . . A A A A A A A A A A A A A . . . . . . . . . . . . .    
374	C C C C C C C C C C C C C C C C C C C . . C C C C C C C . . . . . . . . C . . . . . . C . C C C . C . . C C C C C C C C C C C C C . . . . . . . . . . . . .    
375	C C C C C C C C C C C C C C C C C C C . . C C C C C C C . . . . . . . . C . . . . . . C . C C C . C . . C C C C C C C C C C C C C . . . . . . . . . . . . .    
376	C C C C C C C C C C C C C C C C C C C . . C C C C C C C . . . . . . . . C . . . . . . C . C C C . C . . C C C C C C C C C C C C C . . . . . . . . . . . . .    
377	T T T T T T T T T T T T T T T T T T T . . T T T T T T T . . . . . . . . T . . . . . . T . T T T . T . . T T T T T T T T T T T T T . . . . . . . . . . . . .    
378	- - - - - - - - - - - - - - - - - - - . . - - - - - - - . . . . . . . . T . . . . . . - . - - - . - . . - - - - - - - - - - - - - . . . . . . . . . . . . .    
379	C C C C C C C C C C C C C C C C C C C . . C C C C C C C . . . . . . . . C . . . . . . C . C C C . C . . C C C C C C C C C C C C C . . . . . . . . . . . . .    
380	T T T T T T T T T T T T T T T T T T T . . T T T T T T T . . . . . . . . T . . . . . . T . T T T . T . . T T T T T T T T T T T T T . . . . . . . . . . . . .    
381	C C C C C C C C C C C C C C C C C C C . . C C C C C C C . . . . . . . . C . . . . . . C . C C C . C . . C C C C C C C C C C C C C . . . . . . . . . . . . .    
382	T T T T T T T T T T T T T T T T T T T . . T T T T T T T . . . . . . . . T . . . . . . T . T T T . T . . T T T T T T T T T T T T T . . . . . . . . . . . . .    
383	G G G G G G G G G G G G G G G G G G G . . G G G G G G G . . . . . . . G G . . . . . . G . G G G . G . . G G G G G G G G G G G G G . . . . . . . . . . . . .    
384	T T T T T T T T T T T T T T T T T T T . . T T T T T T T . . . . . . . T T . . . . . . T . T T T . T . . T T T T T T T T T T T T T . . . . . . . . . . . . .    
385	G G G G G G G G G G G G G G G G G G G . . G G G G G G G . . . . . . G G G . . . . . . G . G G G . G . . G G G G G G G G G G G G G . . . . . . . . . . . . .    
386	G G G G G G G G G G G G G G G G G G G . . G G G G G G G . . . . . . G G G . . . . . . G . G G G . G . . G G G G G G G G G G G G G . . . . . . . . . . . . .    
387	G G G G G G G G G G G G G G G G G G G . . G G G G G G G . . . . . . G G G . . . . . . G . G G G . G . . G G G G G G G G G G G G G . . . . . . . . . . . . .    
388	A A A A A A A A A A A A A A A A A A A . . A A A A A A A . . . . . . A A A . . . . . . A . A A A . A . . A A A A A A A A A A A A A . . . . . . . . . . . . .    
389	C C C C C C C C C C C C C C C C C C C . . C C C C C C C . . . . . . C C C . . . . . . C . C C C . C . . C C C C C C C C C C C C C . . . . . . . . . . . . .    
390	A A A A A A A A A A A A A A A A A A A . . A A A A A A A . . . . . . A A A . . . . . . A . A A A . A . . A A A A A A A A A A A A A . . . . . . . . . . . . .    
391	G G G G G G G G G G G G G G G G G G G . . G G G G G G G . . . . . . G G G . . . . . . G . G G G . G . . G G G G G G G G G G G G G . . . . . . . . . . . . .    
392	C C C C C C C C C C C C C C C C C C C . . C C C C C C C . . . . . . C C C . . . . . . C . C C C . C . . C C C C C C C C C C C C C . . . . . . . . . . . . .    
393	T T T T T T T T T T T T T T T T T T T . . T T T T T T T . . . . . . T T T . . . . . . T . T T T . T . . T T T T T T T T T T T T T . . . . . . . . . . . . .    
394	T T T T T T T T T T T T T T T T T T T . . T T T T T T T . . . . . . T T T . . . . . . T . T T T . T . . T T T T T T T T T T T T T . . . . . . . . . . . . .    
395	G G G G G G G G G G G G G G G G G G G . . G G G G G G G . . . . . . G G G . . . . . . G . G G G . G . . G G G G G G G G G G G G G . . . . . . . . . . . . .    
396	C C C C C C C C C C C C C C C C C C C . . C C C C C C C . . . . . . C C C . . . . . . C . C C C . C . . C C C C C C C C C C C C C . . . . . . . . . . . . .    
397	T T T T T T T T T T T T T T T T T T T . . T T T T T T T . . . . . . T T T . . . . . . T . T T T T T . . T T T T T T T T T T T T T . . . . . . . . . . . . .    
398	G G G G G G G G G G G G G G G G G G G . . G G G G G G G . . . . . . G G G . . . . . . G . G G G G G . . G G G G G G G G G G G G G . . . . . . . . . . . . .    
399	A A A A A A A A A A A A A A A A A A A . . A A A A A A A . . . . . . A A A . . . . . . A . A A A A A . . A A A A A A A A A A A A A . . . . . . . . . . . . .    
400	G G G G G G G G G G G G G G G G G G G . . G G G G G G G . . . . . . G G G . . . . . . G . G G G G G . . G G G G G G G G G G G G G . . . . . . . . . . . . .    
401	T T T T T T T T T T T T T T T T T T T . . T T T T T C T . . . . . . T T T . . . . . . T . T T T T T . . T T T T T T T T T T T T T . . . . . . . . . . . . .    
402	T T T T T T T T T T T T T T T T T T T . . T T T T T T T . . . . . . T T T . . . . . . T . T T T T T . . T T T T T T T T T T T T T . . . . . . . . . . . . .    
403	G G G G G G G G G G G G G G G G G G G . . G G G G G G G . . . . . . G G G . . . . . . G . G G G G G . . G G G G G G G G G G G G G . . . . . . . . . . . . .    
404	G G G G G G G G G G G G G G G G G G G . . G G G G G G G . . . . . . G G G . . . . . . G . G G G G G . . G G G G G G G G G G G G G . . . . . . . . . . . . .    
405	G G G G G G G G G G G G G G G G G G G . . G G G G G G G . . . . . . G G G . . . . . . G . G G G G G . . G G G G G G G G G G G G G . . . . . . . . . . . . .    
406	G G G G G G G G G G G G G G G G G G G . . G G G G G G G . . . . . . G G G . . . . . . G . G G G G G . . G G G G G G G G G G G G G . . . . . . . . . . . . .    
407	A A A A A A A A A A A A A A A A A A A . . A A A A A A A . . . . . . A A A . . . . . . A . A A A A A . . A A A A A A A A A A A A A . . . . . . . . . . . . .    
408	T T T T T T T T T T T T T T T T T T T . . T T T T T C T . . . . . . T T T . . . . . . T . T T T T T . . T T T T T T T T T T T T T . . . . . . . . . . . . .    
409	C C C C C C C C C C C C C C C C C C C . . C C C C C C C . . . . . C C C C . . . . . . C . C C C C C . . C C C C C C C C C C C C C . . . . . . . . . . . . .    
410	A A A A A A A A A A A A A A A A A A A . . A A A A A A A . . . . . A A A A . . . . . . A . A A A A A . . A A A A A A A A A A A A A . . . . . . . . . . . . .    
411	G G G G G G G G G G G G G G G G G G G . . G G G G G G G . . . . . G G G G . . . . . . G . G G G G G . . G G G G G G G G G G G G G . . . . . . . . . . . . .    
412	A A A A A A A A A A A A A A A A A A A . . C A A A A A A . . . . . A A A A . . . . . . A . A A A A A . . A A A A A A A A A A A A A . . . . . . . . . . . . .    
413	C C C C C C C C C C C C C C C C C C C . . C C C C C C C . . . . . C C C C . . . . . . C . C C C C C . . C C C C C C C C C C C C C . . . . . . . . . . . . .    
414	G G G G G G G G G G G G G G G G G G G . . G G G G G G G . . . . . G G G G . . . . . . G . G G G G G . . G G G G G G G G G G G G G . . . . . . . . . . . . .    
415	A A A A A A A A A A A A A A A A A A A . . A A A A A A A . . . . . A A A A . . . . . . A . A A A A A . . A A A A A A A A A A A A A . . . . . . . . . . . . .    
416	T T T T T T T T T T T T T T T T T T T . . T T T T T T T . . . . . T T T T . . . . . . T . T T T T T . . T T T T T T T T T T T T T . . . . . . . . . . . . .    
417	G G G G G G G G G G G G G G G G G G G . . G G G G G G G . . . . . G G G G . . . . . . G . G G G G G . . G G G G G G G G G G G G G . . . . . . . . . . . . .    
418	G G G G G G G G G G G G G G G G G G G . . G G G G G G G . . . . . G G G G . . . . . . G . G G G G G . . G G G G G G G G G G G G G . . . . . . . . . . . . .    
419	A A A A A A A A A A A A A A A A A A A . . A A A A A A A . . . . A A A A A . . . . . A A . A A A A A . . A A A A A A A A A A A A A . . . . . . . . . . . . .    
420	A A A A A A A A A A A A A A A A A A A . . A A A A A A A . . . . A A A A A . . . . . A A . A A A A A . . A A A A A A A A A A A A A . . . . . . . . . . . . .    
421	G G G G G G G G G G G G G G G G G G G . . G G G G G G G . . . . G G G G G . . . . . G G . G G G G G . . G G G G G G G G G G G G G . . . . . . . . . . . . .    
422	A A A A A A A A A A A A A A A A A A A . . A A A A A A A . . . . A A A A A . . . . . A A . A A A A A . . A A A A A A A A A A A A A . . . . . . . . . . . . .    
423	A A A A A A A A A A A A A A A A A A A . . A A A A A A A . . . . A A A A A . . . . . A A . A A A A A . . A A A A A A A A A A A A A . . . . . . . . . . . . .    
424	A A A A A A A A A A A A A A A A A A A . . A A A A A A A . . . . A A A A A . . . . . A A . A A A A A . . A A A A A A A A A A A A A . . . . . . . . . . . . .    
425	A A A A A A A A A A A A A A A A A A A . . A A A A A A A . . . . A A A A A . . . . . A A . A A A A A . . A A A A A A A A A A A A A . . . . . . . . . . . . .    
426	- - - - - - - - - - - - - - - - - - A . . - - - - - - - . . . . - - - - - . . . . . - - . - - - - - . . - - - - - - - - - - - - - . . . . . . . . . . . . .    
427	G G G G G G G G G G G G G G G G G G G . . G G G G G G G . . . . G G G G G . . . . . G G . G G G G G . . G G G G G G G G G G G G G . . . . . . . . . . . . .    
428	G G G G G G G G G G G G G G G G G G G . . G G G G G G G . . . . G G G G G . . . . . G G . G G G G G G . G G G G G G G G G G G G G . . . . . . . . . . . . .    
429	A A A A A A A A A A A A A A A A A A A . . A A A A A A A . . . . A A A A A . . . . . A A . A A A A A A . A A A A A A A A A A A A A . . . . . . . . . . . . .    
430	A A A A A A A A A A A A A A A A A A A . . A A A A A A A . . . . A A A A A . . . . . A A . A A A A A A . A A A A A A A A A A A A A . . . . . . . . . . . . .    
431	A A A A A A A A A A A A A A A A A A A . . A A A A A A A . . . . A A A A A . . . . . A A . A A A A A A . A A A A A A A A A A A A A . . . . . . . . . . . . .    
432	T T T T T T T T T T T T T T T T T T T . . T T T T T T T . . . . T T T T T . . . . . T T . T T T T T T . T T T T T T T T T T T T T . . . . . . . . . . . . .    
433	C C C C C C C C C C C C C C C C C C C . . C C C C C C C . . . . C C C C C . . . . . C C . C C C C C C . C C C C C C C C C C C C C . . . . . . . . . . . . .    
434	C C C C C C C C C C C C C C C C C C C . . C C C C C C C . . . . C C C C C . . . . . C C . C C C C C C . C C C C C C C C C C C C C . . . . . . . . . . . . .    
435	A A A A A A A A A A A A A A A A A A A . . A A A A A A A . . . . A A A A A . . . . . A A . A A A A A A . A A A A A A A A A A A A A . . . . . . . . . . . . .    
436	A A A A A A A A A A A A A A A A A A A . . A A A A A A A . . . . A A A A A . . . . . A A . A A A A A A . A A A A A A A A A A A A A . . . . . . . . . . . . .    
437	A A A A A A A A A A A A A A A A A A A . . A A A A A A A . . . . A A A A A . . . . . A A . A A A A A A . A A A A A A A A A A A A A . . . . . . . . . . . . .    
438	G G G G G G G G G G G G G G G G G G G . . G G G G G G G . . . . G G G G G . . . . . G G . G G G G G G . G G G G G G G G G G G G G . . . . . . . . . . . . .    
439	A A A A A A A A A A A A A A A A A A A . . A A A A A A A . . . . A A A A A . . . . . A A . A A A A A A . A A A A A A A A A A A A A . . . . . . . . . . . . .    
440	G G G G G G G G G G G G G G G G G G G . . G G G G G G G . . . . G G G G G . . . . . G G . G G G G G G . G G G G G G G G G G G G G . . . . . . . . . . . . .    
441	A A A A A A A A A A A A A A A A A A A . . A A A A A A A . . . . A A A A A . . . . . A A . A A A A A A . A A A A A A A A A A A A A . . . . . . . . . . . . .    
442	A A A A A A A A A A A A A A A A A A A . . A A A A A A A . . . . A A A A A . . . . . A A . A A A A A A . A A A A A A A A A A A A A . . . . . . . . . . . . .    
443	G G G G G G G G G G G G G G G G G G G . . G G G G G G G . . . . G G G G G . . . . . G G . G G G G G G . G G G G G G G G G G G G G . . . . . . . . . . . . .    
444	A A G A A A A A A A A A A A A A A A A . . A A A A A A A . . . . A A A A A . . . . . A A . A A A A A A . A A A A A A A A A A A A A . . . . . . . . . . . . .    
445	G A G G G G G G G G G G G G G G G G G . . G G G G G G G . . . . G G G G G . . . . . G G . G G G A G G . G G G G G G G G G G G G G . . . . . . . . . . . . .    
446	G G G G G G G G G G G G G G G G G G G . . G G G G G G G . . . . G G G G G . . . . . G G . G G G G G G . G G G G G G G G G G G G G . . . . . . . . . . . . .    
447	G G G G G G G G G G G G G G G G G G G . . G G G G G G G . . . . G G G G G . . . . . G G . G G G G G G . G G G G G G G G G G G G G . . . . . . . . . . . . .    
448	G G G G G G G G G G G G G G G G G G G . . G G G G G G G . . . . G G G G G . . . . . G G . G G G G G G . G G G G G G G G G G G G G . . . . . . . . . . . . .    
449	G G G G G G G G G G G G G G G G G G G . . G G G G G G G . . . . G G G G G . . . . . G G . G G G G G G . G G G G G G G G G G G G G . . . . . . . . . . . . .    
450	A A A A A A A A A A A A A A A A A A A . . A A A A A A A . . . . A A A A A . . . . . A A . A A A A A A . A A A A A A A A A A A A A . . . . . . . . . . . . .    
451	A A A A A A A A A A A A A A A A A A A . . A A A A A A A . . . . A A A A A . . . . . A A . A A A A A A . A A A A A A A A A A A A A . . . . . . . . . . . . .    
452	T T T T T T T T T T T T T T T T T T T . . T T T T T T T . . . . T T T T T . . . . . T T . T T T T T T . T T T T T T T T T T T T T . . . . . . . . . . . . .    
453	C C C C C C C C C C C C C C C C C C C . . C C C C C C C . . . . C C C C C . . . . . C C . C C C C C C . C C C C C C C C C C C C C . . . . . . . . . . . . .    
454	T T T T T T T T T T T T T T T T T T T . . T T T T T T T . . . . T T T T T . . . . . T T . T T T T T T . T T T T T T T T T T T T T . . . . . . . . . . . . .    
455	T T T T T T T T T T T T T T T T T T T . . T T T T T T T . . . . T T T T T . . . . . T T . T T T T T T . T T T T T T T T T T T T T . . . . . . . . . . . . .    
456	G G G G G G G G G G G G G G G G G G G . . G G G G G G G . . . . G G G G G . . . . . G G . G G G G G G . G G G G G G G G G G G G G . . . . . . . . . . . . .    
457	A A A A A A A A A A A A A A A A A A A . . A A A A A A A . . . . A A A A A . . . . . A A . A A A A A A . A A A A A A A A A A A A A . . . . . . . . . . . . .    
458	G G G G G G G G G G G G G G G G G G G . . G G G G G G G . . . . G G G G G . . . . . G G . G G G G G G . G G G G G G G G G G G G G . . . . . . . . . . . . .    
459	G G G G G G G G G G G G G G G G G G G . . G G G G G G G . . . . G G G G G . . . . . G G . G G G G G G . G G G G G G G G G G G G G . . . . . . . . . . . . .    
460	A A A A A A A A A A A A A A A A A A A . . A A A A A A A . . . . A A A A A . . . . . A A . A A A A A A . A A A A A A A A A A A A A . . . . . . . . . . . . .    
461	T T T T T T T T T T T T T T T T T T T . . T T T T T T T . . . . T T T T T . . . . . T T . T T T T T T . T T T T T T T T T T T T T . . . . . . . . . . . . .    
462	C C C C C C C C C C C C C C C C C C C . . C C C C C C C . . . . C C C C C . . . . . C C . C C C C C C . C C C C C C C C C C C C C . . . . . . . . . . . . .    
463	A A A A A A A A A A A A A A A A A A A . . A A A A A A A . . . . A A A A A . . . . . A A . A A A A A A . A A A A A A A A A A A A A . . . . . . . . . . . . .    
464	G G G G G G G G G G G G G G G G G G G . . G G G G G G G . . . . G G G G G . . . . . G G . G G G G G G . G G G G G G G G G G G G G . . . . . . . . . . . . .    
465	A A A A A A A A A A A A A A A A A A A . . A A A A A A A . . . . A A A A A . . . . . A A . A A A A A A . A A A A A A A A A A A A A . . . . . . . . . . . . .    
466	A A A A A A A A A A A A A A A A A A A . . A A A A A A A . . . . A A A A A . . . . . A A . A A A A A A . A A A A A A A A A A A A A . . . . . . . . . . . . .    
467	A A A A A A A A A A A A A A A A A A A . . A A A A A A A . . . . A A A A A . . . . . A A . A A A A A A . A A A A A A A A A A A A A . . . . . . . . . . . . .    
468	T T T T T T T T T T T T T T T T T T T . . T T T T T T T . . . . T T T T T . . . . . T T . T T T T T T . T T T T T T T T T T T T T . . . . . . . . . . . . .    
469	A A A A A A A A A A A A A A A A A A A . . A A A A A A A . . . . A A A A A . . . . . A A . A A A A A A . A A A A A A A A A A A A A . . . . . . . . . . . . .    
470	G G G G G G G G G G G G G G G G G G G . . G G G G G G G . . . . G G G G G . . . . . G G . G G G G G G . G G G G G G G G G G G G G . . . . . . . . . . . . .    
471	A A A A A A A A A A A A A A A A A A A . . A A A A A A A . . . . A A A A A . . . . . A A . A A A A A A . A A A A A A A A A A A A A . . . . . . . . . . . . .    
472	G G G G G G G G G G G G G G G G G G G . . G G G G G G G . . . . G G G G G . . . . . G G . G G G G G G . G G G G G G G G G G G G G . . . . . . . . . . . . .    
473	A A A A A A A A A A A A A A A A A G A . . A A A A A A A . . . . A A A A A . . . . . A A . A A A A G A . A A A A A A A A A A A A A . . . . . . . . . . . . .    
474	T T T T T T T C T T T T T T T T T T T . . T T C T T T T . . . . T T T T T . . . . . T T . T T T T T T . T T T T T T T T T T T T T . . . . . . . . . . . . .    
475	G G G G G G G G G G G G G G G G G G G . . G G G G G G G . . . . G G G G G . . . . . G G . G G G G G G . G G G G G G G G G G G G G . . . . . . . . . . . . .    
476	T T T T T T T T T T T T T T T T T T T . . T T T T T T T . . . . T T T T T . . . . . T T . T T T T T T . T T T T T T T T T T T T T . . . . . . . . . . . . .    
477	A G A A G G A G A G G A A A G G A A A . . A G G G A A G . . . . G G G G G . . . . . G G . G G G G A A . A A G G G G G G G G G G G . . . . . . . . . . . . .  18	 transition  
478	T T T T T T T T T T T T T T T T T T T . . T T T T T T T . . . . T T T T T . . . . . T T . T T T T T T . T T T T T T T T T T T T T . . . . . . . . . . . . .    
479	C C C C C C C C C C C C C C C C C C C . . C C C C C C C . . . . C C C C C . . . . . C C . C C C C C C . C C C C C C C C C C C C C . . . . . . . . . . . . .    
480	T T T T T T T T T T T T T T T T T T T . . T T T T T T T . . . . T T T T T . . . . . T T . T T T T T T . T T T T T T T T T T T T T . . . . . . . . . . . . .    
481	G G G G G G G G G G G G G G G G G G G . . G G G G G G G . . . . G G G G G . . . . . G G . G G G G G G . G G G G G G G G G G G G G . . . . . . . . . . . . .    
482	A A A A A A A A A A A A A A A A A A A . . A A A A A A A . . . . A A A A A . . . . . A A . A A A A A A . A A A A A A A A A A A A A . . . . . . . . . . . . .    
483	C C C C C C C C C C C C C C C C C C C . . C C C C C C C . . . . C C C C C . . . . . C C . C C C C C C . C C C C C C C C C C C C C . . . . . . . . . . . . .    
484	A A A A A A A A A A A A A A A A A A A . . A A A A A A A . . . . A A A A A . . . . . A A . A A A A A A . A A A A A A A A A A A A A . . . . . . . . . . . . .    
485	A A A A A A A A A A A A A A A A A A A . . A A A A A A A . . . . A A A A A . . . . . A A . A A A A A A . A A A A A A A A A A A A A . . . . . . . . . . . . .    
486	A A A A A A A A A A A A A A A A A A A . . A A A A A A A . . . . A A A A A . . . . . A A . A A A A A A . A A A A A A A A A A A A A . . . . . . . . . . . . .    
487	G G G G G G G G G G G G G G G G G G G . . G G G G G G G . . . . G G G G G . . . . . G G . G G G G G G . G G G G G G G G G G G G G . . . . . . . . . . . . .    
488	G G G G G G G G G G G G G G G G G G G . . G G G G G G G . . . . G G G G G . . . . . G G . G G G G G G . G G G G G G G G G G G G G . . . . . . . . . . . . .    
489	G G G G G G G G G G G G G G G G G G G . . G G G G G G G . . . . G G G G G . . . . . G G . G G G G G G . G G G G G G G G G G G G G . . . . . . . . . . . . .    
490	C C C C C C C C C C C C C C C C C C C . . C C C C C C C . . . . C C C C C . . . . . C C . C C C C C C . C C C C C C C C C C C C C . . . . . . . . . . . . .    
491	T T T T T T T T T T T T T T T T T T T . . T T T T T T T . . . . T T T T T . . . . . T T . T T T T T T . T T T T T T T T T T T T T . . . . . . . . . . . . .    
492	G G G G G G G G G G G G G G G G G G G . . G G G G G G G . . . . G G G G G . . . . . G G . G G G G G G . G G G G G G G G G G G G G . . . . . . . . . . . . .    
493	T T T T T T T T T T T T T T T T T T T . . T T T T T T T . . . . T T T T T . . . . . T T . T T T T T T . T T T T T T T T T T T C T . . . . . . . . . . . . .    
494	T T T T T T T T T T T T T T T T T T T . . T T T T T T T . . . . T T T T T . . . . . T T . T T T T T T . T T T T T T T T T T T T T . . . . . . . . . . . . .    
495	A A A A A A A A A A A A A A A A A A A . . A A A A A A A . . . . A A A A A . . . . . A A . A A A A A A . A A A A A A A A A A A A A . . . . . . . . . . . . .    
496	A A A A A A A A A A A A A A A A A A A . . A A A A A A A . . . . A A A A A . . . . . A A . A A A A A A . A A A A A A A A A A A A A . . . . . . . . . . . . .    
497	A A A A A A A A A A A A A A A A A A A . . A A A A A A A . . . . A A A A A . . . . . A A . A A A A A A . A A A A A A A A A A A A A . . . . . . . . . . . . .    
498	G G G G G G G G G G G G G G G G G G G . . G G G G G G G . . . . G G G G G . . . . . G G . G G G G G G . G G G G G G G G G G G G G . . . . . . . . . . . . .    
499	G G G G G G G G G G G G G G G G G G G . . G G G G G G G . . . . G G G G G . . . . . G G . G G G G G G . G G G G G G G G G G G G G . . . . . . . . . . . . .    
500	A A A A A A A A A A A A A A A A A A A . . A A A A A A A . . . . A A A A A . . . . . A A . A A A A A A . A A A A A A A A A A A A A . . . . . . . . . . . . .    
501	A A A A A A A A A A A A A A A A A A A . . A A A A A A A . . . . A A A A A . . . . . A A . A A A A A A . A A A A A A A A A A A A A . . . . . . . . . . . . .    
502	T T T T T T T T T T T T T T T T T T T . . T T T T T T T . . . . T T T T T . . . . . T T . T T T T T T T T T T T T T T T T T T T T . . . . . . . . . . . . .    
503	G G G G G G G G G G G G G G G G G G G . . G G G G G G G . . . . G G G G G . . . . . G G . G G G G G G G G G G G G G G G G G G G G . . . . . . . . . . . . .    
504	A A A A A A A A A A A A A A A A A A A . . A A A A A A A . . . . A A A A A . . . . . A A . A A A A A A A A A A A A A A A A A A A A . . . . . . . . . . . . .    
505	T T T T T T T T T T T T T T T T T T T . . T T T T T T T . . . . T T T T T . . . . . T T . T T T T T T T T T T T T T T T T T T T T . . . . . . . . . . . . .    
506	- - - - - - - - - - - - - - - - - - - . . - A - - - - A . . . . A A - A A . . . . . A - . - - - - - - - - - A A A A A A A A A A A . . . . . . . . . . . . .  18	 indel  
507	- - - - - - - - - - - - - - - - - - - . . - G - - - - G . . . . G G - G G . . . . . G - . - - - - - - - - - G G G G G G G G G G G . . . . . . . . . . . . .  18	 indel  
508	- - - - - - - - - - - - - - - - - - - . . - A - - - - A . . . . A A - A A . . . . . A - . - - - - - - - - - A A A A A A A A A A A . . . . . . . . . . . . .  18	 indel  
509	- - - - - - - - - - - - - - - - - - - . . - G - - - - G . . . . G G - G G . . . . . G - . - - - - - - - - - G G G G G G G G G G G . . . . . . . . . . . . .  18	 indel  
510	- - - - - - - - - - - - - - - - - - - . . - A - - - - A . . . . A A - A A . . . . . A - . - - - - - - - - - A A A A A A A A A A A . . . . . . . . . . . . .  18	 indel  
511	- - - - - - - - - - - - - - - - - - - . . - A - - - - A . . . . A A - A A . . . . . A - . - - - - - - - - - A A A A A A A A A A A . . . . . . . . . . . . .  18	 indel  
512	- - - - - - - - - - - - - - - - - - - . . - T - - - - T . . . . T T - T T . . . . . T - . - - - - - - - - - T T T T T T T T T T T . . . . . . . . . . . . .  18	 indel  
513	- - - - - - - - - - - - - - - - - - - . . - G - - - - G . . . . G G - G G . . . . . G - . - - - - - - - - - G G G G G G G G G G G . . . . . . . . . . . . .  18	 indel  
514	- - - - - - - - - - - - - - - - - - - . . - G - - - - G . . . . G G - G G . . . . . G - . - - - - - - - - - G G G G G G G G G G G . . . . . . . . . . . . .  18	 indel  
515	- - - - - - - - - - - - - - - - - - - . . - T - - - - T . . . . T T - T T . . . . . T - . - - - - - - - - - T T T T T T T T T T T . . . . . . . . . . . . .  18	 indel  
516	A A A A A A A A A A A A A A A A A A A . . A A A A A A A . . . A A A A A A . . . . . A A . A A A A A A A A A A A A A A A A A A A A . . . . . . . . . . . . .    
517	A A A A A A A A A A A A A A A A A A A . . A A A A A A A . . . A A A A A A . . . . . A A . A A A A A A A A A A A A A A A A A A A A . . . . . . . . . . . . .    
518	A A A A A A A A A A A A A A A A A A A . . A C A A A A A . . . A A A A A A . . . . . A A . A A A A A A A A A A A A A A A A A A A A . . . . . . . . . . . . .    
519	G G G G G G G G G G G G G G G G G G G . . G G G G G G G . . . G G G G G G . . . . . G G . G G G G G G G G G G G G G G G G G G G G . . . . . . . . . . . . .    
520	A A A A A A A A A A A A A A A A A A A . . A A A A A A A . . . A A A A A A . . . . . A A . A A A A A A A A A A A A A A A A A A A A . . . . . . . . . . . . .    
521	G G G G G G G G G G G G G G G G G G G . . G G G G G G G . . . G G G G G G . . . G . G G . G G G G G G G G G G G G G G G G G G G G . . . . . . . . . . . . .    
522	G G G G G G G G G G G G G G G G G G G . . G G G G G G G . . . G G G G G G . . . G . G G . G G G G G G G G G G G G G G G G G G G G . . . . . . . . . . . . .    
523	A A A A A A A A G A A A A A A A A A A . . A A A A G A A . . . A A A A A A . . . A . A A . A A A A A A A A A A A A A A A A A A A A . . . . . . . . . . . . .    
524	G G G G G G G G G G G G G G G G G G G . . G G G G G G G . . . G G G G G G . . . G G G G . G G G G G G G G G G G G G G G G G G G G . . . . . . . . . . . . .    
525	G G G G G G G G G G G G G G G G G G G . . G G G G G G G . . . G G G G G G . . . G G G G . G G G G G G G G G G G G G G G G G G G G . . . . . . . . . . . . .    
526	A A A A A A A A A A A A A A A A A A A . . A A A A A A A . . . A A A A A A . . . A A A A . A A A A A A A A A A A A A A A A A A A A . . . . . . . . . . . . .    
527	T T T T T T T T T T T T T T T T T T T . . T T T T T T T . . . T T T T T T . . . T T T T . T T T T T T T T T T T T T T T T T T T T . . . . . . . . . . . . .    
528	A A A A A A A A A A A A A A A A A A A . . A A A A A A A . . . A A A A A A . . . A A A A . A A A A A A A A A A A A A A A A A A A A . . . . . . . . . . . . .    
529	G G G G G G G G G G G G G G G G G G G . . G G G G G G G . . . G G G G G G . . . G G G G . G G G G G G G G G G G G G G G G G G G G . . . . . . . . . . . . .    
530	T T T T T T T T T T T T T T T T T T T . . T T T T T T T . . . T T T T T T . . . T T T T . T T T T T T T T T T T T T T T T T T T T . . . . . . . . . . . . .    
531	A A A A A A A A A A A A A A A A A A A . . A A A A A A A . . . A A A A A A . . . A A A A . A A A A A A A A A A A A A A A A A A A A . . . . . . . . . . . . .    
532	C C C C C C C C C C C C C C C C C C C . . C C C C C C C . . . C C C C C C . . . C C C C . C C C C C C C C C C C C C C C C C C C C . . . . . . . . . . . . .    
533	A A A A A A A A A A A A A A A A A A A . . A A A A A A A . . . A A A A A A . . . A A A A . A A A A A A A A A A A A A A A A A A A A . . . . . . . . . . . . .    
534	A A A A A A A A A A A A A A A A A A A . . A A A A A A A . . . A A A A A A . . . A A A A . A A A A A A A A A A A A A A A A A A A A . . . . . . . . . . . . .    
535	C C C C C C C C C C C C C C C C C C C . . C C C C C C C . . . C C C C C C . . . C C C C . C C C C C C C C C C C C C C C C C C C C . . . . . . . . . . . . .    
536	A A A A A A A A A A A A A A A A A A A . . A A A A A A A . . . A A A A A A . . . A A A A . A A A A A A A A A A A A A A A A A A A A . . . . . . . . . . . . .    
537	A A A A A A A A A A A A A A A A A A A . . A A A A A A A . . . A A A A A A . . . A A A A . A A A A A A A A A A A A A A A A A A A A . . . . . . . . . . . . .    
538	A A A A A A A A A A A A A A A A A A A . . A A A A A A A . . . A A A A A A . . . A A A A . A A A A A A A A A A A A A A A A A A A A . . . . . . . . . . . . .    
539	A A A A A A A A A A A A A A A A A A A . . A A A A A A A . . . A A A A A A . . . A A A A . A A A A A A A A A A A A A A A A A A A A . . . . . . . . . . . . .    
540	T T T T T T T T T T T T T T T T T T T . . T T T T T T T . . . T T T T T T . . . T T T T . T T T T T T T T T T T T T T T T T T T T . . . . . . . . . . . . .    
541	A A A A A A A A A A A A A A A A A A A . . A A A A A A A . . . A A A A A A . . . A A A A . A A A A A A A A A A A A A A A A A A A A . . . . . . . . . . . . .    
542	G G G G G G G G G G G G G G G G G G G . . G G G G G G G . . . G G G G G G . . G G G G G . G G G G G G G G G G G G G G G G G G G G . . . . . . . . . . . . .    
543	G G G G G G G G G G G G G G G G G G G . . G G G G G G G . . . G G G G G G . . G G G G G G G G G G G G G G G G G G G G G G G G G G . . . . . . . . . . . . .    
544	A A A A A A A A A A A A A A A A A A A . . A A A A A A A . . . A A A A A A . . A A A A A A A A A A A A A A A A A A A A A A A A A A . . . . . . . . . . . . .    
545	A A A A A A A A A A A A A A A A A A A . . A A A A A A A . . . A A A A A A . . A A A A A A A A A A A A A A A A A A A A A A A A A A . . . . . . . . . . . . .    
546	A A A A A A A A A A A A A A A A A A A . . A A A A A A A . . . A A A A A A . . A A A A A A A A A A A A A A A A A A A A A A A A A A . . . . . . . . . . . . .    
547	A A A A A A A A A A A A A A A A A A A . . A A A A A A A . . . A A A A A A . . A A A A A A A A A A A A A A A A A A A A A A A A A A . . . . . . . . . . . . .    
548	G G G G G G G G G G G G G G G G G G G . . G G G G G G G . . . G G G G G G . . G G G G G G G G G G G G G G G G G G G G G G G G G G . . . . . . . . . . . . .    
549	A A A A A A A A A A A A A A A A A A A . . T A A A A A A . . . A A A A A A . . A A A A A A A A A A A A A A A A A A A A A A A A A A . . . . . . . . . . . . .    
550	T T T T T T T T T T T T T T T T T T T . . T T T T T T T . . . T T T T T T . . T T T T T T T T T T T T T T T T T T T T T T T T T T . . . . . . . . . . . . .    
551	C C C C C C C C C C C C C C C C C C C . . C C C C C C C . . . C C C C C C . . C C C C C C C C C C C C C C C C C C C C C C C C C C . . . . . . . . . . . . .    
552	T T T T T T T T T T T T T T T T T T T . . T T T T T T T . . . T T T T T T . . T T T T T T T T T T T T T T T T T T T T T T T T T T . . . . . . . . . . . . .    
553	T T T T T T T T T T T T T T T T T T T . . T T T T T T T . T T T T T T T T . . T T T T T T T T T T T T T T T T T T T T T T T T T T . . . . . . . . . . . . .    
554	C C C C C C C C C C C C C C C C C C C . . C C C T C C C . C C T C C T T C . . C C C C C C C T C C C T T C C C C C C C C T C C C C . . . . . . . . . . . . .  8	 transition  
555	T T T T T T T T T T T T T T T T T T T . . T T T T T T T . T T T T T T T T . . T T T T T T T T T T T T T T T T T T T T T T T T T T . . . . . . . . . . . . .    
556	G G G G G G G G G G G G G G G G G G G . G G G G G G G G . G G G G G G G G . . G G G G G G G G G G G G G G G G G G G G G G G G G G . G . . . . . . . . . . .    
557	A A A A A A A A A A A A A A A A A A A . A A A A A A A A . A A A A A A A A . . A A A A A A A A A A A A A A A A A A A A A A A A A A . A . . . . . . . . . . .    
558	A A A A A A A A A A A A A A A A A A A . A A A A A A A A . A A A A A A A A . . A A A A A A A A A A A A A A A A A A A A A A A A A A . A . . . . . . . . . . .    
559	G G G G G G G G G G G G G G G G G G G . G G G G G G G G . G G G G G G G G . . G G G G G G G G G G G G G G G G G G G G G G G G G G . G . . . . . . . . . . .    
560	G G G G G G G G G G G G G G G G G G G . G G G G G G G G . G G G G G G G G . . G G G G G G G G G G G G G G G G G G G G G G G G G G . G . . . . . . . . . . .    
561	A A A A A A A A A A A A A A A A A A A . A A A A A A A A . A A A A A A A A . . A A A A A A A A A A A A A A A A A A A A A A A A A A A A . . . . . . . . . . .    
562	A A A A A A A A A A A A A A A A A A A . A A A A A A A A . A A A A A A A A . . A A A A A A A A A A A A A A A A A A A A A A A A A A A A . . . . . . . . . . .    
563	T T T T T T T T T T T T T T T T T T T . T T T T T T T T . T T T T T T T T . . T T T T T T T T T T T T T T T T T T T T T T T T T T T T . . . . . . . . . . .    
564	A A A A A A A A A A A A A A A A A A A . A A A A A A A A . A A A A A A A A . . A A A A A A A A A A A A A A A A A A A A A A A A A A A A . . . . . . . . . . .    
565	A A A A A A A A A A A A A A A A A A A . A A A A A A A A . A A A A A A A A . . A A A A A A A A A A A A A A A A A A A A A A A A A A A A . . . . . . . . . . .    
566	T T T T T T T T T T T T T T T T T T T . T T T T T T T T . T T T T T T T T . . T T T T T T T T T T T T T T T T T T T T T T T T T T T T . . . . . . . . . . .    
567	C C C C C C C C C C C C C C C C C C C . C C C C C C C C . C C C C C C C C . . C C C C C C C C C C C C C C C C C C C C C C C C C C C C . . . . . . . . . . .    
568	A A A A A A A A A A A A A A A A A A A . A A A A A A A A . A A A A A A A A . . A A A A A A A A A A A A A A A A A A A A A A A A A A A A . . . . . . . . . . .    
569	A A A A A A A A A A A A A A A A A A A . A A A A A A A A . A A A A A A A A . . A A A A A A A A A A A A A A A A A A A A A A A A A A A A . . . . . . . . . . .    
570	A A A A A A A A A A A A A A A A A A A . A A A A A A A A . A A A A A A A A . . A A A A A A A A A A A A A A A A A A A A A A A A A A A A . . . . . . . . . . .    
571	G G G G G G G G G G G G G G G G G G G . G G G G G G G G . G G G G G G G G . . G G G G G G G G G G G G G G G G G G G G G G G G G G G G . . . . . . . . . . .    
572	T T T T T T T T T T T T T T T T T T T . T T T T T T T T . T T T T T T T T . . T T T T T T T T T T T T T T T T T T T T T T T T T T T T . . . . . . . . . . .    
573	G G G G G G G G G G G G G G G G G G G . G G G G G G G G . G G G G G G G G . . G G G G G G G G G G G G G G G G G G G G G G G G G G G G . . . . . . . . . . .    
574	C T T T T T T T T T T T T T T T T T T . T T T T T T T T . T T T T T T T T . . T T T T T T T T T T T T T T T T T T T T T T T T T T T T . . . . . . . . . . .    
575	. G G G G G G G G G G G G G G G G G G . G G G G G G G G . G G G G G G G G . . G G G G G G G G G G G G G G G G G G G G G G G G G G G G . . . . . . . . . . .    
576	. T T T T T T T T T T T T T T T T T T . T T T T T T T T . T T T T T T T T . . T T T T T T T T T T T T T T T T T T T T T T T T T T T T . . . . . . . . . . .    
577	. T T T T T T T T T T T T T T T T T T . T T T T T T T T . T T T T T T T T . . T T T T T T T T T T T T T T T T T T T T T T T T T T T T . . . . . . . . . . .    
578	. T T T T T T T T T T T T T T T T T T . T T T T T T T T . T T T T T T T T . . T T T T T T T T T T T T T T T T T T T T T T T T T T T T . . . . . . . . . . .    
579	. A A A A A A A A A A A A A A A A A A . A A A A A A A A . A A A A A A A A . . A A A A A A A A A A A A A A A A A A A A A A A A A A A A . . . . . . . . . . .    
580	. T T T T T T T T T T T T T T T T T T . T T T T T T T T . T T T T T T T T . . T T T T T T T T T T T T T T T T T T T T T T T T T T T T . . . . . . . . . . .    
581	. T T T T T T T T T T T T T T T T T T . T T T T T T T T . T T T T T T T T . . T T T T T T T T T T T T T T T T T T T T T T T T T T T T . . . . . . . . . . .    
582	. A A A A A A A A A A A A A A A A A A . A A A A A A A A . A A A A A A A A . . A A A A A A A A A A A A A A A A A A A A A A A A A A A A . . . . . . . . . . .    
583	. T T T T T T T T T T T T T T T T T T . T T T T T T T T . T T T T T T T T . . T T T T T T T T T T T T T T T T T T T T T T T T T T T T . . . . . . . . . . .    
584	. A A A A A A A A A A A A A A A A A A . A A A A A A A A . A A A A A A A A . . A A A A A A A A A A A A A A A A A A A A A A A A A A A A . . . . . . . . . . .    
585	. T T T T T T T T T T T T T T T T T T . T T T T T T T T . T T T T T T T T . . T T T T T T T T T T T T T T T T T T T T T T T T T T T T . . . . . . . . . . .    
586	. T T T T T T T T T T T T T T T T T T . T T T T T T T T . T T T T T T T T . . T T T T T T T T T T T T T T T T T T T T T T T T T T T T . . . . . . . . . . .    
587	. G G G G G G G G G G G G G G G G G G . G G G G G G G G . G G G G G G G G . . G G G G G G G G G G G G G G G G G G G G G G G G G G G G . . . . . . . . . . .    
588	. A A A A A A A A A A A A A A A A A A . A A A A A A A A . A A A A A A A A . . A A A A A A A A A A A A A A A A A A A A A A A A A A A A . . . . . . . . . . .    
589	. A A A A A A A A A A A A A A A A A A . A A A A A A A A . A A A A A A A A . . A A A A A A A A A A A A A A A A A A A A A A A A A A A A . . . . . . . . . . .    
590	. A A A A A A A A A A A A A A A A A A . A A A A A A A A . A A A A A A A A . . A A A A A A A A A A A A A A A A A A A A A A A A A A A A . . . . . . . . . . .    
591	. T T T T T T T T T T T T T T T T T T . T T T T T T T T . T T T T T T T T . . T T T T T T T T T T T T T T T T T T T T T T T T T T T T . . . . . . . . . . .    
592	. A A A A A A A A A A A A A A A A A A . A A A A A A A A . A A A A A A A A . . A A A A A A A A A A A A A A A A A A A A A A A A A A A A . . . . . . . . . . .    
593	. T T T T T T T T T T T T T T T T T T . T T T T T T T T . T T T T T T T T . . T T T T T T T T T T T T T T T T T T T T T T T T T T T T . . . . . . . . . . .    
594	. A A A A A A A A A A A A A A A A A A . A A A A A A A A . A A A A A A A A . . A A A A A A A A A A A A A A A A A A A A A A A A A A A A . . . . . . . . . . .    
595	. A A A A A A A A A A A A A A A A A A . A A A A A A A A . A A A A A A A A . . A A A A A A A A A A A A A A A A A A A A A A A A A A A A . . . . . . . . . . .    
596	. A A A A A A A A A A A A A A A A A A . A A A A A A A A . A A A A A A A A . . A A A A A A A A A A A A A A A A A A A A A A A A A A A A . . . . . . . . . . .    
597	. G G G G G G G G G G G G G G G G G G . G G G G G G G G . G G G G G G G G . . G G G G G G G G G G G G G G G G G G G G G G G G G G G G . . . . . . . . . . .    
598	. A A A A A A A A A A A A A A A A A A . A A A A A A A A . A A A A A A A A . . A A A A A A A A A A A A A A A A A A A A A A A A A A A A . . . . . . . . . . .    
599	. T T T T T T T T T T T T T T T T T T . T T T T T T T T . T T T T T T T T . . T T T T T T T T T T T T T T T T T T T T T T T T T T T T . . . . . . . . . . .    
600	. A A A A A A A A A A A A A A A A A A . A A A A A A A A . A A A A A A A A . . A A A A A A A A A A A A A A A A A A A A A A A A A A A A . . . . . . . . . . .    
601	. T T T T T T T T T T T T T T T T T T . T T T T T T T T . T T T T T T T T . . T T T T T T T T T T T T T T T T T T T T T T T T T T T T . . . . . . . . . . .    
602	. T T T T T T T T T T T T T T T T T T . T T T T T T T T . T T T T T T T T . . T T T T T T T T T T T T T T T T T T T T T T T T T T T T . . . . . . . . . . .    
603	. A A A A A A A A A A A A A A A A A A . A A A A A A A A . A A A A A A A A . . A A A A A A A A A A A A A A A A A A A A A A A A A A A A . . . . . . . . . . .    
604	. T T T T T T T T T T T T T T T T T T . T T T T T T T T . T T T T T T T T . . T T T T T T T T T T T T T T T T T T T T T T T T T T T T . . . . . . . . . . .    
605	. A A A A A A A A A A A A A A A A A A . A A A A A A A A . A A A A A A A A . . A A A A A A A A A A A A A A A A A A A A A A A A A A A A . . . . . . . . . . .    
606	. G G G G G G G G G G G G G G G G G G . G G G G G G G G . G G G G G G G G . . G G G G G G G G G G G G G G G G G G G G G G G G G G G G . . . . . . . . . . .    
607	. T T T T T T T T T T T T T T T T T T . T T T T T T T T . T T T T T T T T . . T T T T T T T T T T T T T T T T T T T T T T T T T T T T . . . . . . . . . . .    
608	. T T T T T T T T T T T T T T T T T T . T T T T T T T T . T T T T T T T T . . T T T T T T T T T T T T T T T T T T T T T T T T T T T T . . . . . . . . . . .    
609	. A A A A A A A A A A A A A A A A A A . A A A A A A A A . A A A A A A A A . . A A A A A A A A A A A A A A A A A A A A A A A A A A A A . . . . . . . . . . .    
610	. A A A A A A A A A A A A A A A A A A . A A A A A A A A . A A A A A A A A . . A A A A A A A A A A A A A A A A A A A A A A A A A A A A . . . . . . . . . . .    
611	. A A A A A A A A A A A A A A A A A A . A A A A A A A A . A A A A A A A A . . A A A A A A A A A A A A A A A A A A A A A A A A A A A A . . . . . . . . . . .    
612	. A A A A A A A A A A A A A A A A A A . A A A A A A A A . A A A A A A A A . . A A A A A A A A A A A A A A A A A A A A A A A A A A A A . . . . . . . . . . .    
613	. T T T T T T T T T T T T T T T T T T . T T T T T T T T . T T T T T T T T . . T T T T T T T T T T T T T T T T T T T T T T T T T T T T . . . . . . . . . . .    
614	. G G G G G G G G G G G G G G G G G G . G G G G G G G G . G G G G G G G G . . G G G G G G G G G G G G G G G G G G G G G G G G G G G G . . . . . . . . . . .    
615	. C T T T T T T T T T T T T T T T T T . T T T T T T T T . T T T T T T C T . . T T T T T T T T T C T T T T T T T T T T T T T T T T T T . . . . . . . . . . .    
616	. T T T T T T T T T T T T T T T T T T . T T T T T T T T . T T T T T T T T . . T T T T T T T T T T T T T T T T T T T T T T T T T T T T . . . . . . . . . . .    
617	. A A A A A A A A A A A A A A A A A A . A A A A A A A A . A A A G G A A A . . A A A A A A A A A A A A A A A A A A A A A A A A A A A A . . . . . . . . . . .    
618	. C C C C C C C C C C C C C C C C C C . C C C C C C C C . C C C C C C C C . . C C C C C C C C C C C C C C C C C C C C C C C C C C C C . . . . . . . . . . .    
619	. T T T T T T T T T T T T T T T T T T . T T T T T T T T . T T T T T T T T . . T T T T T T T T T T T T T T T T T T T T T T T T T T T T . . . . . . . . . . .    
620	. A A A A A A A A A A A A A A A A A A . A A A A A A A A . A A A A A A A A . . A A A A A G A A A A A A A A A A A A A A A A A A A A A A . . . . . . . . . . .    
621	. C C C C C C C C C C C C C C C C C C . C C C C C C C C . C C C C C C C C . . C C C C C C C C C C C C C C C C C C C C C C C C C C C C . . . . . . . . . . .    
622	. T T T T T T T T T T T T T T T T T T . T T T T T T T T . T T T T T T T T . . T T T T T T T T T T T T T T T T T T T T T T T T T T T T . . . . . . . . . . .    
623	. G G G G G G G G G G G G G G G G G G . G G G G G G G G . G G G G G G G G . . G G G G G G G G G G G G G G G G G G G G G G G G G G G G . . . . . . . . . . .    
624	. T T T T T T T T T T T T T T T T T T . T T T T T T T T . T T T T T T T T . . T T T T T T T T T T T T T T T T T T T T T T T T T T T T . . . . . . . . . . .    
625	. A A A A A A A A A A A A A A A A A A . A A A A A A A A . A A A A A A A A . . A A A A A A A A A A A A A A A A A A A A A A A A A A A A . . . . . . . . . . .    
626	. A A A A A A A A A A A A A A A A A A . A A A A A A A A . A A A A A A A A . . A A A A A A A A A A A A A A A A A A A A A A A A A A A A . . . . . . . . . . .    
627	. A A A A A A A A A A A A A A A A A A . A A A A A A A A . A A A A A A A A . . A A A A A A A A A A A A A A A A A A A A A A A A A A A A . . . . . . . . . . .    
628	. A A A A A A A A A A A A A A A A A A . A A A A A A A A . A A A A A A A A . . A A A A A A A A A A A A A A A A A A A A A A A A A A A A . . . . . . . . . . .    
629	. A A A A A A A A A A A A A A A A A A . A A A A A A A A . A A A A A A A A . . A A A A A A A A A A A A A A A A A A A A A A A A A A A A . . . . . . . . . . .    
630	. A A A A A A A A A A A A A A A A A A . A A A A A A A A . A A A A A A A A . . A A A A A A A A A A A A A A A A A A A A A A A A A A A A . . . . . . . . . . .    
631	. A A A A A A A A A A A A A A A A A A . A A A A A A A A . A A A A A A A A . . A A A A A A A A A A A A A A A A A A A A A A A A A A A A . . . . . . . . . . .    
632	. A A A A - A - A A A A A A - A A A A . A A A - A A A A . A A A A A A A A . . A A A A A A - - A A A A A A A A A A A A A A A - A A A A . . . . . . . . . . .  7	 indel  
633	. - A - - - - - - - - - - - - - - - - . - - A - - - - - . - - - - - - - - . . - - - - - - - - - - - - - - - - - - - - - - - - - - - - . . . . . . . . . . .    
634	. G G G G G G G G G G G G G G G G G G . G G G G G G G G . G G G G G G G G . . G G G G G G G G G G G G G G G G G G G G G G G G G G G G . . . . . . . . . . .    
635	. A A A A A A A A A A A A A A A A A A . A A A A A A A A . A A A A A A A A . . A A A A A A A A A A A A A A A A A A A A A A A A A A A A . . . . . . . . . . .    
636	. T T T T T T T T T T T T T T T T T T . T T T T T T T T . T T T T T T T T . . T T T T T T T T T T T T T T T T T T T T T T T T T T T T . . . . . . . . . . .    
637	. T T T T T T T T T T T T T T T T T T . T T T T T T T T . T T T T T T T T . . T T T T T T T T T T T T T T T T T T T T T T T T T T T T . . . . . . . . . . .    
638	. T T T T T T T T T T T T T T T T T T . T T T T T T T T . T T T T T T T T . . T T T T T T T T T T T T T T T T T T T T T T T T T T T T . . . . . . . . . . .    
639	. A A A A A A A A A A A A A A A A A A . A A A A A A A A . A A A A A A A A . . A A A A A A A A A A A A A A A A A A A A A A A A A A A A . . . . . . . . . . .    
640	. C C C C C C C C C C C C C C C C C C . C C C C C C C C . C C C C C C C C . . C C C C C C C C C C C C C C C C C C C C C C C C C C C C . . . . . . . . . . .    
641	. A A A A A A A A A A A A A A A A A A . A A A A A A A A . A A A A A A A A . . A A A A A A A A A A A A A A A A A A A A A A A A A A A A . . . . . . . . . . .    
642	. G G G G G G G G G G G G G G G G G G . G G G G G G G G . G G G G G G G G . . G G G G G G G G G G G G G G G G G G G G G G G G G G G G . . . . . . . . . . .    
643	. T T T T T T T T T T T T T T T T T T . T T T T T T T T . T T T T T T T T . . T T T T T T T T T T T T T T T T T T T T T T T T T T T T . . . . . . . . . . .    
644	. A A A A A A A A A A A A A A A A A A . A A A A A A A A . A A A A A A A A . . A A A A A A A A A A A A A A A A A A A A A A A A A A A A . . . . . . . . . . .    
645	. C C C C C C C C C C C C C C C C C C . C C C C C C C C . C C C C C C C C . C C C C C C C C C C C C C C C C C C C C C C C C C C C C C . . . . . . . . . . .    
646	. A A A A A A A A A A A A A A A A A A . A A A A A A A A . A A A A A A A A . A A A A A A A A A A A A A A A A A A A A A A A A A A A A A . . . . . . . . . . .    
647	. T T T T T T T T T T T T T T T T T T . T T T T T T T T . T T T T T T T T . T T T T T T T T T T T T T T T T T T T T T T T T T T T T T . . . . . . . . . . .    
648	. C C C C C C C C C C C C C C C C C C . C C C C C C C C . C C C C C C C C . C C C C C C C C C C C C C C C C C C C C C C C C C C C C C . . . . . . . . . . .    
649	. A A A A A A A A A A A A A A A A A A . A A A A A A A A . A A A A A A A A . A A A A A A A A A A A A A A A A A A A A A A A A A A A A A . . . . . . . . . . .    
650	. A A A A A A A A A A A A A A A A A A . A A A A A A A A . A A A A A A A A . A A A A A A A A A A A A A A A A A A A A A A A A A A A A A . . . . . . . . . . .    
651	. T T T T T T T T T T T T T T T T T T . T T T T T T T T . T T T T T T T T . T T T T T T T T T T T T T T T T T T T T T T T T T T T T T . . . . . . . . . . .    
652	. G G G G G G G G G G G G G G G G G G . G G G G G G G G . G G G G G G G G . G G G G G G G G G G G G G G G G G G G G G G G G G G G G G . . . . . . . . . . .    
653	. A A A A A A A A A A A A A A A A A A . A A A A A A A A . A A A A A A A A . A A A A A A A A A A A A A A A A A A A A A A A A A A A A A . . . . . . . . . . .    
654	. T T T T T T T T T T T T T T T T T T . T T T T T T T T . T T T T T T T T . T T T T T T T T T T T T T T T T T T T T T T T T T T T T T . . . . . . . . . . .    
655	. C C C C C C C C C C C C C C C C C C . C C C C C C C C . C T C C C C T C . C C C C C C C C T C C C C C C C C C C C C C C C C C C C C . . . . . . . . . . .    
656	. T T T T T T T T T T T T T T T T T T . T T T T T T T T . T T T T T T T T . T T T T T T T T T T T T T T T T T T T T T T T T T T T T T . . . . . . . . . . .    
657	. T T T T T T T T T T T T T T T T T T . T T T T T T T T . T T T T T T T T . T T T T T T T T T T T T T T T T T T T T T T T T T T T T T . . . . . . . . . . .    
658	. A A A A A A A A A A A A A A A A A A . A A A A A A A A . A A A A A A A A . A A A A A A A A A A A A A A A A A A A A A A A A A A A A A . . . . . . . . . . .    
659	. A A A A A A A A A A A A A A A A A A . A A A A A A A A . A A A A A A A A . A A A A A A A A A A A A A A A A G A A A A A A A A A A A A . . . . . . . . . . .    
660	. A A A A A A A A A A A A A A A A A A . A A A A A A A A . A A A A A A A A . A A A A A A A A A A A A A A A A A A A A A A A A A A A A A . . . . . . . . . . .    
661	. T T T T T T T T T T T T T T T T T T . T T T T T T T T . T T T T T T T T . T T T T T T T T T T T T T T T T T T T T T T T T T T T T T . . . . . . . . . . .    
662	. G G G G G G G G G G G G G G G G G G . G G G G G G G G . G G G G G G G G . G G G G G G G G G G G G G G G G G G G G G G G G G G G G G . . . . . . . . . . .    
663	. C C C C C C C C C C C C C C C C C C . C C C C C C C C . C C C C C C C C . C C C C C C C C C C C C C C C C C C C C C C C C C C C C C . . . . . . . . . . .    
664	. A A A A A A A A A A A A A A A A A A . A C A A A A A A . A A A A A A A A . A A A A A A A A C A A A A A A A A A A A A A A A A A A A A . . . . . . . . . . .    
665	. C C C C C C C C C C C C C C C C C C . C C C C C C C C . C C C C C C C C . C C C C C C C C C C C C C C C C C C C C C C C C C C C C C . . . . . . . . . . .    
666	. C C C C C C C C C C C C C C C C C C . C C C C C C C C . C C C C C C C C . C C C C C C C C C C C C C C C C C C C C C C C C C C C C C . . . . . . . . . . .    
667	. A A A A C A A A A A A A A A A A A A . A A A A A A A A . A A A A A A A A . A A A A A A A A A A A A A A A A A A A A A A A A A A A A A . . . . . . . . . . .    
668	. T T T T . T T T T T T T T T T T T T . T T T T T T T T . T T T T T T T T . T T T T T T T T T T T T T T T T T T T T T T T T T T T T T . . . . . . . . . . .    
669	. A A A A . A A A A A A A A A A A A A . A A A A A A A A . A A A A A A A A . A A A A A A A A A A A A A A A A A A A A A A A A A A A A A . . . . . . . . . . .    
670	. C C C C . C C C C C C C C C C C C C . C C C C C C C C . C C C C C C C C . C C C C C C C C C C C C C C C C C C C C C C C C C C C C C . . . . . . . . . . .    
671	. A A A A . A A A A A A A A A A A A A . A A A A A A A A . A A A A A A A A . A A A A A A A A A A A A A A A A A A A A A A A A A A A A A . . . . . . . . . . .    
672	. G G G G . G G G G G G G G G G G G G . G G G G G G G G . G G G G G G G G . G G G G G G G G G G G G G G G G G G G G G G G G G G G G G . . . . . . . . . . .    
673	. T T T T . T C T T T T T T T T T T T . T T T C T T T T . T T T T T T T T . T T T T T T T T T T T T T T T T T T T T T T T T T T T T T . . . . . . . . . . .    
674	. C C C C . C C C C C C C C C C C C C . C C C C T C C C . C C C C C C C C . C C C C C C C C C C C C C C C C C C C C C C C C C C C C C . . . . . . . . . . .    
675	. T T T T . T T T T T T T T T T T T T . T T T T T T T T . T T T T T T T T . T T T T T T T T T T T T T T T T T T T T T T T T T T T T T . . . . . . . . . . .    
676	. T T T T . T T T T T T T T T T T T T . T T T T T T T T . T T T T T T T T . T T T T T T T T T T T T T T T T T T T T T T T T T T T T T . . . . . . . . . . .    
677	. A A A A . A A A A A A A A A A A A A . A A A A A A A A . A A A A A A A A . A A A A A A A A A A A A A A A A A A A A A A A A A A A A A . . . . . . . . . . .    
678	. T T T T . T T T T T T T T T T T T T . T T T T T T T T . T T T T T T T T . T T T T T T T T T T T T T T T T T T T T T T T T T T T T T . . . . . . . . . . .    
679	. C C C C . C C C C C C C C C C C C C . C C C C C C C C . C C C C C C C C . C C C C C C C C C C C C C C C C C C C C C C C C C C C C C . . . . . . . . . . .    
680	. T T T T . T T T T T T T T T T T T T . T T T T T T T T . T T T T T T T T . T T T T T T T T T T T T T T T T T T T T T T T T T T T T T . . . . . . . . . . .    
681	. G G G G . G G G G G G G G G G G G G . G G G G G G G G . G G G G G G G G . G G G G G G G G G G G G G G G G G G G G G G G G G G G G G . . . . . . . . . . .    
682	. A A A A . A A A A A A A A A A A A A . A A A A A A A A . A A A A A A A A . A A A A A A A A A A A A A A A A A A A A A A A A A A A A A . . . . . . . . . . .    
683	. T T T T . T T T T T T T T T T T T T T T T T T T T T T . T T T T T T T T . T T T T T T T T T T T T T T T T T T T T T T T T T T T T T . . . . . . . . . . .    
684	. C C C C . C C C C C C C C C C C C C C C C C C C C C C . C C C C C C C C . C C C C C C C C C C C C C C C C C C C C C C C C C C C C C . . . . . . . . . . .    
685	. A A A A . A A A A A A A A A A A A A A A A A A A A A A . A A A A A A A A . A A A A A A A A A A A A A A A A A A A A A A A A A A A A A . . . . . . . . . . .    
686	. T T T T . T T T T T T T T T T T T T T T T T T T T T T . T T T T T T T T . T T T T T T T T T T T T T T T T T T T T T T T T T T T T T . . . . . . . . . . .    
687	. . G G G . G G G G G G G G G G G G G G G G G G G G G G . G G G G G G G G . G G G G G G G G G G G G G G G G G G G G G G G G G G G G G . . . . . . . . . . .    
688	. . C C C . C C C C C C C C C C C C C C C C C C C C C C . C C C C C C C C . C C C C C C C C C C C C C C C C C C C C C C C C C C C C C . . . . . . . . . . .    
689	. . A A A . A A A A A A A A A A A A A A A A A A A A A A . A A A A A A A A . A A A A A A A A A A A A A A A A A A A A A A A A A A A A A . . . . . . . . . . .    
690	. . T T T . T T T T T T T T T T T T T T T T T T T T T T . T T T T T T T T . T T T T T T T T T T T T T T T T T T T T T T T T T T T T T . . . . . . . . . . .    
691	. . A A A . A A A A A A A A A A A A A A A A A A A A A A . A A A A A A A A . A A A A A A A A A A A A A A A A A A A A A A A A A A A A A . . . . . . . . . . .    
692	. . A A A . A A A A A A A A A A A A A A A A A A A A A A . A A A A A A A A . A A A A A A A A A A A A A A A A A A A A A A A A A A A A A . . . . . . . . . . .    
693	. . A A A . A A A A A A A A A A A A A A A A A A A A A A . A A A A A A A A . A A A A A A A A A A A A A A A A A A A A A A A A A A A A A . . . . . . . . . . .    
694	. . T T T . T T T T T T T T T T T T T T T T T T T T T T . T T T T T T T T . T T T T T T T T T T T T T T T T T T T T T T T T T T T T T . . . . . . . . . . .    
695	. . G G G . G G G G G G G G G G G G G G G G G G G G G G . G G G G G G G G . G G G G G G G G G G G G G G G G G G G G G G G G G G G G G G . . . . . . . . . .    
696	. . T T T . T T T T T T T T T T T T T T T T T T T T T T . T T T T T T T T . T T T T T T T T T T T T T T T T T T T T T T T T T T T T T T . . . . . . . . . .    
697	. . T T T . T T T T T T T T T T T T T T T T T T T T T T . T T T T T T T T . T T T T T T T T T T T T T T T T T T T T T T T T T T T T T T . . . . . . . . . .    
698	. . C C C . C C C C C C C C C C C C C C C C C C C C C C . C C C C C C C C . C C C C C C C C C C C C C C C C C C C C C C C C C C C C C C . . . . . . . . . .    
699	. . T T T . T T T T T T T T T T T T T T T T T T T T T T . T T T T T T T T . T T T T T T T T T T T T T T T T T T T T T T T T T T T T T T . . . . . . . . . .    
700	. . T T T . T T T T T T T T T T T T T T T T T T T T T T . T T T T T T T T . T T T T T T T T T T T T T T T T T T T T T T T T T T T T T T . . . . . . . . . .    
701	. . C C C . C C C C C C C C C C C C C C C C C C C C C C . C C C C C C C C . C C C C C C C C C C C C C C C C C C C C C C C C C C C C C C . . . . . . . . . .    
702	. . A A C . A A A A A A A A A A A A A A A A A A A A A A . A A A A A A A A . A A A A A A A A A A A A A A A A A A A A A A A A A A A A A A . . . . . . . . . .    
703	. . T T . . T T T T T T T T T T T T T T T T T T T T T T . T T T T T T T T . T T T T T T T T T T T T T T T T T T T T T T T T T T T T T T . . . . . . . . . .    
704	. . A A . . A A A A A A A A A A A A A A A A A A A A A A . A A A A A A A A . A A A A A A A A A A A A A A A A A A A A A A A A A A A A A A . . . . . . . . . .    
705	. . . T . . T T T T T T T T T T T T T T T T T T T T T T . T T T T T T T T . T T T T T T T T T T T T T T T T T T T T T T T T T T T T T T . . . . . . . . . .    
706	. . . . . . T T T T T T T T T T T T T T T T T T T T T T . T T T T T T T T . T T T T T T T T T T T T T T T T T T T T T T T T T T T T T T . . . . . . . . . .    
707	. . . . . . T T T T T T T T T T T T T T T T T T T T T T . T T T T T T T T . T T T T T T T T T T T T T T T T T T T T T T T T T T T T T T . . . . . . . . . .    
708	. . . . . . A A T A T A A A A A A A A A A A A A A A A A . A A A A A A A A . A A A A A A A A A A A A A A A A A A A A A A A A A A A A A A . . . . . . . . . .    
709	. . . . . . A A A A A A A A A A A A A A A A A A A A A A . A A A A A A A A . A A A A A A A A A A A A A A A A A A A A A A A A A A A A A A . . . . . . . . . .    
710	. . . . . . A A A A A A A A A A A A A A A A A A A A A A . A A A A A A A A . A A A A A A A A A A A A A A A A A A A A A A A A A A A A A A . . . . . . . . . .    
711	. . . . . . G G G G G G G G G G G G G G G G G G G G G G . G G G G G G G G . G G G G G G G G G G G G G G G G G G G G G G G G G G G G G G . . . . . . . . . .    
712	. . . . . . G G G G G G G G G G G G G G G G G G G G G G . G G G G G G G G . G G G G G G G G G G G G G G G G G G G G G G G G G G G G G G . . . . . . . . . .    
713	. . . . . . G G G G G G G G G G G G G G G G G G G G G G . G G G G G G G G . G G G G G G G G G G G G G G G G G G G G G G G G G G G G G G . . . . . . . . . .    
714	. . . . . . T T T T T T T T T T T T T T T T T T T T T T . T T T T T T T T . T T T T T T T T T T T T T T T T T T T T T T T T T T T T T T . . . . . . . . . .    
715	. . . . . . A A A A A A A A A A A A A A A A A A A A A A . A A A A A A A A . A A A A A A A A A A A A A A A A A A A A A A A A A A A A A A . . . . . . . . . .    
716	. . . . . . T T T T T T T T T T T T T T T T T T T T T T . T T T T T T T T . T T T T T T T T T T T T T T T T T T T T T T T T T T T T T T . . . . . . . . . .    
717	. . . . . . A A A A A A A A A A A A A A A A A A A A A A . A A A A A A A A . A A A A A A A A A A A A A A A A A A A A A A A A A A A A A A . . . . . . . . . .    
718	. . . . . . C C C C C C C C C C C C C C C C C C C C C C . C C C C C C C C . C C C C C C C C C C C C C C C C C C C C C C C C C C C C C C . . . . . . . . . .    
719	. . . . . . A A A A A A A A A A A A A A A A A A A A A A . A A A A A A A A . A A A A A A A A A A A A A A A A A A A A A A A A A A A A A A . . . . . . . . . .    
720	. . . . . . G G G G G G G G G G G G G G G G G G G G G G . G G G G G G G G G G G G G G G G G G G G G G G G G G G G G G G G G G G G G G G . . . . . . . . . .    
721	. . . . . . A A A A A A A A A A A A A A A A A A A A A A . A A A A A A A A A A A A A A A A A A A A A A A A A A A A A A A A A A A A A A A . . . . . . . . . .    
722	. . . . . . A A A A A A A A A A A A A A A A A A A A A A . A A A A A A A A A A A A A A A A A A A A A A A A A A A A A A A A A A A A A A A . . . . . . . . . .    
723	. . . . . . A A A A A A A A A A A A A A A A A A A A A A . A A A A A A A A A A A A A A A A A A A A A A A A A A A A A A A A A A A A A A A . . . . . . . . . .    
724	. . . . . . T T T T T T T T T T T T T T T T T T T T T T . T T T T T T T T T T T T T T T T T T T T T T T T T T T T T T T T T T T T T T T . . . . . . . . . .    
725	. . . . . . T C C C C C C C C C C C C C C C C C C C C C . C C C C C C C C C C C C C C C C C C C C C C C C C C C C C C C C C C C C C C C . . . . . . . . . .    
726	. . . . . . . A A A A A A A A A A A A A A A A A A A A A . A A A A A A A A A A A A A A A A A A A A A A A A A A A A A A A A A A A A A A A . . . . . . . . . .    
727	. . . . . . . A A A A A A A A A A A A A A A A A A A A A . A A A A A A A A A A A A A A A A A A A A A A A A A A A A A A A A A A A A A A A . . . . . . . . . .    
728	. . . . . . . T T T T T T T T T T T T T T T T T T T T T . T T T T T T T T T T T T T T T T T T T T T T T T T T T T T T T T T T T T T T T . . . . . . . . . .    
729	. . . . . . . A A A A A A A A A A A A A A A A A A A A A . A A A A A A A A A A A A A A A A A A A A A A A A A A A A A A A A A A A A A A A . . . . . . . . . .    
730	. . . . . . . C C C C C C C C C C C C C C C C C C C C C . C C C C C C C C C C C C C C C C C C C C C C C C C C C C C C C C C C C C C C C . . . . . . . . . .    
731	. . . . . . . A A A A A A A A A A A A A A A A A A A A A . A A A A A A A A A A A A A A A A A A A A A A A A A A A A A A A A A A A A A A A . . . . . . . . . .    
732	. . . . . . . A A A A A A A A A A A A A A A A A A A A A . A A A A A A A A A A A A A A A A A A A A A A A A A A A A A A A A A A A A A A A . . . . . . . . . .    
733	. . . . . . . A A A A A A A A A A A A A A A A A A A A A . A A A A A A A A A A A A A A A A A A A A A A A A A A A A A A A A A A A A A A A . . . . . . . . . .    
734	. . . . . . . T T T T T T T T T T T T T T T T T T T T T . T T T T T T T T T T T T T T T T T T T T T T T T T T T T T T T T T T T T T T T . . . . . . . . . .    
735	. . . . . . . T T T T T T T T T T T T T T T T T T T T T . T T T T T T T T T T T T T T T T T T T T T T T T T T T T T T T T T T T T T T T . . . . . . . . . .    
736	. . . . . . . A A A A A A A A A A A A A A A A A A A A A . A A A A A A A A A A A A A A A A A A A A A A A A A A A A A A A A A A A A A A A . . . . . . . . . .    
737	. . . . . . . A A A A A A A A A A A A A A A A A A A A A . A A A A A A A A A A A A A A A A A A A A A A A A A A A A A A A A A A A A A A A . . . . . . . . . .    
738	. . . . . . . T T T T T T T T T T T T T T T T T T T T T . T T T T T T T T T T T T T T T T T T T T T T T T T T T T T T T T T T T T T T T . . . . . . . . . .    
739	. . . . . . . A A A A A A A A A A A A A A A A A A A A A . A A A A A A A A A A A A A A A A A A A A A A A A A A A A A A A A A A A A A A A . . . . . . . . . .    
740	. . . . . . . A A A A A A A A A A A A A A A A A A A A A . A A A A A A A A A A A A A A A A A A A A A A A A A A A A A A A A A A A A A A A . . . . . . . . . .    
741	. . . . . . . G G G G G G G G G G G G G G G G G G G G G . G G G G G G G G G G G G G G G G G G G G G G G G G G G G G G G G G G G G G G G . . . . . . . . . .    
742	. . . . . . . A A A A A A A A A A A A A A A A A A A A A . A A A A A A A A A A A A A A A A G A A A A A A A A A A A A A A A A A A A A A A . . . . . . . . . .    
743	. . . . . . . T T T T T T T T T T T T T T T T T T T T T . T T T T T T T T T T T T T T T T T T T T T T T T T T T T T T T T T T T T T T T . . . . . . . . . .    
744	. . . . . . . C C C C C C C C C C C C C C C C C C C C C . C C C C C C C C C C C C C C C C C C C C C C C C C C C C C C C C C C C C C C C . . . . . . . . . .    
745	. . . . . . . A A A A A A A A A A A A A A A A A A A A A . A A A A A A A A A A A A A A A A A A A A A A A A A A A A A A A A A A A A A A A . . . . . . . . . .    
746	. . . . . . . G G G G G G G G G G G G G G G G G G G G G . G G G G G G G G G G G G G G G G G G G G G G G G G G G G G G G G G G G G G G G . . . . . . . . . .    
747	. . . . . . . C C C C C C C C C C C C C C C C C C C C C . C C C C C C C C C C C C C C C C C C C C C C C C C C C C C C C C C C C C C C C . . . . . . . . . .    
748	. . . . . . . T T T T T T T T T T T T T T T T T T T T T . T T T T T T T T T T T T T T T T T T T T T T T T T T T T T T T T T T T T T T T . . . . . . . . . .    
749	. . . . . . . G G G G G G G A G G G G G G G G G G G G G . G G G G G G G G G G G G G G G G G A G G G G G G G G G G G G G G G G G G G G G . . . . . . . . . .    
750	. . . . . . . G G G G G G G G G G G G G G G G G G G G G . G G G G G G G G G G G G G G G G G G G G G G G G G G G G G G G G G G G G G G G . . . . . . . . . .    
751	. . . . . . . T T T T T G T T T T T T T T T T T T T T T . T T T T T T T T T T T T T T T T T T T T T T T T T T T T T T T T T T T T T T T . . . . . . . . . .    
752	. . . . . . . T T T T T T T T T T T T T T T T T T T T T . T T T T T T T T T T T T T T T T T T T T T T T T T T T T T T T T T T T T T T T . . . . . . . . . .    
753	. . . . . . . C C C C C C C C C C C C C C C C C C C C C . C C C C C C C C C C C C C C C C C C C C C C C C C C C C C C C C C C C C C C C . . . . . . . . . .    
754	. . . . . . . C C C C C C C C C C C C C C C C C C C C C . C C C C C C C C C C C C C C C C C C C C C C C C C C C C C C C C C C C C C C C . . . . . . . . . .    
755	. . . . . . . T T T T T T T C T T T T T T T T T T T T T . T T T T T T T T T T T T T T C C T C T T T T T T T T T T T T T T T T T T T T T . . . . . . . . . .    
756	. . . . . . . A A A A A A A A A A A A A A A A A A A A A . A A A A A A A A A A A A A A A A A A A A A A A A A A A A A A A A A A A A A A A . . . . . . . . . .    
757	. . . . . . . T T T T T T T T T T T T T T T T T T T T T . T T T T T T T T T T T T T T T T T T T T T T T T T T T T T T T T T T T T T T T . . . . . . . . . .    
758	. . . . . . . C C C C C C C C C C C C T C C C C C C C C . C C C C C C C C C C C C C C C C C C C C C C C C C C C C C C C C C C C C C C C . . . . . . . . . .    
759	. . . . . . . T T T T T T T T T T T T T T T T T T T T T . T T T T T T T T T T T T T T T T T T T T T T T T T T T T T T T T T T T T T T T . . . . . . . . . .    
760	. . . . . . . C C C C C C C C C C C C C C C C C C C C C . C C C C C C C C C C C C C C C C C C C C C C C C C C C C C C C C C C C C C C C . . . . . . . . . .    
761	. . . . . . . G G G G G G G G G G G G G G G G G G G G G . G G G G G G G G G G G G G G G G G G G G G G G G G G G G G G G G G G G G G G G . . . . . . . . . .    
762	. . . . . . . T A T T T T T T T T T T T T T T T T T T T . T T T T T T T T T T T T T T T T T T T T T T T T T T T T T T T T T T T T T T T . . . . . . . . . .    
763	. . . . . . . A . A A A A A A A A A A A A A A A A A A A . A A A A A A A A A A A A A A A A A A A A A A A A A A A A A A A A A A A A A A A . . . . . . . . . .    
764	. . . . . . . T . T T T T T T T T T T T T T T T T T T T . T T T T T T T T T T T T T T T T T T T T T T T T T T T T T T T T T T T T T T T . . . . . . . . . .    
765	. . . . . . . T . T T T T T T T T T T T T T T T T T T T . T T T T T T T T T T T T T T T T T T T T T T T T T T T T T T T T T T T T T T T . . . . . . . . . .    
766	. . . . . . . T . T T T T T T T T T T T T T T T T T T T . T T T T T T T T T T T T T T T T T T T T T T T T T T T T T T T T T T T T T T T . . . . . . . . . .    
767	. . . . . . . G . G G G G G G G G G G G G G G G G G G G . G G G G G G G G G G G G G G G G G G G G G G G G G G G G G G G G G G G G G G G . . . . . . . . . .    
768	. . . . . . . A . A G A A A A A A A A A A A A A A A A A . A A A A A A A A A A A A A A A A A A A A A A A A A A A A A A A A A A A A A A A . . . . . . . . . .    
769	. . . . . . . T . T . T T T T T T T T T T T T T T T T T . T T T T T T T T T T T T T T T T T T T T T T T T T T T T T T T T T T T T T T T . . . . . . . . . .    
770	. . . . . . . G . G . G G G G G G G G G G G G G G G G G . G G G G G G G G G G G G G G G G G G G G G G G G G G G G G G G G G G G G G G G . . . . . . . . . .    
771	. . . . . . . A . A . A A A A A A A A A A A A A A A A A . A A A A A A A A A A A A A A A A A A A A A A A A A A A A A A A A A A A A A A A . . . . . . . . . .    
772	. . . . . . . T . T . T T T T T T T T T T T T T T T T T . T T T T T T T T T T T T T T T T T T T T T T T T T T T T T T T T T T T T T T T . . . . . . . . . .    
773	. . . . . . . . . . . C C C C C C C C C C C C C C C C C . C C C C C C C C C C C C C C C C C C C C C C C C C C C C C C C C C C C C C C C . . . . . . . . . .    
774	. . . . . . . . . . . A A A A A A A A A A A A A A A A A . A A A A A A A A A A A A A A A A A A A A A A A A A A A A A A A A A A A A A A A . . . . . . . . . .    
775	. . . . . . . . . . . A A A A A A A A A A A A A A A A A . A A A A A A A A A A A A A A A A A A A A A A A A A A A A A A A A A A A A A A A . . . . . . . . . .    
776	. . . . . . . . . . . T T T T T T T T T T T T T T T T T . T T T T T T T T T T T T T T T T T T T T T T T T T T T T T T T T T T T T T T T . . . . . . . . . .    
777	. . . . . . . . . . . A A A A A A A A A A A A A A A A A . A A A A A A A A A A A A A A A A A A A A A A A A A A A A A A A A A A A A A A A . . . . . . . . . .    
778	. . . . . . . . . . . T T T T T T T T T T T T T T T T T . T T T T T T T T T T T T T T T T T T T T T T T T T T T T T T T T T T T T T T T . . . . . . . . . .    
779	. . . . . . . . . . . T A A A A A A A A A A A A A A A A . A A A A A A A A A A A A A A A A A A A A A A A A A A A A A A A A A A A A A A A . . . . . . . . . .    
780	. . . . . . . . . . . . T T T T T T T T T T T T T T T T . T T T T T T T T T T T T T T T T T T T T T T T T T T T T T T T T T T T T T T T . . . . . . . . . .    
781	. . . . . . . . . . . . T T T T T T T T T T T T T T T T . T T T T T T T T T T T T T T T T T T T T T T T T T T T T T T T T T T T T T T T . . . . . . . . . .    
782	. . . . . . . . . . . . C C C C C C C C C C C C C C C C . C C C C C C C C C C C C C C C C C C C C C C C C C C C C C C C C C C C C C C C . . . . . . . . . .    
783	. . . . . . . . . . . . T T T T T T T T T T T T T T T T . T T T T T T T T T T T T T T T T T T T T T T T T T T T T T T T T T T T T T T T . . . . . . . . . .    
784	. . . . . . . . . . . . T T T T T T T T T T T T T T T T . T T T T T T T T T T T T T T T T T T T T T T T T T T T T T T T T T T T T T T T . . . . . . . . . .    
785	. . . . . . . . . . . . T T T T T T T T T T T T T T T T . T T T T T T T T T T T T T T T T T T T T T T T T T T T T T T T T T T T T T T T . . . . . . . . . .    
786	. . . . . . . . . . . . C C . C C C C C C C C C C C C C . C C C C C C C C C C C C C C C C C C C C C C C C C C C C C C C C C C C C C C C . . . . . . . . . .    
787	. . . . . . . . . . . . A A . A A A A A A A A A A A A A . A A A A A A A A A A A A A A A A A A A A A A A A A A A A A A A A A A A A A A A . . . . . . . . . .    
788	. . . . . . . . . . . . G G . G G G G G G G G G G G G G . G G G G G G G G G G G G G G G G G G G G G G G G G G G G G G G G G G G G G G G . . . . . . . . . .    
789	. . . . . . . . . . . . C C . C C C C C C C C C C C C C . C C C C C C C C C C C C C C C C C C C C C C C C C C C C C C C C C C C C C C C . . . . . . . . . .    
790	. . . . . . . . . . . . C C . C C C C C C C C C C C C C . C C C C C C C C C C C C C C C C C C C C C C C C C C C C C C C C C C C C C C C . . . . . . . . . .    
791	. . . . . . . . . . . . T T . T T T T T T T T T T T T T . T T T T T T T T T T T T T T T T T T T T T T T T T T T T T T T T T T T T T T T . . . . . . . . . .    
792	. . . . . . . . . . . . T T . T T T T T T T T T T T T T . T T T T T T T T T T T T T T T T T T T T T T T T T T T T T T T T T T T T T T T . . . . . . . . . .    
793	. . . . . . . . . . . . C C . C C C C C C C C C C C C C . C C C C C C C C C C C C C C C C C C C C C C C C C C C C C C C C C C C C C C C . . . . . . . . . .    
794	. . . . . . . . . . . . A A . A A A A A A A A A A A A A . A A A A A A A A A A A A A A A A A A A A A A A A A A A A A A A A A A A A A A A . . . . . . . . . .    
795	. . . . . . . . . . . . A A . A A A A A A A A A A A A A . A A A A A A A A A A A A A A A A A A A A A A A A A A A A A A A A A A A A A A A . . . . . . . . . .    
796	. . . . . . . . . . . . T T . T T T T T T T T T T T T T . T T T T T T T T T T T T T T T T T T T T T T T T T T T T T T T T T T T T T T T . . . . . . . . . .    
797	. . . . . . . . . . . . A A . A A A A A A A A A A A A A . A A A A A A A A A A A A A A A A A A A A A A A A A A A A A A A A A A A A A A A . . . . . . . . . .    
798	. . . . . . . . . . . . T T . T T T T T T T T T T T T T . T T T T T T T T T T T T T T T T T T T T T T T T T T T T T T T T T T T T T T T . . . . . . . . . .    
799	. . . . . . . . . . . . G G . G G G G G G G G G G G G G . G G G G G G G G G G G G G G G G G G G G G G G G G G G G G G G G G G G G G G G . . . . . . . . . .    
800	. . . . . . . . . . . . A A . A A A A A A A A A A A A A . A A A A A A A A A A A A A A A A A A A A A A A A A A A A A A A A A A A A A A A . . . . . . . . . .    
801	. . . . . . . . . . . . T T . T T T T T T T T T T T T T . T T T T T T T T T T T T T T T T T T T T T T T T T T T T T T T T T T T T T T T . . . . . . . . . .    
802	. . . . . . . . . . . . C C . C C C C C C C C C C C C C . C C C C C C C C C C C C C C C C C C C C C C C C C C C C C C C C C C C C C C C . . . . . . . . . .    
803	. . . . . . . . . . . . A A . A A A A A A A A A A A A A . A A A A A A A A A A A A A A A A A A A A A A A A A A A A A A A A A A A A A A A . . . . . . . . . .    
804	. . . . . . . . . . . . T T . T T T T T T T T T T T T T T T T T T T T T T T T T T T T T T T T T T T T T T T T T T T T T T T T T T T T T . . . . . . . . . .    
805	. . . . . . . . . . . . G G . G G G G G G G G G G G G G G G G G G G G G G G G G G G G G G G G G G G G G G G G G G G G G G G G G G G G G . . . . . . . . . .    
806	. . . . . . . . . . . . T T . T T T T T T T T T T T T T T T T T T T T T T T T T T T T T T T T T T T T T T T T T T T T T T T T T T T T T . . . . . . . . . .    
807	. . . . . . . . . . . . G . . G G G G G G G G G G G G G G G G G G G G G G G G G G G G G G G G G G G G G G G G G G G G G G G G G G G G G . . . . . . . . . .    
808	. . . . . . . . . . . . G . . G G G G G G G G G G G G G G G G G G G G G G G G G G G G G G G G G G G G G G G G G G G G G G G G G G G G G . . . . . . . . . .    
809	. . . . . . . . . . . . T . . T T T T T T T T T T T T T T T T T T T T T T T T T T T T T T T T T T T T T T T T T T T T T T T T T T T T T . . . . . . . . . .    
810	. . . . . . . . . . . . G . . G G G G G G G G G G G G G G G G G G G G G G G G G G G G G G G G G G G G G G G G G G G G G G G G G G G G G . . . . . . . . . .    
811	. . . . . . . . . . . . C . . C C C C C C C C C C C C C C C C C C C C C C C C C C C C C C C C C C C C C C C C C C C C C C C C C C C C C . . . . . . . . . .    
812	. . . . . . . . . . . . A . . A A A A A A A A A A A A A A A A A A A A A A A A A A A A A A A A A A A A A A A A A A A A A A A A A A A A A . . . . . . . . . .    
813	. . . . . . . . . . . . T . . T T T T T T T T T T T T T T T T T T T T T T T T T T T T T T T T T T T T T T T T T T T T T T T T T T T T T . . . . . . . . . .    
814	. . . . . . . . . . . . A . . A A A A A A A A A A A A A A A A A A A A A A A A A A A A A A A A A A A A A A A A A A A A A A A A A A A A A . . . . . . . . . .    
815	. . . . . . . . . . . . T . . T T T T T T T T T T T T T T T T T T T T T T T T T T T T T T T T T T T T T T T T T T T T T T T T T T T T T . . . . . . . . . .    
816	. . . . . . . . . . . . . . . T T T T T T T T T T T T T T T T T T T T T T T T T T T T T T T T T T T T T T T T T T T T T T T T T T T T T . . . . . . . . . .    
817	. . . . . . . . . . . . . . . G G G G G G G G G G G G G G G G G G G G G G G G G G G G G G G G G G G G G G G G G G G G G G G G G G G G G . . . . . . . . . .    
818	. . . . . . . . . . . . . . . A A A A A A A A A A A A A A A A A A A A A A A A A A A A A A A A A A A A A A A A A A A A A A A A A A A A A . . . . . . . . . .    
819	. . . . . . . . . . . . . . . T T T T T T T T T T T T T T T T T T T T T T T T T T T T T T T T T T T T T T T T T T T T T T T T T T T T T . . . . . . . . . .    
820	. . . . . . . . . . . . . . . A A A A A A A A A A A A A A A A A A A A A A A A A A A A A A A A A A A A A A A A A A A A A A A A A A A A A . . . . . . . . . .    
821	. . . . . . . . . . . . . . . A A A A A A A A A A A A A A A A A A A A A A A A A A A A A A A A A A A A A A A A A A A A A A A A A A A A A . . . . . . . . . .    
822	. . . . . . . . . . . . . . . T T T T T T T T T T T T T T T T T T T T T T T T T T T T T T T T T T T T T T T T T T T T T T T T T T T T T . . . . . . . . . .    
823	. . . . . . . . . . . . . . . G G G G G G G G G G G G G G G G G G G G G G G G G G G G G G G G G G G G G G G G G G G G G G G G G G G G G . . . . . . . . . .    
824	. . . . . . . . . . . . . . . C C C C C C C C C C C C C C C C C C C C C C C C C C C C C C C C C C C C C C C C C C C C C C C C C C C C C . . . . . . . . . .    
825	. . . . . . . . . . . . . . . A A A A A A A A A A A A A A A A A A A A A A A A A A A A A A A A A A A A A A A A A A A A A A A A A A A A A . . . . . . . . . .    
826	. . . . . . . . . . . . . . . C C C C C C C C C C C C C C C C C C C C C C C C C C C C C C C C C C C C C C C C C C C C C C C C C C C C C . . . . . . . . . .    
827	. . . . . . . . . . . . . . . A A A A A A A A A A A A A A A A A A A A A A A A A A A A A A A A A A A A A A A A A A A A A A A A A A A A A . . . . . . . . . .    
828	. . . . . . . . . . . . . . . A A A A A A A A A A A A A A A A A A A A A A A A A A A A A A A A A A A A A A A A A A A A A A A A A A A A A . . . . . . . . . .    
829	. . . . . . . . . . . . . . . A A A A A A A A A A A A A A A A A A A A A A A A A A A A A A A A A A A A A A A A A A A A A A A A A A A A A . . . . . . . . . .    
830	. . . . . . . . . . . . . . . T T T T T T T T T T T T T T T T T T T T T T T T T T T T T T T T T T T T T T T T T T T T T T T T T T T T T . . . . . . . . . .    
831	. . . . . . . . . . . . . . . T T T T T T T T T T T T T T T T T T T T T T T T T T T T T T T T T T T T T T T T T T T T T T T T T T T T T . . . . . . . . . .    
832	. . . . . . . . . . . . . . . G G G G G G G G G G G G G G G G G G G G G G G G G G G G G G G G G G G G G G G G G G G G G G G G G G G G G . . . . . . . . . .    
833	. . . . . . . . . . . . . . . T T T T T T T T T T T T T T T T T T T T T T T T T T T T T T T T T T T T T T T T T T T T T T T T T T T T T . . . . . . . . . .    
834	. . . . . . . . . . . . . . . A A A A A A A A A A A A A A A A A A A A A A A A A A A A A A A A A A A A A A A A A A A A A A A A A A A A A . . . . . . . . . .    
835	. . . . . . . . . . . . . . . T T T T T T T T T T T T T T T T T T T T T T T T T T T T T T T T T T T T T T T T T T T T T T T T T T T T T . . . . . . . . . .    
836	. . . . . . . . . . . . . . . T T T T T T T T T T T T T T T T T T T T T T T T T T T T T T T T T T T T T T T T T T T T T T T T T T T T T . . . . . . . . . .    
837	. . . . . . . . . . . . . . . T T T T T T T T T T T T T T T T T T T T T T T T T T T T T T T T T T T T T T T T T T T T T T T T T T T T T . . . . . . . . . .    
838	. . . . . . . . . . . . . . . G G G G G G G G G G G G G G G G G G G G G G G G G G G G G G G G G G G G G G G G G G G G G G G G G G G G G . . . . . . . . . .    
839	. . . . . . . . . . . . . . . A A A A A A A A A A A A A A A A A A A A A A A A A A A A A A A A A A A A A A A A A A A A A A A A A A A A A . . . . . . . . . .    
840	. . . . . . . . . . . . . . . A A A A A A A A A A A A A A A A A A A A A A A A A A A A A A A A A A A A A A A A A A A A A A A A A A A A A . . . . . . . . . .    
841	. . . . . . . . . . . . . . . A A A A A A A A A A A A A A A A A A A A A A A A A A A A A A A A A A A A A A A A A A A A A A A A A A A A A . . . . . . . . . .    
842	. . . . . . . . . . . . . . . G G G G G G G G G G G G G G G G G G G G G G G G G G G G G G G G G G G G G G G G G G G G G G G G G G G G G . . . . . . . . . .    
843	. . . . . . . . . . . . . . . A A A A A A A A A A A A A A A A A A A A A A A A A A A A A A A A A A A A A A A A A A A A A A A A A A A A A . . . . . . . . . .    
844	. . . . . . . . . . . . . . . T T T T T T T T C T T T T T T T T T T T T T T T T T T T T T T T T T T T T T T T T T T T T T T T T T T T T . . . . . . . . . .    
845	. . . . . . . . . . . . . . . C C C C C C C C C C C C C C C C C C C C C C C C C C C C C C C C C C C C C C C C C C C C C C C C C C C C C . . . . . . . . . .    
846	. . . . . . . . . . . . . . . C C C C C C C C C C C C C C C C C C C C C C C C C C C C C C C C C C C C C C C C C C C C C C C C C C C C C . . . . . . . . . .    
847	. . . . . . . . . . . . . . . A A A C A A A A A A A A A A A A A A A A A A A A A A A A A A A A A A A A A A A A A A A A A A A A A A A A A . . . . . . . . . .    
848	. . . . . . . . . . . . . . . A A A . A A A A A A A A A A A A A A A A A A A A A A A A A A A A A A A A A A A A A A A A A A A A A A A A A . . . . . . . . . .    
849	. . . . . . . . . . . . . . . T T T . T T T T T T T T T T T T T T T T T T T T T T T T T T T T T T T T T T T T T T T T T T T T T T T T T . . . . . . . . . .    
850	. . . . . . . . . . . . . . . A A A . A A A A A A A A A A A A A A A A A A A A A A A A A A A A A A A A A A A A A A A A A A A A A A A A A . . . . . . . . . .    
851	. . . . . . . . . . . . . . . T T T . T T T T T T T T T T T T T T T T T T T T T T T T T T T T T T T T T T T T T T T T T T T T T T T T T . . . . . . . . . .    
852	. . . . . . . . . . . . . . . G G G . G G G G G G G G G G G G G G G G G G G G G G G G G G G G G G G G G G G G G G G G G G G G G G G G G . . . . . . . . . .    
853	. . . . . . . . . . . . . . . T T T . T T T T T T T T T T T T T T T T T T T T T T T T T T T T T T T T T T T T T T T T T T T T T T T T T . . . . . . . . . .    
854	. . . . . . . . . . . . . . . G G G . G G G G G G G G G G G G G G G G G G G G G G G G G G G G G G G G G G G G G G G G G G G G G G G G G . . . . . . . . . .    
855	. . . . . . . . . . . . . . . C C C . C C C C C C C C C C C C C C C C C C C C C C C C C C C C C C C C C C C C C C C C C C C C C C C C C . . . . . . . . . .    
856	. . . . . . . . . . . . . . . A A A . A A A A A A A A A A A A A A A A A A A A A A A A A A A A A A A A A A A A A A A A A A A A A A A A A . . . . . . . . . .    
857	. . . . . . . . . . . . . . . C C C . C C C C C C C C C C C C C C C C C C C C C C C C C C C C C C C C C C C C C C C C C C C C C C C C C . . . . . . . . . .    
858	. . . . . . . . . . . . . . . T T T . T T T T T T T T T T T T T T T T T T T T T T T T T T T T T T T T T T T T T T T T T T T T T T T T T . . . . . . . . . .    
859	. . . . . . . . . . . . . . . G G G . G G G G G G G G G G G G G G G G G G G G G G G G G G G G G G G G G G G G G G G G G G G G G G G G G . . . . . . . . . .    
860	. . . . . . . . . . . . . . . T T T . T T T T T T T T T T T T T T T T T T T T T T T T T T T T T T T T T T T T T T T T T T T T T T T T T . . . . . . . . . .    
861	. . . . . . . . . . . . . . . G G G . G G G G G G G G G G G G G G G G G G G G G G G G G G G G G G G G G G G G G G G G G G G G G G G G G . . . . . . . . . .    
862	. . . . . . . . . . . . . . . C C C . C C C C C C C C C C C C C C C C C C C C C C C C C C C C C C C C C C C C C C C C C C C C C C C C C . . . . . . . . . .    
863	. . . . . . . . . . . . . . . C C C . C C C C C C C C C C C C C C C C C C C C C C C C C C C C C C C C C C C C C C C C C C C C C C C C C . . . . . . . . . .    
864	. . . . . . . . . . . . . . . A A A . A A A A A A A A A A A A A A A A A A A A A A A A A A A A A A A A A A A A A A A A A A A A A A A A A . . . . . . . . . .    
865	. . . . . . . . . . . . . . . A A A . A A A A A A A A A A A A A A A A A A A A A A A A A A A A A A A A A A A A A A A A A A A A A A A A A . . . . . . . . . .    
866	. . . . . . . . . . . . . . . T T T . T T T T T T T T T T T T T T T T T T T T T T T T T T T T T T T T T T T T T T T T T T T T T T T T T . . . . . . . . . .    
867	. . . . . . . . . . . . . . . G G G . G G G G G G G G G G G G G G G G G G G G G G G G G G G G G G G G G G G G G G G G G G G G G G G G G . . . . . . . . . .    
868	. . . . . . . . . . . . . . . A A A . A A A A A A A A A A A A A A A A A A A A A A A A A A A A A A A A A A A A A A A A A A A A A A A A A . . . . . . . . . .    
869	. . . . . . . . . . . . . . . . G G . G G G G G G G G G G G G G G G G G G G G G G G G G G G G G G G G G G G G G G G G G G G G G G G G G . . . . . . . . . .    
870	. . . . . . . . . . . . . . . . T T . T T T T T T T T T T T T T T T T T T T T T T T T T T T T T T T T T T T T T T T T T T T T T T T T T . . . . . . . . . .    
871	. . . . . . . . . . . . . . . . T T . T T T T T T T T T T T T T T T T T T T T T T T T T T T T T T T T T T T T T T T T T T T T T T T T T . . . . . . . . . .    
872	. . . . . . . . . . . . . . . . T T . T T T T T T T T T T T T T T T T T T T T T T T T T T T T T T T T T T T T T T T T T T T T T T T T T . . . . . . . . . .    
873	. . . . . . . . . . . . . . . . T T . T T T T T T T T T T T T T T T T T T T T T T T T T T T T T T T T T T T T T T T T T T T T T T T T T T . . . . . . . . .    
874	. . . . . . . . . . . . . . . . C C . C C C C C C C C C C C C C C C C C C C C C C C C C C C C C C C C C C C C C C C C C C C C C C C C C C . . . . . . . . .    
875	. . . . . . . . . . . . . . . . . . . T T T T T T T T T T T T T T T T T T T T T T T T T T T T T T T T T T T T T T T T T T T T T T T T T T . . . . . . . . .    
876	. . . . . . . . . . . . . . . . . . . A A A A A A A A A A A A A A A A A A A A A A A A A A A A A A A A A A A A A A A A A A A A A A A A A A . . . . . . . . .    
877	. . . . . . . . . . . . . . . . . . . T T T T T T T T T T T T T T T T T T T T T T T T T T T T T T T T T T T T T T T T T T T T T T T T T T . . . . . . . . .    
878	. . . . . . . . . . . . . . . . . . . T T T T T T T T T T T T T T T T T T T T T T T T T T T T T T T T T T T T T T T T T T T T T T T T T T . . . . . . . . .    
879	. . . . . . . . . . . . . . . . . . . T T T T T T T T T T T T T T T T T T T T T T T T T T T T T T T T T T T T T T T T T T T T T T T T T T . . . . . . . . .    
880	. . . . . . . . . . . . . . . . . . . A A A A A A A A A A A A A A A A A A A A A A A A A A A A A A A A A A A A A A A A A A A A A A A A A A . . . . . . . . .    
881	. . . . . . . . . . . . . . . . . . . C C C C C C C C C C C C C C C C C C C C C C C C C C C C C C C C C C C C C C C C C C C C C C C C C C . . . . . . . . .    
882	. . . . . . . . . . . . . . . . . . . A A A A A A A A A A A A A A A A A A A A A A A A A A A A A A A A A A A A A A A A A A A A A A A A A A . . . . . . . . .    
883	. . . . . . . . . . . . . . . . . . . T T T T T T T T T T T T T T T T T T T T T T T T T T T T T T T T T T T T T T T T T T T T T T T T T T . . . . . . . . .    
884	. . . . . . . . . . . . . . . . . . . A A A A A A A A A A A A A A A A A A A A A A A A A A A A A A A A A A A A A A A A A A A A A A A A A A . . . . . . . . .    
885	. . . . . . . . . . . . . . . . . . . G G G G A G G G G G G G G G G G G G G G G G G G G G G G G G G G G G G G G G G G G G G G G G G G G G . . . . . . . . .    
886	. . . . . . . . . . . . . . . . . . . C C C C C C C C C C C C C C C C C C C C C C C C C C C C C C C C C C C C C C C C C C C C C C C C C C . . . . . . . . .    
887	. . . . . . . . . . . . . . . . . . . T T T T T T T T T T T T T T T T T T T T T T T T T T T T T T T T T T T T T T T T T T T T T T T T T T . . . . . . . . .    
888	. . . . . . . . . . . . . . . . . . . G G G G G G G G G G G G G G G G G G G G G G G G G G G G G G G G G G G G G G G G G G G G G G G G G G . . . . . . . . .    
889	. . . . . . . . . . . . . . . . . . . A A A A A A A A A A A A A A A A A A A A A A A A A A A A A A A A A A A A A A A A A A A A A A A A A A . . . . . . . . .    
890	. . . . . . . . . . . . . . . . . . . G G G G G G G G G G G G G G G G G G G G G G G G G G G G G G G G G G G G G G G G G G G G G G G G G G G . . . . . . . .    
891	. . . . . . . . . . . . . . . . . . . T T T T T T T T T T T T T T T T T T T T T T T T T T T T T T T T T T T T T T T T T T T T T T T T T T T . . . . . . . .    
892	. . . . . . . . . . . . . . . . . . . G G G G G G G G G G G G G G G G G G G G G G G G G G G G G G G G G G G G G G G G G G G G G G G G G G G . . . . . . . .    
893	. . . . . . . . . . . . . . . . . . . A A A A A A A A A A A A A A A A A A A A A A A A A A A A A A A A A A A A A A A A A A A A A A A A A A A . . . . . . . .    
894	. . . . . . . . . . . . . . . . . . . C C C C C C C C C C C C C C C C C C C C C C C C C C C C C C C C C C C C C C C C C C C C C C C C C C C . . . . . . . .    
895	. . . . . . . . . . . . . . . . . . . A A A A A A A A A A A A A A A A A A A A A A A A A A A A A A A A A A A A A A A A A A A A A A A A A A A . . . . . . . .    
896	. . . . . . . . . . . . . . . . . . . A A A A A A A A A A A A A A A A A A A A A A A A A A A A A A A A A A A A A A A A A A A A A A A A A A A . . . . . . . .    
897	. . . . . . . . . . . . . . . . . . . A A A A A A A A A A A A A A A A A A A A A A A A A A A A A A A A A A A A A A A A A A A A A A A A A A A . . . . . . . .    
898	. . . . . . . . . . . . . . . . . . . A A A A A A A A A A A A A A A A A A A A A A A A A A A A A A A A A A A A A A A A A A A A A A A A A A A . . . . . . . .    
899	. . . . . . . . . . . . . . . . . . . T T T T T T T T T T T T T T T T T T T T T T T T T T T T T T T T T T T T T T T T T T T T T T T T T T T . . . . . . . .    
900	. . . . . . . . . . . . . . . . . . . G G G G G G G G G G G G G G G G G G G G G G G G G G G G G G G G G G G G G G G G G G G G G G G G G G G . . . . . . . .    
901	. . . . . . . . . . . . . . . . . . . T T T T T T T T T T T T T T T T T T T T T T T T T T T T T T T T T T T T T T T T T T T T T T T T T T T . . . . . . . .    
902	. . . . . . . . . . . . . . . . . . . G G G G G G G G G G G G G G G G G G G G G G G G G G G G G G G G G G G G G G G G G G G - G G G G G G G . . . . . . . .    
903	. . . . . . . . . . . . . . . . . . . T T T T T T T T T T T T T T T T T T T T T T T T T T T T T T T T T T T T T T T T T T T T T T T T T T T . . . . . . . .    
904	. . . . . . . . . . . . . . . . . . . A A A A A A A A A A A A A A A A A A A A A A A A A A A A A A A A A A A A A A A A A A A A A A A A A A A . . . . . . . .    
905	. . . . . . . . . . . . . . . . . . . T T T T T T T T T T T T T T T T T T T T T T T T T T T T T T T T T T T T T T T T T T T T T C T T T T T . . . . . . . .    
906	. . . . . . . . . . . . . . . . . . . T T T T T T T T T T T T T T T T T T T T T T T T T T T T T T T T T T T T T T T T T T T T T T T T T T T . . . . . . . .    
907	. . . . . . . . . . . . . . . . . . . A A A A A A A A A A A A A A A A A A A A A A A A A A A A A A A A A A A A A A A A A A A A A A A A A A A . . . . . . . .    
908	. . . . . . . . . . . . . . . . . . . A A A A A A A A A A A A A A A A A A A A A A A A A A A A A A A A A A A A A A A A A A A A A A A A A A A . . . . . . . .    
909	. . . . . . . . . . . . . . . . . . . A A A A A A A A A A A A A A A A A A A A A A A A A A A A A A A A A A A A A A A A A A A A A A A A A A A . . . . . . . .    
910	. . . . . . . . . . . . . . . . . . . T T T T T T T T T T T T T T T T T T T T T T T T T T T T T T T T T T T T T T T T T T T T T T T T T T T . . . . . . . .    
911	. . . . . . . . . . . . . . . . . . . C C C C C C C C C C C C C C C C C C C C C C C C C C C C C C C C C C C C C C C C C C C C C C C C C C C . . . . . . . .    
912	. . . . . . . . . . . . . . . . . . . A A A A A A A A A A A A A A A A A A A A A A A A A A A A A A A A A A A A A A A A A A A A A A A A A A A . . . . . . . .    
913	. . . . . . . . . . . . . . . . . . . G G G G G G G G G G G G G G G G G G G G G G G G G G G G G G G G G G G G G G G G G G G G G G G G G G G . . . . . . . .    
914	. . . . . . . . . . . . . . . . . . . T T T T T T T T T T T T T T T T T T T T T T T T T T T T T T T T T T T T T T T T T T T T T T T T T T T . . . . . . . .    
915	. . . . . . . . . . . . . . . . . . . T T T T T T T T T T T T T T T T T T T T T T T T T T T T T T T T T T T T T T T T T T T T T T T T T T T . . . . . . . .    
916	. . . . . . . . . . . . . . . . . . . A A A A A A A A A A A A A A A A A A A A A A A A A A A A A A A A A A A A A A A A A A A A A A A A A A A . . . . . . . .    
917	. . . . . . . . . . . . . . . . . . . A A A A A A A A A A A A A A A A A A A A A A A A A A A A A A A A A A A A A A A A A A A A A A A A A A A . . . . . . . .    
918	. . . . . . . . . . . . . . . . . . . T T T T T T T T T T T T T T T T T T T T T T T T T T T T T T T T T T T T T T T T T T T T T T T T T T T . . . . . . . .    
919	. . . . . . . . . . . . . . . . . . . A A A A A A A A A A A A A A A A A A A A A A A A A A A A A A A A A A A A A A A A A A A A A A A A A A A . . . . . . . .    
920	. . . . . . . . . . . . . . . . . . . T T T T T T T T T T T T T T T T T T T T T T T T T T T T T T T T T T T T T T T T T T T T T T T T T T T . . . . . . . .    
921	. . . . . . . . . . . . . . . . . . . T T T T T T T T T T T T T T T T T T T T T T T T T T T T T T T T T T T T T T T T T T T T T T T T T T T . . . . . . . .    
922	. . . . . . . . . . . . . . . . . . . G G G G G G G G G G G G G G G G G G G G G G G G G G G G G G G G G G G G G G G G G G G G G G G G G G G . . . . . . . .    
923	. . . . . . . . . . . . . . . . . . . T T T T T T T T T T T T T T T T T T T T T T T T T T T T T T T T T T T T T T T T T T T T T T T T T T T . . . . . . . .    
924	. . . . . . . . . . . . . . . . . . . G G G G G G G G G G G G G G G G G G G G G G G G G G G G G G G G G G G G G G G G G G G G G G G G G G G . . . . . . . .    
925	. . . . . . . . . . . . . . . . . . . G G G G G G G G G G G G G G G G G G G G G G G G G G G G G G G G G G G G G G G G G G G G G G G G G G G . . . . . . . .    
926	. . . . . . . . . . . . . . . . . . . A A A A A A A A A A A A A A A A A A A A A A A A A A A A A A A A A A A A A A A A A A A A A A A A A A A . . . . . . . .    
927	. . . . . . . . . . . . . . . . . . . G G G G G G G G G G G G G G G G G G G G G G G G G G G G G G G G G G G G G G G G G G G G G G G G G G G . . . . . . . .    
928	. . . . . . . . . . . . . . . . . . . A A A A A A A A A A A A A A A A A A A A A A A A A A A A A A A A A A A A A A A A A A A A A A A A A A A . . . . . . . .    
929	. . . . . . . . . . . . . . . . . . . A A A A A A A A A A A A A A A A A A A A A A A A A A A A A A A A A A A A A A A A A A A A A A A A A A A . . . . . . . .    
930	. . . . . . . . . . . . . . . . . . . C C C C C C C C C C C C C C C C C C C C C C C C C C C C C C C C C C C C C C C C C C C C C C C C C C C . . . . . . . .    
931	. . . . . . . . . . . . . . . . . . . T T T T T T T T T T T T T T T T T T T T T T T T T T T T T T T T T T T T T T T T T T T T T T T T T T T . . . . . . . .    
932	. . . . . . . . . . . . . . . . . . . C C C C C C C C C N C C C C C C C C C C C C C C C C C C C C C C C C C C C C C C C C C C C C C C C C C . . . . . . . .    
933	. . . . . . . . . . . . . . . . . . . G G G G G G G G G G G G G G G G G G G G G G G G G G G G G G G G G G G G G G G G G G G G G G G G G G G . . . . . . . .    
934	. . . . . . . . . . . . . . . . . . . A A A A A A A A A A A A A A A A A A A A A A A A A A A A A A A A A A A A A A A A A A A A A A A A A A A . . . . . . . .    
935	. . . . . . . . . . . . . . . . . . . T T T T T T T T T T T T T T T T T T T T T T T T T T T T T T T T T T T T T T T T T T T T T T T T T T T . . . . . . . .    
936	. . . . . . . . . . . . . . . . . . . G G G A G G G G G G G G G G G G G G G G G G G G G G G G G G G G G G G G G G G G G G G G G G G G G G G . . . . . . . .    
937	. . . . . . . . . . . . . . . . . . . T T T . T T T T T T T T T T T T T T T T T T T T T T T T T T T T T T T T T T T T T T T T T T T T T T T . . . . . . . .    
938	. . . . . . . . . . . . . . . . . . . G G G . G G G G G G G G G G G G G G G G G G G G G G G G G G G G G G G G G G G G G G G G G G G G G G G . . . . . . . .    
939	. . . . . . . . . . . . . . . . . . . A A A . A A A A A A A A A A A A A A A A A A A A A A A A A A A A A A A A A A A A A A A A A A A A A A A . . . . . . . .    
940	. . . . . . . . . . . . . . . . . . . T T T . T T T T T T T T T T T T T T T T T T T T T T T T T T T T T T T T T T T T T T T T T T T T T T T . . . . . . . .    
941	. . . . . . . . . . . . . . . . . . . A A A . A A A A A A A A A A A A A A A A A A A A A A A A A A A A A A A A A A A A A A A A A A A A A A A . . . . . . . .    
942	. . . . . . . . . . . . . . . . . . . A A A . A A A A A A A A A A A A A A A A A A A A A A A A A A A A A A A A A A A A A A A A A A A A A A A . . . . . . . .    
943	. . . . . . . . . . . . . . . . . . . T T T . T T T T T T T T T T T T T T T T T T T T T T T T T T T T T T T T T T T T T T T T T T T T T T T . . . . . . . .    
944	. . . . . . . . . . . . . . . . . . . G G G . G G G G G G G G G G G G G G G G G G G G G G G G G G G G G G G G G G G G G G G G G G G G G G G . . . . . . . .    
945	. . . . . . . . . . . . . . . . . . . T T T . T T T T T T T T T T T T T T T T T T T T T T T T T T T T T T T T T T T T T T T T T T T T T T T . . . . . . . .    
946	. . . . . . . . . . . . . . . . . . . A A A . A A A A A A A A A A A A A A A A A A A A A A A A A A A A A A A A A A A A A A A A A A A A A A A . . . . . . . .    
947	. . . . . . . . . . . . . . . . . . . T T T . T T T T T T T T T T T T T T T T T T T T T T T T T T T T T T T T T T T T T T T T T T T T T T T . . . . . . . .    
948	. . . . . . . . . . . . . . . . . . . T T T . T T T T T T T T T T T T T T T T T T T T T T T T T T T T T T T T T T T T T T T T T T T T T T T . . . . . . . .    
949	. . . . . . . . . . . . . . . . . . . T T T . T T T T T T T T T T T T T T T T T T T T T T T T T T T T T T T T T T T T T T T T T T T T T T T . . . . . . . .    
950	. . . . . . . . . . . . . . . . . . . C A C . A A A A A A A A A A A A A A A A A A A A A A A A A A A A A A A A A A A A A A A A A A A A A A A . . . . . . . .    
951	. . . . . . . . . . . . . . . . . . . . C . . C C C C C C C C C C C C C C C C C C C C C C C C C C C C C C C C C C C C C C C C C C C C C C C . . . . . . . .    
952	. . . . . . . . . . . . . . . . . . . . A . . A A A A A A A A A A A A A A A A A A A A A A A A A A A A A A A A A A A A A A A A A A A A A A A . . . . . . . .    
953	. . . . . . . . . . . . . . . . . . . . C . . A A A A A A A A A A A A A A A A A A A A A A A A A A A A A A A A A A A A A A A A A A A A A A A . . . . . . . .    
954	. . . . . . . . . . . . . . . . . . . . . . . T T T T T T T T T T T T T T T T T T T T T T T T T T T T T T T T T T T T T T T T T T T T T T T . . . . . . . .    
955	. . . . . . . . . . . . . . . . . . . . . . . A A A A A A A A A A A A A A A A A A A A A A A A A A A A A A A A A A A A A A A A A A A A A A A . . . . . . . .    
956	. . . . . . . . . . . . . . . . . . . . . . . A A A A A A A A A A A A A A A A A A A A A A A A A A A A A A A A A A A A A A A A A A A A A A A . . . . . . . .    
957	. . . . . . . . . . . . . . . . . . . . . . . A A A A A A A A A A A A A A A A A A A A A A A A A A A A A A A A A A A A A A A A A A A A A A A . . . . . . . .    
958	. . . . . . . . . . . . . . . . . . . . . . . C C C C C C C C C C C C C C C C C C C C C C C C C C C C C C C C C C C C C C C C C C C C C C C . . . . . . . .    
959	. . . . . . . . . . . . . . . . . . . . . . . A A A A A A A A A A A A A A A A A A A A A A A A A A A A A A A A A A A A A A A A A A A A A A A . . . . . . . .    
960	. . . . . . . . . . . . . . . . . . . . . . . A A A A A A A A A A A A A A A A A A A A A A A A A A A A A A A A A A A A A A A A A A A A A A A . . . . . . . .    
961	. . . . . . . . . . . . . . . . . . . . . . . A A A A A A A A A A A A A A A A A A A A A A A A A A A A A A A A A A A A A A A A A A A A A A A . . . . . . . .    
962	. . . . . . . . . . . . . . . . . . . . . . . A A A A A A A A A A A A A A A A A A A A A A A A A A A A A A A A A A A A A A A A A A A A A A A . . . . . . . .    
963	. . . . . . . . . . . . . . . . . . . . . . . T T T T T T T T T T T T T T T T T T T T T T T T T T T T T T T T T T T T T T T T T T T T T T T . . . . . . . .    
964	. . . . . . . . . . . . . . . . . . . . . . . A A A A A A A A A A A A A A A A A A A A A A A A A A A A A A A A A A A A A A A A A A A A A A A . . . . . . . .    
965	. . . . . . . . . . . . . . . . . . . . . . . A A A A A A A A A A A A A A A A A A A A A A A A A A A A A A A A A A A A A A A A A A A A A A A . . . . . . . .    
966	. . . . . . . . . . . . . . . . . . . . . . . A A A A A A A A A A A A A A A A A A A A A A A A A A A A A A A A A A A A A A A A A A A A A A A . . . . . . . .    
967	. . . . . . . . . . . . . . . . . . . . . . . T T T T T T T T T T T T T T T T T T T T T T T T T T T T T T T T T T T T T T T T T T T T T T T . . . . . . . .    
968	. . . . . . . . . . . . . . . . . . . . . . . G G G G G G G G G G G G G G G G G G G G G G G G G G G G G G G G G G G G G G G G G G G G G G G . . . . . . . .    
969	. . . . . . . . . . . . . . . . . . . . . . . C C C C C C C C C C C C C C C C C C C C C C C C C C C C C C C C C C C C C C C C C C C C C C C C . . . . . . .    
970	. . . . . . . . . . . . . . . . . . . . . . . A A A A A A A A A A A A A A A A A A A A A A A A A A A A A A A A A A A A A A A A A A A A A A A A . . . . . . .    
971	. . . . . . . . . . . . . . . . . . . . . . . C C C C C C C C C C C C C C C C C C C C C C C C C C C C C C C C C C C C C C C C C C C C C C C C . . . . . . .    
972	. . . . . . . . . . . . . . . . . . . . . . . T T T T T T T T T T T T T T T T T T T T T T T T T T T T T T T T T T T T T T T T T T T T T T T T . . . . . . .    
973	. . . . . . . . . . . . . . . . . . . . . . . T T T T T T T T T T T T T T T T T T T T T T T T T T T T T T T T T T T T T T T T T T T T T T T T . . . . . . .    
974	. . . . . . . . . . . . . . . . . . . . . . . G G G G G G G G G G G G G G G G G G G G G G G G G G G G G G G G G G G G G G G G G G G G G G G G . . . . . . .    
975	. . . . . . . . . . . . . . . . . . . . . . . A A A A A A A A A A A A A A A A A A A A A A A A A A A A A A A A . A A A A A A A A A A A A A A A . . . . . . .    
976	. . . . . . . . . . . . . . . . . . . . . . . T T T T T T T T T T T T T T T T T T T T T T T T T T T T T T T T . T T T T T T T T T T T T T T T . . . . . . .    
977	. . . . . . . . . . . . . . . . . . . . . . . G G G G G G G G G G G G G G G G G G G G G G G G G G G G G G G G . G G G G G G G G G G G G G G G . . . . . . .    
978	. . . . . . . . . . . . . . . . . . . . . . . A A A A A A A A A A A A A A A A A A A A A A A A A A A A A A A A . A A A A A A A A A A A A A A A . . . . . . .    
979	. . . . . . . . . . . . . . . . . . . . . . . C C C C C C C C C C C C C C C C C C C C C C C C C C C C C C C C . C C C C C C C C C C C C C C C . . . . . . .    
980	. . . . . . . . . . . . . . . . . . . . . . . C C C C C C C C C C C C C C C C C C C C C C C C C C C C C C C C . C C C C C C C C C C C C C C C . . . . . . .    
981	. . . . . . . . . . . . . . . . . . . . . . . C T . T T T T T T T T T T T T T T T T T T T T T T . T T T T C T . T . . T T T T T T T T T T T T . . . . . . .    
982	. . . . . . . . . . . . . . . . . . . . . . . . . . . A . . . . . . . . . . . . . . . . . . . . . . . . . . . . . . . . . . . . . A A A A A A . . . . . . .    
983	. . . . . . . . . . . . . . . . . . . . . . . . . . . A . . . . . . . . . . . . . . . . . . . . . . . . . . . . . . . . . . . . . A A A A A A . . . . . . .    
984	. . . . . . . . . . . . . . . . . . . . . . . . . . . A . . . . . . . . . . . . . . . . . . . . . . . . . . . . . . . . . . . . . A A A A A A . . . . . . .    
985	. . . . . . . . . . . . . . . . . . . . . . . . . . . A . . . . . . . . . . . . . . . . . . . . . . . . . . . . . . . . . . . . . A A A A A A . . . . . . .    
986	. . . . . . . . . . . . . . . . . . . . . . . . . . . C . . . . . . . . . . . . . . . . . . . . . . . . . . . . . . . . . . . . . C C C C C C . . . . . . .    
987	. . . . . . . . . . . . . . . . . . . . . . . . . . . . . . . . . . . . . . . . . . . . . . . . . . . . . . . . . . . . . . . . . A A A A A A . . . . . . .    
988	. . . . . . . . . . . . . . . . . . . . . . . . . . . . . . . . . . . . . . . . . . . . . . . . . . . . . . . . . . . . . . . . . T T T T T T . . . . . . .    
989	. . . . . . . . . . . . . . . . . . . . . . . . . . . . . . . . . . . . . . . . . . . . . . . . . . . . . . . . . . . . . . . . . G G G G G G . . . . . . .    
990	. . . . . . . . . . . . . . . . . . . . . . . . . . . . . . . . . . . . . . . . . . . . . . . . . . . . . . . . . . . . . . . . . C C C C C C . . . . . . .    
991	. . . . . . . . . . . . . . . . . . . . . . . . . . . . . . . . . . . . . . . . . . . . . . . . . . . . . . . . . . . . . . . . . A A A A A A A . . . . . .    
992	. . . . . . . . . . . . . . . . . . . . . . . . . . . . . . . . . . . . . . . . . . . . . . . . . . . . . . . . . . . . . . . . . A A A A A A A . . . . . .    
993	. . . . . . . . . . . . . . . . . . . . . . . . . . . . . . . . . . . . . . . . . . . . . . . . . . . . . . . . . . . . . . . . . T T T T T T T . . . . . .    
994	. . . . . . . . . . . . . . . . . . . . . . . . . . . . . . . . . . . . . . . . . . . . . . . . . . . . . . . . . . . . . . . . . T T T T T T T . . . . . .    
995	. . . . . . . . . . . . . . . . . . . . . . . . . . . . . . . . . . . . . . . . . . . . . . . . . . . . . . . . . . . . . . . . . C C C C C C C . . . . . .    
996	. . . . . . . . . . . . . . . . . . . . . . . . . . . . . . . . . . . . . . . . . . . . . . . . . . . . . . . . . . . . . . . . . T T T T T T T . . . . . .    
997	. . . . . . . . . . . . . . . . . . . . . . . . . . . . . . . . . . . . . . . . . . . . . . . . . . . . . . . . . . . . . . . . . A A A A A A A . . . . . .    
998	. . . . . . . . . . . . . . . . . . . . . . . . . . . . . . . . . . . . . . . . . . . . . . . . . . . . . . . . . . . . . . . . . C C C C C C C . . . . . .    
999	. . . . . . . . . . . . . . . . . . . . . . . . . . . . . . . . . . . . . . . . . . . . . . . . . . . . . . . . . . . . . . . . . T T T T T T T . . . . . .    
1000	. . . . . . . . . . . . . . . . . . . . . . . . . . . . . . . . . . . . . . . . . . . . . . . . . . . . . . . . . . . . . . . . . T T T T T T T . . . . . .    
1001	. . . . . . . . . . . . . . . . . . . . . . . . . . . . . . . . . . . . . . . . . . . . . . . . . . . . . . . . . . . . . . . . . G G G G G G G . . . . . .    
1002	. . . . . . . . . . . . . . . . . . . . . . . . . . . . . . . . . . . . . . . . . . . . . . . . . . . . . . . . . . . . . . . . . A A A A A A A . . . . . .    
1003	. . . . . . . . . . . . . . . . . . . . . . . . . . . . . . . . . . . . . . . . . . . . . . . . . . . . . . . . . . . . . . . . . C C C C C C C . . . . . .    
1004	. . . . . . . . . . . . . . . . . . . . . . . . . . . . . . . . . . . . . . . . . . . . . . . . . . . . . . . . . . . . . . . . . T T T T T T T . . . . . .    
1005	. . . . . . . . . . . . . . . . . . . . . . . . . . . . . . . . . . . . . . . . . . . . . . . . . . . . . . . . . . . . . . . . . T T T T T T T . . . . . .    
1006	. . . . . . . . . . . . . . . . . . . . . . . . . . . . . . . . . . . . . . . . . . . . . . . . . . . . . . . . . . . . . . . . . T T T T T T T . . . . . .    
1007	. . . . . . . . . . . . . . . . . . . . . . . . . . . . . . . . . . . . . . . . . . . . . . . . . . . . . . . . . . . . . . . . . T T T T T T T . . . . . .    
1008	. . . . . . . . . . . . . . . . . . . . . . . . . . . . . . . . . . . . . . . . . . . . . . . . . . . . . . . . . . . . . . . . . A A A A A A A . . . . . .    
1009	. . . . . . . . . . . . . . . . . . . . . . . . . . . . . . . . . . . . . . . . . . . . . . . . . . . . . . . . . . . . . . . . . T T T T T T T . . . . . .    
1010	. . . . . . . . . . . . . . . . . . . . . . . . . . . . . . . . . . . . . . . . . . . . . . . . . . . . . . . . . . . . . . . . . T T T T T T T . . . . . .    
1011	. . . . . . . . . . . . . . . . . . . . . . . . . . . . . . . . . . . . . . . . . . . . . . . . . . . . . . . . . . . . . . . . . G G G G G G G . . . . . .    
1012	. . . . . . . . . . . . . . . . . . . . . . . . . . . . . . . . . . . . . . . . . . . . . . . . . . . . . . . . . . . . . . . . . A A A A A A A . . . . . .    
1013	. . . . . . . . . . . . . . . . . . . . . . . . . . . . . . . . . . . . . . . . . . . . . . . . . . . . . . . . . . . . . . . . . G G G G G G G . . . . . .    
1014	. . . . . . . . . . . . . . . . . . . . . . . . . . . . . . . . . . . . . . . . . . . . . . . . . . . . . . . . . . . . . . . . . T T T T T T T . . . . . .    
1015	. . . . . . . . . . . . . . . . . . . . . . . . . . . . . . . . . . . . . . . . . . . . . . . . . . . . . . . . . . . . . . . . . A A A A A A A . . . . . .    
1016	. . . . . . . . . . . . . . . . . . . . . . . . . . . . . . . . . . . . . . . . . . . . . . . . . . . . . . . . . . . . . . . . . A A A A A A A . . . . . .    
1017	. . . . . . . . . . . . . . . . . . . . . . . . . . . . . . . . . . . . . . . . . . . . . . . . . . . . . . . . . . . . . . . . . T T T T T T T . . . . . .    
1018	. . . . . . . . . . . . . . . . . . . . . . . . . . . . . . . . . . . . . . . . . . . . . . . . . . . . . . . . . . . . . . . . . G G G G G G G . . . . . .    
1019	. . . . . . . . . . . . . . . . . . . . . . . . . . . . . . . . . . . . . . . . . . . . . . . . . . . . . . . . . . . . . . . . . G G G G G G G . . . . . .    
1020	. . . . . . . . . . . . . . . . . . . . . . . . . . . . . . . . . . . . . . . . . . . . . . . . . . . . . . . . . . . . . . . . . T T T T T T T . . . . . .    
1021	. . . . . . . . . . . . . . . . . . . . . . . . . . . . . . . . . . . . . . . . . . . . . . . . . . . . . . . . . . . . . . . . . G G G G G G G . . . . . .    
1022	. . . . . . . . . . . . . . . . . . . . . . . . . . . . . . . . . . . . . . . . . . . . . . . . . . . . . . . . . . . . . . . . . C C C C C C C . . . . . .    
1023	. . . . . . . . . . . . . . . . . . . . . . . . . . . . . . . . . . . . . . . . . . . . . . . . . . . . . . . . . . . . . . . . . A A A A A A A . . . . . .    
1024	. . . . . . . . . . . . . . . . . . . . . . . . . . . . . . . . . . . . . . . . . . . . . . . . . . . . . . . . . . . . . . . . . A A A A A A A . . . . . .    
1025	. . . . . . . . . . . . . . . . . . . . . . . . . . . . . . . . . . . . . . . . . . . . . . . . . . . . . . . . . . . . . . . . . C C C C C C C . . . . . .    
1026	. . . . . . . . . . . . . . . . . . . . . . . . . . . . . . . . . . . . . . . . . . . . . . . . . . . . . . . . . . . . . . . . . A A A A A A A . . . . . .    
1027	. . . . . . . . . . . . . . . . . . . . . . . . . . . . . . . . . . . . . . . . . . . . . . . . . . . . . . . . . . . . . . . . . A A A A A A A . . . . . .    
1028	. . . . . . . . . . . . . . . . . . . . . . . . . . . . . . . . . . . . . . . . . . . . . . . . . . . . . . . . . . . . . . . . . C C C C C C C . . . . . .    
1029	. . . . . . . . . . . . . . . . . . . . . . . . . . . . . . . . . . . . . . . . . . . . . . . . . . . . . . . . . . . . . . . . . G G G G G G G . . . . . .    
1030	. . . . . . . . . . . . . . . . . . . . . . . . . . . . . . . . . . . . . . . . . . . . . . . . . . . . . . . . . . . . . . . . . C C C C C C C . . . . . .    
1031	. . . . . . . . . . . . . . . . . . . . . . . . . . . . . . . . . . . . . . . . . . . . . . . . . . . . . . . . . . . . . . . . . A A A A A A A . . . . . .    
1032	. . . . . . . . . . . . . . . . . . . . . . . . . . . . . . . . . . . . . . . . . . . . . . . . . . . . . . . . . . . . . . . . . G G G G G G G . . . . . .    
1033	. . . . . . . . . . . . . . . . . . . . . . . . . . . . . . . . . . . . . . . . . . . . . . . . . . . . . . . . . . . . . . . . . G G G G G G G . . . . . .    
1034	. . . . . . . . . . . . . . . . . . . . . . . . . . . . . . . . . . . . . . . . . . . . . . . . . . . . . . . . . . . . . . . . . T T T T T T T . . . . . .    
1035	. . . . . . . . . . . . . . . . . . . . . . . . . . . . . . . . . . . . . . . . . . . . . . . . . . . . . . . . . . . . . . . . . G G G G G G G . . . . . .    
1036	. . . . . . . . . . . . . . . . . . . . . . . . . . . . . . . . . . . . . . . . . . . . . . . . . . . . . . . . . . . . . . . . . C C C C C C C . . . . . .    
1037	. . . . . . . . . . . . . . . . . . . . . . . . . . . . . . . . . . . . . . . . . . . . . . . . . . . . . . . . . . . . . . . . . C C C C C C C C . . . . .    
1038	. . . . . . . . . . . . . . . . . . . . . . . . . . . . . . . . . . . . . . . . . . . . . . . . . . . . . . . . . . . . . . . . . C C C C C C C C . . . . .    
1039	. . . . . . . . . . . . . . . . . . . . . . . . . . . . . . . . . . . . . . . . . . . . . . . . . . . . . . . . . . . . . . . . . T T T T T T T T . . . . .    
1040	. . . . . . . . . . . . . . . . . . . . . . . . . . . . . . . . . . . . . . . . . . . . . . . . . . . . . . . . . . . . . . . . . G G G G G G G G . . . . .    
1041	. . . . . . . . . . . . . . . . . . . . . . . . . . . . . . . . . . . . . . . . . . . . . . . . . . . . . . . . . . . . . . . . . G G G G G G G G . G . . .    
1042	. . . . . . . . . . . . . . . . . . . . . . . . . . . . . . . . . . . . . . . . . . . . . . . . . . . . . . . . . . . . . . . . . C C C C C C C C . C . . .    
1043	. . . . . . . . . . . . . . . . . . . . . . . . . . . . . . . . . . . . . . . . . . . . . . . . . . . . . . . . . . . . . . . . . C C C C C C C C . C . . .    
1044	. . . . . . . . . . . . . . . . . . . . . . . . . . . . . . . . . . . . . . . . . . . . . . . . . . . . . . . . . . . . . . . . . C C C C C C C C . C . . .    
1045	. . . . . . . . . . . . . . . . . . . . . . . . . . . . . . . . . . . . . . . . . . . . . . . . . . . . . . . . . . . . . . . . . T T T T T T T T . T . . .    
1046	. . . . . . . . . . . . . . . . . . . . . . . . . . . . . . . . . . . . . . . . . . . . . . . . . . . . . . . . . . . . . . . . . T T T T T T T T . T . . .    
1047	. . . . . . . . . . . . . . . . . . . . . . . . . . . . . . . . . . . . . . . . . . . . . . . . . . . . . . . . . . . . . . . . . T T T T T T T T . T . . .    
1048	. . . . . . . . . . . . . . . . . . . . . . . . . . . . . . . . . . . . . . . . . . . . . . . . . . . . . . . . . . . . . . . . . A A A A A A A A . A . . .    
1049	. . . . . . . . . . . . . . . . . . . . . . . . . . . . . . . . . . . . . . . . . . . . . . . . . . . . . . . . . . . . . . . . . A A A A A A A A . A . . .    
1050	. . . . . . . . . . . . . . . . . . . . . . . . . . . . . . . . . . . . . . . . . . . . . . . . . . . . . . . . . . . . . . . . . T T T T T T T T . T . . .    
1051	. . . . . . . . . . . . . . . . . . . . . . . . . . . . . . . . . . . . . . . . . . . . . . . . . . . . . . . . . . . . . . . . . A A A A A A A A . A . . .    
1052	. . . . . . . . . . . . . . . . . . . . . . . . . . . . . . . . . . . . . . . . . . . . . . . . . . . . . . . . . . . . . . . . . C C C C C C C C . C . . .    
1053	. . . . . . . . . . . . . . . . . . . . . . . . . . . . . . . . . . . . . . . . . . . . . . . . . . . . . . . . . . . . . . . . . C C C C C C C C . C . . .    
1054	. . . . . . . . . . . . . . . . . . . . . . . . . . . . . . . . . . . . . . . . . . . . . . . . . . . . . . . . . . . . . . . . . A A A A A A A A . A . . .    
1055	. . . . . . . . . . . . . . . . . . . . . . . . . . . . . . . . . . . . . . . . . . . . . . . . . . . . . . . . . . . . . . . . . A A A A A A A A . A . . .    
1056	. . . . . . . . . . . . . . . . . . . . . . . . . . . . . . . . . . . . . . . . . . . . . . . . . . . . . . . . . . . . . . . . . T T T T T T T T . T . . .    
1057	. . . . . . . . . . . . . . . . . . . . . . . . . . . . . . . . . . . . . . . . . . . . . . . . . . . . . . . . . . . . . . . . . C C C C C C C C . C . . .    
1058	. . . . . . . . . . . . . . . . . . . . . . . . . . . . . . . . . . . . . . . . . . . . . . . . . . . . . . . . . . . . . . . . . A A A A A A A A . A . . .    
1059	. . . . . . . . . . . . . . . . . . . . . . . . . . . . . . . . . . . . . . . . . . . . . . . . . . . . . . . . . . . . . . . . . T T T T T T T T . T . . .    
1060	. . . . . . . . . . . . . . . . . . . . . . . . . . . . . . . . . . . . . . . . . . . . . . . . . . . . . . . . . . . . . . . . . T T T T T T T T . T . . .    
1061	. . . . . . . . . . . . . . . . . . . . . . . . . . . . . . . . . . . . . . . . . . . . . . . . . . . . . . . . . . . . . . . . . G G G G G G G G G G . . .    
1062	. . . . . . . . . . . . . . . . . . . . . . . . . . . . . . . . . . . . . . . . . . . . . . . . . . . . . . . . . . . . . . . . . C C C C C C C C C C . . .    
1063	. . . . . . . . . . . . . . . . . . . . . . . . . . . . . . . . . . . . . . . . . . . . . . . . . . . . . . . . . . . . . . . . . A A A A A A A A A A . . .    
1064	. . . . . . . . . . . . . . . . . . . . . . . . . . . . . . . . . . . . . . . . . . . . . . . . . . . . . . . . . . . . . . . . . C C C C C C C C C C . . .    
1065	. . . . . . . . . . . . . . . . . . . . . . . . . . . . . . . . . . . . . . . . . . . . . . . . . . . . . . . . . . . . . . . . . C C C C C C C C C C . . .    
1066	. . . . . . . . . . . . . . . . . . . . . . . . . . . . . . . . . . . . . . . . . . . . . . . . . . . . . . . . . . . . . . . . . A A A A A A A A A A . . .    
1067	. . . . . . . . . . . . . . . . . . . . . . . . . . . . . . . . . . . . . . . . . . . . . . . . . . . . . . . . . . . . . . . . . C C C C C C C C C C . . .    
1068	. . . . . . . . . . . . . . . . . . . . . . . . . . . . . . . . . . . . . . . . . . . . . . . . . . . . . . . . . . . . . . . . . A A A A A A A A A A . . .    
1069	. . . . . . . . . . . . . . . . . . . . . . . . . . . . . . . . . . . . . . . . . . . . . . . . . . . . . . . . . . . . . . . . . T T T T T T T T T T . . .    
1070	. . . . . . . . . . . . . . . . . . . . . . . . . . . . . . . . . . . . . . . . . . . . . . . . . . . . . . . . . . . . . . . . . G G G G G G G G G G . . .    
1071	. . . . . . . . . . . . . . . . . . . . . . . . . . . . . . . . . . . . . . . . . . . . . . . . . . . . . . . . . . . . . . . . . G G G G G G G G G G . . .    
1072	. . . . . . . . . . . . . . . . . . . . . . . . . . . . . . . . . . . . . . . . . . . . . . . . . . . . . . . . . . . . . . . . . A A A A A A A A A A . . .    
1073	. . . . . . . . . . . . . . . . . . . . . . . . . . . . . . . . . . . . . . . . . . . . . . . . . . . . . . . . . . . . . . . . . G G G G G G G G G G . . .    
1074	. . . . . . . . . . . . . . . . . . . . . . . . . . . . . . . . . . . . . . . . . . . . . . . . . . . . . . . . . . . . . . . . . G G G G G G G G G G . . .    
1075	. . . . . . . . . . . . . . . . . . . . . . . . . . . . . . . . . . . . . . . . . . . . . . . . . . . . . . . . . . . . . . . . . . A A A A A A A A A . . .    
1076	. . . . . . . . . . . . . . . . . . . . . . . . . . . . . . . . . . . . . . . . . . . . . . . . . . . . . . . . . . . . . . . . . . G G G G G G G G G . . .    
1077	. . . . . . . . . . . . . . . . . . . . . . . . . . . . . . . . . . . . . . . . . . . . . . . . . . . . . . . . . . . . . . . . . . T T T T T T T T T . . .    
1078	. . . . . . . . . . . . . . . . . . . . . . . . . . . . . . . . . . . . . . . . . . . . . . . . . . . . . . . . . . . . . . . . . . C C C C C C C C C . . .    
1079	. . . . . . . . . . . . . . . . . . . . . . . . . . . . . . . . . . . . . . . . . . . . . . . . . . . . . . . . . . . . . . . . . . A A A A A A A A A . . .    
1080	. . . . . . . . . . . . . . . . . . . . . . . . . . . . . . . . . . . . . . . . . . . . . . . . . . . . . . . . . . . . . . . . . . G G G G G G G G G . . .    
1081	. . . . . . . . . . . . . . . . . . . . . . . . . . . . . . . . . . . . . . . . . . . . . . . . . . . . . . . . . . . . . . . . . . C C C C C C C C C . . .    
1082	. . . . . . . . . . . . . . . . . . . . . . . . . . . . . . . . . . . . . . . . . . . . . . . . . . . . . . . . . . . . . . . . . . G G G G G G G G G . . .    
1083	. . . . . . . . . . . . . . . . . . . . . . . . . . . . . . . . . . . . . . . . . . . . . . . . . . . . . . . . . . . . . . . . . . G G G G G G G G G . . .    
1084	. . . . . . . . . . . . . . . . . . . . . . . . . . . . . . . . . . . . . . . . . . . . . . . . . . . . . . . . . . . . . . . . . . C C C C C C C C C . . .    
1085	. . . . . . . . . . . . . . . . . . . . . . . . . . . . . . . . . . . . . . . . . . . . . . . . . . . . . . . . . . . . . . . . . . A A A A A A A A A . . .    
1086	. . . . . . . . . . . . . . . . . . . . . . . . . . . . . . . . . . . . . . . . . . . . . . . . . . . . . . . . . . . . . . . . . . T T T T T T T T T . . .    
1087	. . . . . . . . . . . . . . . . . . . . . . . . . . . . . . . . . . . . . . . . . . . . . . . . . . . . . . . . . . . . . . . . . . C C C C C C C C C . . .    
1088	. . . . . . . . . . . . . . . . . . . . . . . . . . . . . . . . . . . . . . . . . . . . . . . . . . . . . . . . . . . . . . . . . . C C C C C C C C C . . .    
1089	. . . . . . . . . . . . . . . . . . . . . . . . . . . . . . . . . . . . . . . . . . . . . . . . . . . . . . . . . . . . . . . . . . A A A A A A A A A . . .    
1090	. . . . . . . . . . . . . . . . . . . . . . . . . . . . . . . . . . . . . . . . . . . . . . . . . . . . . . . . . . . . . . . . . . C C C C C C C C C . . .    
1091	. . . . . . . . . . . . . . . . . . . . . . . . . . . . . . . . . . . . . . . . . . . . . . . . . . . . . . . . . . . . . . . . . . A A A A A A A A A . . .    
1092	. . . . . . . . . . . . . . . . . . . . . . . . . . . . . . . . . . . . . . . . . . . . . . . . . . . . . . . . . . . . . . . . . . C C C C C C C C C . . .    
1093	. . . . . . . . . . . . . . . . . . . . . . . . . . . . . . . . . . . . . . . . . . . . . . . . . . . . . . . . . . . . . . . . . . T T T T T T T T T . . .    
1094	. . . . . . . . . . . . . . . . . . . . . . . . . . . . . . . . . . . . . . . . . . . . . . . . . . . . . . . . . . . . . . . . . . C C C C C C C C C . . .    
1095	. . . . . . . . . . . . . . . . . . . . . . . . . . . . . . . . . . . . . . . . . . . . . . . . . . . . . . . . . . . . . . . . . . . C C C C C C C C . . .    
1096	. . . . . . . . . . . . . . . . . . . . . . . . . . . . . . . . . . . . . . . . . . . . . . . . . . . . . . . . . . . . . . . . . . . T T T T T T T T . . .    
1097	. . . . . . . . . . . . . . . . . . . . . . . . . . . . . . . . . . . . . . . . . . . . . . . . . . . . . . . . . . . . . . . . . . . T T T T T T T T . . .    
1098	. . . . . . . . . . . . . . . . . . . . . . . . . . . . . . . . . . . . . . . . . . . . . . . . . . . . . . . . . . . . . . . . . . . G G G G G G G G . . .    
1099	. . . . . . . . . . . . . . . . . . . . . . . . . . . . . . . . . . . . . . . . . . . . . . . . . . . . . . . . . . . . . . . . . . . G G G G G G G G . . .    
1100	. . . . . . . . . . . . . . . . . . . . . . . . . . . . . . . . . . . . . . . . . . . . . . . . . . . . . . . . . . . . . . . . . . . C C C C C C C C . . .    
1101	. . . . . . . . . . . . . . . . . . . . . . . . . . . . . . . . . . . . . . . . . . . . . . . . . . . . . . . . . . . . . . . . . . . C C C C C C C C . . .    
1102	. . . . . . . . . . . . . . . . . . . . . . . . . . . . . . . . . . . . . . . . . . . . . . . . . . . . . . . . . . . . . . . . . . . A A A A A A A A . . .    
1103	. . . . . . . . . . . . . . . . . . . . . . . . . . . . . . . . . . . . . . . . . . . . . . . . . . . . . . . . . . . . . . . . . . . C C C C C C C C . . .    
1104	. . . . . . . . . . . . . . . . . . . . . . . . . . . . . . . . . . . . . . . . . . . . . . . . . . . . . . . . . . . . . . . . . . . C C C C C C C C . . .    
1105	. . . . . . . . . . . . . . . . . . . . . . . . . . . . . . . . . . . . . . . . . . . . . . . . . . . . . . . . . . . . . . . . . . . A A A A A A A A . . .    
1106	. . . . . . . . . . . . . . . . . . . . . . . . . . . . . . . . . . . . . . . . . . . . . . . . . . . . . . . . . . . . . . . . . . . T T T T T T T T . . .    
1107	. . . . . . . . . . . . . . . . . . . . . . . . . . . . . . . . . . . . . . . . . . . . . . . . . . . . . . . . . . . . . . . . . . . G G G G G G G G . . .    
1108	. . . . . . . . . . . . . . . . . . . . . . . . . . . . . . . . . . . . . . . . . . . . . . . . . . . . . . . . . . . . . . . . . . . T T T T T T T T . . .    
1109	. . . . . . . . . . . . . . . . . . . . . . . . . . . . . . . . . . . . . . . . . . . . . . . . . . . . . . . . . . . . . . . . . . . T T T T T T T T . . .    
1110	. . . . . . . . . . . . . . . . . . . . . . . . . . . . . . . . . . . . . . . . . . . . . . . . . . . . . . . . . . . . . . . . . . . T T T T T T T T . . .    
1111	. . . . . . . . . . . . . . . . . . . . . . . . . . . . . . . . . . . . . . . . . . . . . . . . . . . . . . . . . . . . . . . . . . . G G G G G G G G . . .    
1112	. . . . . . . . . . . . . . . . . . . . . . . . . . . . . . . . . . . . . . . . . . . . . . . . . . . . . . . . . . . . . . . . . . . G G G G G G G G . . .    
1113	. . . . . . . . . . . . . . . . . . . . . . . . . . . . . . . . . . . . . . . . . . . . . . . . . . . . . . . . . . . . . . . . . . . T T T T T T T T . . .    
1114	. . . . . . . . . . . . . . . . . . . . . . . . . . . . . . . . . . . . . . . . . . . . . . . . . . . . . . . . . . . . . . . . . . . T T T T T T T T . . .    
1115	. . . . . . . . . . . . . . . . . . . . . . . . . . . . . . . . . . . . . . . . . . . . . . . . . . . . . . . . . . . . . . . . . . . T T T T T T T T . . .    
1116	. . . . . . . . . . . . . . . . . . . . . . . . . . . . . . . . . . . . . . . . . . . . . . . . . . . . . . . . . . . . . . . . . . . G G G G G G G G . . .    
1117	. . . . . . . . . . . . . . . . . . . . . . . . . . . . . . . . . . . . . . . . . . . . . . . . . . . . . . . . . . . . . . . . . . . T T T T T T T T . . .    
1118	. . . . . . . . . . . . . . . . . . . . . . . . . . . . . . . . . . . . . . . . . . . . . . . . . . . . . . . . . . . . . . . . . . . A A A A A A A A . . .    
1119	. . . . . . . . . . . . . . . . . . . . . . . . . . . . . . . . . . . . . . . . . . . . . . . . . . . . . . . . . . . . . . . . . . . G G G G G G G G . . .    
1120	. . . . . . . . . . . . . . . . . . . . . . . . . . . . . . . . . . . . . . . . . . . . . . . . . . . . . . . . . . . . . . . . . . . C C C C C C C C . . .    
1121	. . . . . . . . . . . . . . . . . . . . . . . . . . . . . . . . . . . . . . . . . . . . . . . . . . . . . . . . . . . . . . . . . . . A A A A A A A A . . .    
1122	. . . . . . . . . . . . . . . . . . . . . . . . . . . . . . . . . . . . . . . . . . . . . . . . . . . . . . . . . . . . . . . . . . . G G G G G G G G . . .    
1123	. . . . . . . . . . . . . . . . . . . . . . . . . . . . . . . . . . . . . . . . . . . . . . . . . . . . . . . . . . . . . . . . . . . A A A A A A A A A . .    
1124	. . . . . . . . . . . . . . . . . . . . . . . . . . . . . . . . . . . . . . . . . . . . . . . . . . . . . . . . . . . . . . . . . . . T T T T T T T T T T .    
1125	. . . . . . . . . . . . . . . . . . . . . . . . . . . . . . . . . . . . . . . . . . . . . . . . . . . . . . . . . . . . . . . . . . . A A A A A A A A A A .    
1126	. . . . . . . . . . . . . . . . . . . . . . . . . . . . . . . . . . . . . . . . . . . . . . . . . . . . . . . . . . . . . . . . . . . T T T T T T T T T T .    
1127	. . . . . . . . . . . . . . . . . . . . . . . . . . . . . . . . . . . . . . . . . . . . . . . . . . . . . . . . . . . . . . . . . . . T T T T T T T T T T .    
1128	. . . . . . . . . . . . . . . . . . . . . . . . . . . . . . . . . . . . . . . . . . . . . . . . . . . . . . . . . . . . . . . . . . . G G G G G G G G G G .    
1129	. . . . . . . . . . . . . . . . . . . . . . . . . . . . . . . . . . . . . . . . . . . . . . . . . . . . . . . . . . . . . . . . . . . G G G G G G G G G G .    
1130	. . . . . . . . . . . . . . . . . . . . . . . . . . . . . . . . . . . . . . . . . . . . . . . . . . . . . . . . . . . . . . . . . . . T T T T T T T T T T .    
1131	. . . . . . . . . . . . . . . . . . . . . . . . . . . . . . . . . . . . . . . . . . . . . . . . . . . . . . . . . . . . . . . . . . . T T T T T T T T T T .    
1132	. . . . . . . . . . . . . . . . . . . . . . . . . . . . . . . . . . . . . . . . . . . . . . . . . . . . . . . . . . . . . . . . . . . C C C C C C C C C C .    
1133	. . . . . . . . . . . . . . . . . . . . . . . . . . . . . . . . . . . . . . . . . . . . . . . . . . . . . . . . . . . . . . . . . . . C C C C C C C C C C .    
1134	. . . . . . . . . . . . . . . . . . . . . . . . . . . . . . . . . . . . . . . . . . . . . . . . . . . . . . . . . . . . . . . . . . . A A A A A A A A A A .    
1135	. . . . . . . . . . . . . . . . . . . . . . . . . . . . . . . . . . . . . . . . . . . . . . . . . . . . . . . . . . . . . . . . . . . T T T T T T T T T T .    
1136	. . . . . . . . . . . . . . . . . . . . . . . . . . . . . . . . . . . . . . . . . . . . . . . . . . . . . . . . . . . . . . . . . . . G G G G G G G G G G .    
1137	. . . . . . . . . . . . . . . . . . . . . . . . . . . . . . . . . . . . . . . . . . . . . . . . . . . . . . . . . . . . . . . . . . . C C C C C C C C C C .    
1138	. . . . . . . . . . . . . . . . . . . . . . . . . . . . . . . . . . . . . . . . . . . . . . . . . . . . . . . . . . . . . . . . . . . T T T T T T T T T T .    
1139	. . . . . . . . . . . . . . . . . . . . . . . . . . . . . . . . . . . . . . . . . . . . . . . . . . . . . . . . . . . . . . . . . . . C C C C C C C C C C .    
1140	. . . . . . . . . . . . . . . . . . . . . . . . . . . . . . . . . . . . . . . . . . . . . . . . . . . . . . . . . . . . . . . . . . . A A A A A A A A A A .    
1141	. . . . . . . . . . . . . . . . . . . . . . . . . . . . . . . . . . . . . . . . . . . . . . . . . . . . . . . . . . . . . . . . . . . T T T T T T T T T T .    
1142	. . . . . . . . . . . . . . . . . . . . . . . . . . . . . . . . . . . . . . . . . . . . . . . . . . . . . . . . . . . . . . . . . . . C C C C C C C C C C .    
1143	. . . . . . . . . . . . . . . . . . . . . . . . . . . . . . . . . . . . . . . . . . . . . . . . . . . . . . . . . . . . . . . . . . . C C C C C C C C C C .    
1144	. . . . . . . . . . . . . . . . . . . . . . . . . . . . . . . . . . . . . . . . . . . . . . . . . . . . . . . . . . . . . . . . . . . A A A A A A A A A A .    
1145	. . . . . . . . . . . . . . . . . . . . . . . . . . . . . . . . . . . . . . . . . . . . . . . . . . . . . . . . . . . . . . . . . . . A A A A A A A A A A .    
1146	. . . . . . . . . . . . . . . . . . . . . . . . . . . . . . . . . . . . . . . . . . . . . . . . . . . . . . . . . . . . . . . . . . . C C C C C C C C C C .    
1147	. . . . . . . . . . . . . . . . . . . . . . . . . . . . . . . . . . . . . . . . . . . . . . . . . . . . . . . . . . . . . . . . . . . T T T T T T T T T T .    
1148	. . . . . . . . . . . . . . . . . . . . . . . . . . . . . . . . . . . . . . . . . . . . . . . . . . . . . . . . . . . . . . . . . . . C C C C C C C C C C .    
1149	. . . . . . . . . . . . . . . . . . . . . . . . . . . . . . . . . . . . . . . . . . . . . . . . . . . . . . . . . . . . . . . . . . . C C C C C C C C C C .    
1150	. . . . . . . . . . . . . . . . . . . . . . . . . . . . . . . . . . . . . . . . . . . . . . . . . . . . . . . . . . . . . . . . . . . A A A A A A A A A A .    
1151	. . . . . . . . . . . . . . . . . . . . . . . . . . . . . . . . . . . . . . . . . . . . . . . . . . . . . . . . . . . . . . . . . . . C C C C C C C C C C .    
1152	. . . . . . . . . . . . . . . . . . . . . . . . . . . . . . . . . . . . . . . . . . . . . . . . . . . . . . . . . . . . . . . . . . . C C C C C C C C C C .    
1153	. . . . . . . . . . . . . . . . . . . . . . . . . . . . . . . . . . . . . . . . . . . . . . . . . . . . . . . . . . . . . . . . . . . A A A A A A A A A A .    
1154	. . . . . . . . . . . . . . . . . . . . . . . . . . . . . . . . . . . . . . . . . . . . . . . . . . . . . . . . . . . . . . . . . . . C C C C C C C C C C .    
1155	. . . . . . . . . . . . . . . . . . . . . . . . . . . . . . . . . . . . . . . . . . . . . . . . . . . . . . . . . . . . . . . . . . . C C C C C C C C C C .    
1156	. . . . . . . . . . . . . . . . . . . . . . . . . . . . . . . . . . . . . . . . . . . . . . . . . . . . . . . . . . . . . . . . . . . T T T T T T T T T T .    
1157	. . . . . . . . . . . . . . . . . . . . . . . . . . . . . . . . . . . . . . . . . . . . . . . . . . . . . . . . . . . . . . . . . . . T T T T T T T T T T .    
1158	. . . . . . . . . . . . . . . . . . . . . . . . . . . . . . . . . . . . . . . . . . . . . . . . . . . . . . . . . . . . . . . . . . . G G G G G G G G G G .    
1159	. . . . . . . . . . . . . . . . . . . . . . . . . . . . . . . . . . . . . . . . . . . . . . . . . . . . . . . . . . . . . . . . . . . A A A A A A A A A A .    
1160	. . . . . . . . . . . . . . . . . . . . . . . . . . . . . . . . . . . . . . . . . . . . . . . . . . . . . . . . . . . . . . . . . . . G G G G G G G G G G .    
1161	. . . . . . . . . . . . . . . . . . . . . . . . . . . . . . . . . . . . . . . . . . . . . . . . . . . . . . . . . . . . . . . . . . . G G G G G G G G G G .    
1162	. . . . . . . . . . . . . . . . . . . . . . . . . . . . . . . . . . . . . . . . . . . . . . . . . . . . . . . . . . . . . . . . . . . T T T T T T T T T T .    
1163	. . . . . . . . . . . . . . . . . . . . . . . . . . . . . . . . . . . . . . . . . . . . . . . . . . . . . . . . . . . . . . . . . . . C C C C C C C C C C .    
1164	. . . . . . . . . . . . . . . . . . . . . . . . . . . . . . . . . . . . . . . . . . . . . . . . . . . . . . . . . . . . . . . . . . . C C C C C C C C C C .    
1165	. . . . . . . . . . . . . . . . . . . . . . . . . . . . . . . . . . . . . . . . . . . . . . . . . . . . . . . . . . . . . . . . . . . T T T T T T T T T T .    
1166	. . . . . . . . . . . . . . . . . . . . . . . . . . . . . . . . . . . . . . . . . . . . . . . . . . . . . . . . . . . . . . . . . . . C C C C C C C C C C .    
1167	. . . . . . . . . . . . . . . . . . . . . . . . . . . . . . . . . . . . . . . . . . . . . . . . . . . . . . . . . . . . . . . . . . . A A A A A A A A A A .    
1168	. . . . . . . . . . . . . . . . . . . . . . . . . . . . . . . . . . . . . . . . . . . . . . . . . . . . . . . . . . . . . . . . . . . T T T T T T T T T T .    
1169	. . . . . . . . . . . . . . . . . . . . . . . . . . . . . . . . . . . . . . . . . . . . . . . . . . . . . . . . . . . . . . . . . . . A A A A A A A A A A .    
1170	. . . . . . . . . . . . . . . . . . . . . . . . . . . . . . . . . . . . . . . . . . . . . . . . . . . . . . . . . . . . . . . . . . . G G G G G G G G G G .    
1171	. . . . . . . . . . . . . . . . . . . . . . . . . . . . . . . . . . . . . . . . . . . . . . . . . . . . . . . . . . . . . . . . . . . T T T T T T T T T T .    
1172	. . . . . . . . . . . . . . . . . . . . . . . . . . . . . . . . . . . . . . . . . . . . . . . . . . . . . . . . . . . . . . . . . . . C C C C C C C C C C .    
1173	. . . . . . . . . . . . . . . . . . . . . . . . . . . . . . . . . . . . . . . . . . . . . . . . . . . . . . . . . . . . . . . . . . . C C C C C C C C C C .    
1174	. . . . . . . . . . . . . . . . . . . . . . . . . . . . . . . . . . . . . . . . . . . . . . . . . . . . . . . . . . . . . . . . . . . A A A A A A A A A A .    
1175	. . . . . . . . . . . . . . . . . . . . . . . . . . . . . . . . . . . . . . . . . . . . . . . . . . . . . . . . . . . . . . . . . . . C C C C C C C C C C .    
1176	. . . . . . . . . . . . . . . . . . . . . . . . . . . . . . . . . . . . . . . . . . . . . . . . . . . . . . . . . . . . . . . . . . . A A A A A A A A A A .    
1177	. . . . . . . . . . . . . . . . . . . . . . . . . . . . . . . . . . . . . . . . . . . . . . . . . . . . . . . . . . . . . . . . . . . G G G G G G G G G G .    
1178	. . . . . . . . . . . . . . . . . . . . . . . . . . . . . . . . . . . . . . . . . . . . . . . . . . . . . . . . . . . . . . . . . . . G G G G G G G G G G .    
1179	. . . . . . . . . . . . . . . . . . . . . . . . . . . . . . . . . . . . . . . . . . . . . . . . . . . . . . . . . . . . . . . . . . . C C C C C C C C C C .    
1180	. . . . . . . . . . . . . . . . . . . . . . . . . . . . . . . . . . . . . . . . . . . . . . . . . . . . . . . . . . . . . . . . . . . A A A A A A A A A A .    
1181	. . . . . . . . . . . . . . . . . . . . . . . . . . . . . . . . . . . . . . . . . . . . . . . . . . . . . . . . . . . . . . . . . . . C C C C C C C C C C .    
1182	. . . . . . . . . . . . . . . . . . . . . . . . . . . . . . . . . . . . . . . . . . . . . . . . . . . . . . . . . . . . . . . . . . . A A A A A A A A A A .    
1183	. . . . . . . . . . . . . . . . . . . . . . . . . . . . . . . . . . . . . . . . . . . . . . . . . . . . . . . . . . . . . . . . . . . C C C C C C C C C C .    
1184	. . . . . . . . . . . . . . . . . . . . . . . . . . . . . . . . . . . . . . . . . . . . . . . . . . . . . . . . . . . . . . . . . . . A A A A A A A A A A .    
1185	. . . . . . . . . . . . . . . . . . . . . . . . . . . . . . . . . . . . . . . . . . . . . . . . . . . . . . . . . . . . . . . . . . . G G G G G G G G G G .    
1186	. . . . . . . . . . . . . . . . . . . . . . . . . . . . . . . . . . . . . . . . . . . . . . . . . . . . . . . . . . . . . . . . . . . C C C C C C C C C C .    
1187	. . . . . . . . . . . . . . . . . . . . . . . . . . . . . . . . . . . . . . . . . . . . . . . . . . . . . . . . . . . . . . . . . . . A A A A A A A A A A .    
1188	. . . . . . . . . . . . . . . . . . . . . . . . . . . . . . . . . . . . . . . . . . . . . . . . . . . . . . . . . . . . . . . . . . . G G G G G G G G G G .    
1189	. . . . . . . . . . . . . . . . . . . . . . . . . . . . . . . . . . . . . . . . . . . . . . . . . . . . . . . . . . . . . . . . . . . G G G G G G G G G G .    
1190	. . . . . . . . . . . . . . . . . . . . . . . . . . . . . . . . . . . . . . . . . . . . . . . . . . . . . . . . . . . . . . . . . . . G G G G G G G G G G .    
1191	. . . . . . . . . . . . . . . . . . . . . . . . . . . . . . . . . . . . . . . . . . . . . . . . . . . . . . . . . . . . . . . . . . . T T T T T T T T T T .    
1192	. . . . . . . . . . . . . . . . . . . . . . . . . . . . . . . . . . . . . . . . . . . . . . . . . . . . . . . . . . . . . . . . . . . G G G G G G G G G G .    
1193	. . . . . . . . . . . . . . . . . . . . . . . . . . . . . . . . . . . . . . . . . . . . . . . . . . . . . . . . . . . . . . . . . . . A A A A A A A A A - .    
1194	. . . . . . . . . . . . . . . . . . . . . . . . . . . . . . . . . . . . . . . . . . . . . . . . . . . . . . . . . . . . . . . . . . . G G G G G G G G G G .    
1195	. . . . . . . . . . . . . . . . . . . . . . . . . . . . . . . . . . . . . . . . . . . . . . . . . . . . . . . . . . . . . . . . . . . C C C C C C C C C C .    
1196	. . . . . . . . . . . . . . . . . . . . . . . . . . . . . . . . . . . . . . . . . . . . . . . . . . . . . . . . . . . . . . . . . . . G G G G G G G G G G .    
1197	. . . . . . . . . . . . . . . . . . . . . . . . . . . . . . . . . . . . . . . . . . . . . . . . . . . . . . . . . . . . . . . . . . . C C C C C C C C C C .    
1198	. . . . . . . . . . . . . . . . . . . . . . . . . . . . . . . . . . . . . . . . . . . . . . . . . . . . . . . . . . . . . . . . . . . T T T T T T T T T T .    
1199	. . . . . . . . . . . . . . . . . . . . . . . . . . . . . . . . . . . . . . . . . . . . . . . . . . . . . . . . . . . . . . . . . . . C C C C C C C C C C .    
1200	. . . . . . . . . . . . . . . . . . . . . . . . . . . . . . . . . . . . . . . . . . . . . . . . . . . . . . . . . . . . . . . . . . . C C C C C C C C C C .    
1201	. . . . . . . . . . . . . . . . . . . . . . . . . . . . . . . . . . . . . . . . . . . . . . . . . . . . . . . . . . . . . . . . . . . A A A A A A A A A A .    
1202	. . . . . . . . . . . . . . . . . . . . . . . . . . . . . . . . . . . . . . . . . . . . . . . . . . . . . . . . . . . . . . . . . . . G G G G G G G G G G .    
1203	. . . . . . . . . . . . . . . . . . . . . . . . . . . . . . . . . . . . . . . . . . . . . . . . . . . . . . . . . . . . . . . . . . . G G G G G G G G G G .    
1204	. . . . . . . . . . . . . . . . . . . . . . . . . . . . . . . . . . . . . . . . . . . . . . . . . . . . . . . . . . . . . . . . . . . G G G G G G G G G G .    
1205	. . . . . . . . . . . . . . . . . . . . . . . . . . . . . . . . . . . . . . . . . . . . . . . . . . . . . . . . . . . . . . . . . . . C C C C C C C C C C .    
1206	. . . . . . . . . . . . . . . . . . . . . . . . . . . . . . . . . . . . . . . . . . . . . . . . . . . . . . . . . . . . . . . . . . . C C C C C C C C C C .    
1207	. . . . . . . . . . . . . . . . . . . . . . . . . . . . . . . . . . . . . . . . . . . . . . . . . . . . . . . . . . . . . . . . . . . A A A A A A A A A A .    
1208	. . . . . . . . . . . . . . . . . . . . . . . . . . . . . . . . . . . . . . . . . . . . . . . . . . . . . . . . . . . . . . . . . . . G G G G G G G G G G .    
1209	. . . . . . . . . . . . . . . . . . . . . . . . . . . . . . . . . . . . . . . . . . . . . . . . . . . . . . . . . . . . . . . . . . . G G G G G G G G G G .    
1210	. . . . . . . . . . . . . . . . . . . . . . . . . . . . . . . . . . . . . . . . . . . . . . . . . . . . . . . . . . . . . . . . . . . C C C C C C C C C C .    
1211	. . . . . . . . . . . . . . . . . . . . . . . . . . . . . . . . . . . . . . . . . . . . . . . . . . . . . . . . . . . . . . . . . . . C C C C C C C C C C .    
1212	. . . . . . . . . . . . . . . . . . . . . . . . . . . . . . . . . . . . . . . . . . . . . . . . . . . . . . . . . . . . . . . . . . . A A A A A A A A A A .    
1213	. . . . . . . . . . . . . . . . . . . . . . . . . . . . . . . . . . . . . . . . . . . . . . . . . . . . . . . . . . . . . . . . . . . C C C C C C C C C C .    
1214	. . . . . . . . . . . . . . . . . . . . . . . . . . . . . . . . . . . . . . . . . . . . . . . . . . . . . . . . . . . . . . . . . . . T T T T T T T T T T .    
1215	. . . . . . . . . . . . . . . . . . . . . . . . . . . . . . . . . . . . . . . . . . . . . . . . . . . . . . . . . . . . . . . . . . . C C C C C C C C C C .    
1216	. . . . . . . . . . . . . . . . . . . . . . . . . . . . . . . . . . . . . . . . . . . . . . . . . . . . . . . . . . . . . . . . . . . T T T T T T T T T T .    
1217	. . . . . . . . . . . . . . . . . . . . . . . . . . . . . . . . . . . . . . . . . . . . . . . . . . . . . . . . . . . . . . . . . . . G G G G G G G G G G .    
1218	. . . . . . . . . . . . . . . . . . . . . . . . . . . . . . . . . . . . . . . . . . . . . . . . . . . . . . . . . . . . . . . . . . . G G G G G G G G G G .    
1219	. . . . . . . . . . . . . . . . . . . . . . . . . . . . . . . . . . . . . . . . . . . . . . . . . . . . . . . . . . . . . . . . . . . A A A A A A A A A A .    
1220	. . . . . . . . . . . . . . . . . . . . . . . . . . . . . . . . . . . . . . . . . . . . . . . . . . . . . . . . . . . . . . . . . . . C C C C C C C C C C .    
1221	. . . . . . . . . . . . . . . . . . . . . . . . . . . . . . . . . . . . . . . . . . . . . . . . . . . . . . . . . . . . . . . . . . . C C C C C C C C C C .    
1222	. . . . . . . . . . . . . . . . . . . . . . . . . . . . . . . . . . . . . . . . . . . . . . . . . . . . . . . . . . . . . . . . . . . T T T T T T T T T T .    
1223	. . . . . . . . . . . . . . . . . . . . . . . . . . . . . . . . . . . . . . . . . . . . . . . . . . . . . . . . . . . . . . . . . . . G G G G G G G G G G .    
1224	. . . . . . . . . . . . . . . . . . . . . . . . . . . . . . . . . . . . . . . . . . . . . . . . . . . . . . . . . . . . . . . . . . . G G G G G G G G G G .    
1225	. . . . . . . . . . . . . . . . . . . . . . . . . . . . . . . . . . . . . . . . . . . . . . . . . . . . . . . . . . . . . . . . . . . C C C C C C C C C C .    
1226	. . . . . . . . . . . . . . . . . . . . . . . . . . . . . . . . . . . . . . . . . . . . . . . . . . . . . . . . . . . . . . . . . . . T T T T T T T T T T .    
1227	. . . . . . . . . . . . . . . . . . . . . . . . . . . . . . . . . . . . . . . . . . . . . . . . . . . . . . . . . . . . . . . . . . . G G G G G G G G G G .    
1228	. . . . . . . . . . . . . . . . . . . . . . . . . . . . . . . . . . . . . . . . . . . . . . . . . . . . . . . . . . . . . . . . . . . T T T T T T T T T T .    
1229	. . . . . . . . . . . . . . . . . . . . . . . . . . . . . . . . . . . . . . . . . . . . . . . . . . . . . . . . . . . . . . . . . . . T T T T T T T T T T .    
1230	. . . . . . . . . . . . . . . . . . . . . . . . . . . . . . . . . . . . . . . . . . . . . . . . . . . . . . . . . . . . . . . . . . . A A A A A A A A A A .    
1231	. . . . . . . . . . . . . . . . . . . . . . . . . . . . . . . . . . . . . . . . . . . . . . . . . . . . . . . . . . . . . . . . . . . A A A A A A A A A A .    
1232	. . . . . . . . . . . . . . . . . . . . . . . . . . . . . . . . . . . . . . . . . . . . . . . . . . . . . . . . . . . . . . . . . . . G G G G G G G G G G .    
1233	. . . . . . . . . . . . . . . . . . . . . . . . . . . . . . . . . . . . . . . . . . . . . . . . . . . . . . . . . . . . . . . . . . . C C C C C C C C C C .    
1234	. . . . . . . . . . . . . . . . . . . . . . . . . . . . . . . . . . . . . . . . . . . . . . . . . . . . . . . . . . . . . . . . . . . A A A A A A A A A A .    
1235	. . . . . . . . . . . . . . . . . . . . . . . . . . . . . . . . . . . . . . . . . . . . . . . . . . . . . . . . . . . . . . . . . . . G G G G G G G G G G .    
1236	. . . . . . . . . . . . . . . . . . . . . . . . . . . . . . . . . . . . . . . . . . . . . . . . . . . . . . . . . . . . . . . . . . . G G G G G G G G G G .    
1237	. . . . . . . . . . . . . . . . . . . . . . . . . . . . . . . . . . . . . . . . . . . . . . . . . . . . . . . . . . . . . . . . . . . A A A A A A A A A A .    
1238	. . . . . . . . . . . . . . . . . . . . . . . . . . . . . . . . . . . . . . . . . . . . . . . . . . . . . . . . . . . . . . . . . . . T T T T T T T T T T .    
1239	. . . . . . . . . . . . . . . . . . . . . . . . . . . . . . . . . . . . . . . . . . . . . . . . . . . . . . . . . . . . . . . . . . . A A A A A A A A A A .    
1240	. . . . . . . . . . . . . . . . . . . . . . . . . . . . . . . . . . . . . . . . . . . . . . . . . . . . . . . . . . . . . . . . . . . G G G G G G G G G G .    
1241	. . . . . . . . . . . . . . . . . . . . . . . . . . . . . . . . . . . . . . . . . . . . . . . . . . . . . . . . . . . . . . . . . . . C C C C C C C C C C .    
1242	. . . . . . . . . . . . . . . . . . . . . . . . . . . . . . . . . . . . . . . . . . . . . . . . . . . . . . . . . . . . . . . . . . . A A A A A A A A A A .    
1243	. . . . . . . . . . . . . . . . . . . . . . . . . . . . . . . . . . . . . . . . . . . . . . . . . . . . . . . . . . . . . . . . . . . T T T T T T T T T T T    
1244	. . . . . . . . . . . . . . . . . . . . . . . . . . . . . . . . . . . . . . . . . . . . . . . . . . . . . . . . . . . . . . . . . . . G G G G G G G G G G G    
1245	. . . . . . . . . . . . . . . . . . . . . . . . . . . . . . . . . . . . . . . . . . . . . . . . . . . . . . . . . . . . . . . . . . . G G G G G G G G G G G    
1246	. . . . . . . . . . . . . . . . . . . . . . . . . . . . . . . . . . . . . . . . . . . . . . . . . . . . . . . . . . . . . . . . . . . T T T T T T T T T T T    
1247	. . . . . . . . . . . . . . . . . . . . . . . . . . . . . . . . . . . . . . . . . . . . . . . . . . . . . . . . . . . . . . . . . . . T T T T T T T T T T T    
1248	. . . . . . . . . . . . . . . . . . . . . . . . . . . . . . . . . . . . . . . . . . . . . . . . . . . . . . . . . . . . . . . . . . . A A A A A A A A A A A    
1249	. . . . . . . . . . . . . . . . . . . . . . . . . . . . . . . . . . . . . . . . . . . . . . . . . . . . . . . . . . . . . . . . . . . T T T T T T T T T T T    
1250	. . . . . . . . . . . . . . . . . . . . . . . . . . . . . . . . . . . . . . . . . . . . . . . . . . . . . . . . . . . . . . . . . . . T T T T T T T T T T T    
1251	. . . . . . . . . . . . . . . . . . . . . . . . . . . . . . . . . . . . . . . . . . . . . . . . . . . . . . . . . . . . . . . . . . . C C C C C C C C C C C    
1252	. . . . . . . . . . . . . . . . . . . . . . . . . . . . . . . . . . . . . . . . . . . . . . . . . . . . . . . . . . . . . . . . . . . C C C C C C C C C C C    
1253	. . . . . . . . . . . . . . . . . . . . . . . . . . . . . . . . . . . . . . . . . . . . . . . . . . . . . . . . . . . . . . . . . . . C C C C C C C C C C C    
1254	. . . . . . . . . . . . . . . . . . . . . . . . . . . . . . . . . . . . . . . . . . . . . . . . . . . . . . . . . . . . . . . . . . . G G G G G G G G G G G    
1255	. . . . . . . . . . . . . . . . . . . . . . . . . . . . . . . . . . . . . . . . . . . . . . . . . . . . . . . . . . . . . . . . . . . T T T T T T T T T T T    
1256	. . . . . . . . . . . . . . . . . . . . . . . . . . . . . . . . . . . . . . . . . . . . . . . . . . . . . . . . . . . . . . . . . . . T T T T T T T T T T T    
1257	. . . . . . . . . . . . . . . . . . . . . . . . . . . . . . . . . . . . . . . . . . . . . . . . . . . . . . . . . . . . . . . . . . . G G G G G G G G G G G    
1258	. . . . . . . . . . . . . . . . . . . . . . . . . . . . . . . . . . . . . . . . . . . . . . . . . . . . . . . . . . . . . . . . . . . G G G G G G G G G G G    
1259	. . . . . . . . . . . . . . . . . . . . . . . . . . . . . . . . . . . . . . . . . . . . . . . . . . . . . . . . . . . . . . . . . . . T T T T T T T T T T T    
1260	. . . . . . . . . . . . . . . . . . . . . . . . . . . . . . . . . . . . . . . . . . . . . . . . . . . . . . . . . . . . . . . . . . . C C C C C C C C C C C    
1261	. . . . . . . . . . . . . . . . . . . . . . . . . . . . . . . . . . . . . . . . . . . . . . . . . . . . . . . . . . . . . . . . . . . A A A A A A A A A A A    
1262	. . . . . . . . . . . . . . . . . . . . . . . . . . . . . . . . . . . . . . . . . . . . . . . . . . . . . . . . . . . . . . . . . . . G G G G G G G G G G G    
1263	. . . . . . . . . . . . . . . . . . . . . . . . . . . . . . . . . . . . . . . . . . . . . . . . . . . . . . . . . . . . . . . . . . . G G G G G G G G G G G    
1264	. . . . . . . . . . . . . . . . . . . . . . . . . . . . . . . . . . . . . . . . . . . . . . . . . . . . . . . . . . . . . . . . . . . G G G G G G G G G G G    
1265	. . . . . . . . . . . . . . . . . . . . . . . . . . . . . . . . . . . . . . . . . . . . . . . . . . . . . . . . . . . . . . . . . . . G G G G G G G G G G G    
1266	. . . . . . . . . . . . . . . . . . . . . . . . . . . . . . . . . . . . . . . . . . . . . . . . . . . . . . . . . . . . . . . . . . . A A A A A A A A A A A    
1267	. . . . . . . . . . . . . . . . . . . . . . . . . . . . . . . . . . . . . . . . . . . . . . . . . . . . . . . . . . . . . . . . . . . A A A A A A A A A A A    
1268	. . . . . . . . . . . . . . . . . . . . . . . . . . . . . . . . . . . . . . . . . . . . . . . . . . . . . . . . . . . . . . . . . . . A A A A A A A A A A A    
1269	. . . . . . . . . . . . . . . . . . . . . . . . . . . . . . . . . . . . . . . . . . . . . . . . . . . . . . . . . . . . . . . . . . . G G G G G G G G G G G    
1270	. . . . . . . . . . . . . . . . . . . . . . . . . . . . . . . . . . . . . . . . . . . . . . . . . . . . . . . . . . . . . . . . . . . C C C C C C C C C C C    
1271	. . . . . . . . . . . . . . . . . . . . . . . . . . . . . . . . . . . . . . . . . . . . . . . . . . . . . . . . . . . . . . . . . . . T T T T T T T T T T T    
1272	. . . . . . . . . . . . . . . . . . . . . . . . . . . . . . . . . . . . . . . . . . . . . . . . . . . . . . . . . . . . . . . . . . . G G G G G G G G G G G    
1273	. . . . . . . . . . . . . . . . . . . . . . . . . . . . . . . . . . . . . . . . . . . . . . . . . . . . . . . . . . . . . . . . . . . C C C C C C C C C C C    
1274	. . . . . . . . . . . . . . . . . . . . . . . . . . . . . . . . . . . . . . . . . . . . . . . . . . . . . . . . . . . . . . . . . . . A A A A A A A A A A A    
1275	. . . . . . . . . . . . . . . . . . . . . . . . . . . . . . . . . . . . . . . . . . . . . . . . . . . . . . . . . . . . . . . . . . . G G G G G G G G G G G    
1276	. . . . . . . . . . . . . . . . . . . . . . . . . . . . . . . . . . . . . . . . . . . . . . . . . . . . . . . . . . . . . . . . . . . A A A A A A A A A A A    
1277	. . . . . . . . . . . . . . . . . . . . . . . . . . . . . . . . . . . . . . . . . . . . . . . . . . . . . . . . . . . . . . . . . . . C C C C C C C C C C C    
1278	. . . . . . . . . . . . . . . . . . . . . . . . . . . . . . . . . . . . . . . . . . . . . . . . . . . . . . . . . . . . . . . . . . . T T T T T T T T T T T    
1279	. . . . . . . . . . . . . . . . . . . . . . . . . . . . . . . . . . . . . . . . . . . . . . . . . . . . . . . . . . . . . . . . . . . C C C C C C C C C C C    
1280	. . . . . . . . . . . . . . . . . . . . . . . . . . . . . . . . . . . . . . . . . . . . . . . . . . . . . . . . . . . . . . . . . . . C C C C C C C C C C C    
1281	. . . . . . . . . . . . . . . . . . . . . . . . . . . . . . . . . . . . . . . . . . . . . . . . . . . . . . . . . . . . . . . . . . . . C C C C C C C C C C    
1282	. . . . . . . . . . . . . . . . . . . . . . . . . . . . . . . . . . . . . . . . . . . . . . . . . . . . . . . . . . . . . . . . . . . . T T T T T T T T T T    
1283	. . . . . . . . . . . . . . . . . . . . . . . . . . . . . . . . . . . . . . . . . . . . . . . . . . . . . . . . . . . . . . . . . . . . G G G G G G G G G G    
1284	. . . . . . . . . . . . . . . . . . . . . . . . . . . . . . . . . . . . . . . . . . . . . . . . . . . . . . . . . . . . . . . . . . . . G G G G G G G G G G    
1285	. . . . . . . . . . . . . . . . . . . . . . . . . . . . . . . . . . . . . . . . . . . . . . . . . . . . . . . . . . . . . . . . . . . . C C C C C C C C C C    
1286	. . . . . . . . . . . . . . . . . . . . . . . . . . . . . . . . . . . . . . . . . . . . . . . . . . . . . . . . . . . . . . . . . . . . A A A A A A A A A A    
1287	. . . . . . . . . . . . . . . . . . . . . . . . . . . . . . . . . . . . . . . . . . . . . . . . . . . . . . . . . . . . . . . . . . . . G G G G G G G G G G    
1288	. . . . . . . . . . . . . . . . . . . . . . . . . . . . . . . . . . . . . . . . . . . . . . . . . . . . . . . . . . . . . . . . . . . . T T T T T T T T T T    
1289	. . . . . . . . . . . . . . . . . . . . . . . . . . . . . . . . . . . . . . . . . . . . . . . . . . . . . . . . . . . . . . . . . . . . T T T T T T T T T T    
1290	. . . . . . . . . . . . . . . . . . . . . . . . . . . . . . . . . . . . . . . . . . . . . . . . . . . . . . . . . . . . . . . . . . . . G G G G G G G G G G    
1291	. . . . . . . . . . . . . . . . . . . . . . . . . . . . . . . . . . . . . . . . . . . . . . . . . . . . . . . . . . . . . . . . . . . . T T T T T T T T T T    
1292	. . . . . . . . . . . . . . . . . . . . . . . . . . . . . . . . . . . . . . . . . . . . . . . . . . . . . . . . . . . . . . . . . . . . A A A A A A A A A A    
1293	. . . . . . . . . . . . . . . . . . . . . . . . . . . . . . . . . . . . . . . . . . . . . . . . . . . . . . . . . . . . . . . . . . . . G G G G G G G G G G    
1294	. . . . . . . . . . . . . . . . . . . . . . . . . . . . . . . . . . . . . . . . . . . . . . . . . . . . . . . . . . . . . . . . . . . . A A A A A A A A A A    
1295	. . . . . . . . . . . . . . . . . . . . . . . . . . . . . . . . . . . . . . . . . . . . . . . . . . . . . . . . . . . . . . . . . . . . T T T T T T T T T T    
1296	. . . . . . . . . . . . . . . . . . . . . . . . . . . . . . . . . . . . . . . . . . . . . . . . . . . . . . . . . . . . . . . . . . . . G G G G G G G G G G    
1297	. . . . . . . . . . . . . . . . . . . . . . . . . . . . . . . . . . . . . . . . . . . . . . . . . . . . . . . . . . . . . . . . . . . . G G G G G G G G G G    
1298	. . . . . . . . . . . . . . . . . . . . . . . . . . . . . . . . . . . . . . . . . . . . . . . . . . . . . . . . . . . . . . . . . . . . T T T T T T T T T T    
1299	. . . . . . . . . . . . . . . . . . . . . . . . . . . . . . . . . . . . . . . . . . . . . . . . . . . . . . . . . . . . . . . . . . . . G G G G G G G G G G    
1300	. . . . . . . . . . . . . . . . . . . . . . . . . . . . . . . . . . . . . . . . . . . . . . . . . . . . . . . . . . . . . . . . . . . . G G G G G G G G G G    
1301	. . . . . . . . . . . . . . . . . . . . . . . . . . . . . . . . . . . . . . . . . . . . . . . . . . . . . . . . . . . . . . . . . . . . C C C C C C C C C C    
1302	. . . . . . . . . . . . . . . . . . . . . . . . . . . . . . . . . . . . . . . . . . . . . . . . . . . . . . . . . . . . . . . . . . . . C C C C C C C C C C    
1303	. . . . . . . . . . . . . . . . . . . . . . . . . . . . . . . . . . . . . . . . . . . . . . . . . . . . . . . . . . . . . . . . . . . . T T T T T T T T T T    
1304	. . . . . . . . . . . . . . . . . . . . . . . . . . . . . . . . . . . . . . . . . . . . . . . . . . . . . . . . . . . . . . . . . . . . C C C C C C C C C C    
1305	. . . . . . . . . . . . . . . . . . . . . . . . . . . . . . . . . . . . . . . . . . . . . . . . . . . . . . . . . . . . . . . . . . . . A A A A A A A A A A    
1306	. . . . . . . . . . . . . . . . . . . . . . . . . . . . . . . . . . . . . . . . . . . . . . . . . . . . . . . . . . . . . . . . . . . . G G G G G G G G G G    
1307	. . . . . . . . . . . . . . . . . . . . . . . . . . . . . . . . . . . . . . . . . . . . . . . . . . . . . . . . . . . . . . . . . . . . G G G G G G G G G G    
1308	. . . . . . . . . . . . . . . . . . . . . . . . . . . . . . . . . . . . . . . . . . . . . . . . . . . . . . . . . . . . . . . . . . . . A A A A A A A A A A    
1309	. . . . . . . . . . . . . . . . . . . . . . . . . . . . . . . . . . . . . . . . . . . . . . . . . . . . . . . . . . . . . . . . . . . . G G G G G G G G G G    
1310	. . . . . . . . . . . . . . . . . . . . . . . . . . . . . . . . . . . . . . . . . . . . . . . . . . . . . . . . . . . . . . . . . . . . G G G G G G G G G G    
1311	. . . . . . . . . . . . . . . . . . . . . . . . . . . . . . . . . . . . . . . . . . . . . . . . . . . . . . . . . . . . . . . . . . . . A A A A A A A A A A    
1312	. . . . . . . . . . . . . . . . . . . . . . . . . . . . . . . . . . . . . . . . . . . . . . . . . . . . . . . . . . . . . . . . . . . . G G G G G G G G G G    
1313	. . . . . . . . . . . . . . . . . . . . . . . . . . . . . . . . . . . . . . . . . . . . . . . . . . . . . . . . . . . . . . . . . . . . A A A A A A A A A A    
1314	. . . . . . . . . . . . . . . . . . . . . . . . . . . . . . . . . . . . . . . . . . . . . . . . . . . . . . . . . . . . . . . . . . . . C C C C C C C C C C    
1315	. . . . . . . . . . . . . . . . . . . . . . . . . . . . . . . . . . . . . . . . . . . . . . . . . . . . . . . . . . . . . . . . . . . . A A A A A A A A A A    
1316	. . . . . . . . . . . . . . . . . . . . . . . . . . . . . . . . . . . . . . . . . . . . . . . . . . . . . . . . . . . . . . . . . . . . G G G G G G G G G G    
1317	. . . . . . . . . . . . . . . . . . . . . . . . . . . . . . . . . . . . . . . . . . . . . . . . . . . . . . . . . . . . . . . . . . . . C C C C C C C C C C    
1318	. . . . . . . . . . . . . . . . . . . . . . . . . . . . . . . . . . . . . . . . . . . . . . . . . . . . . . . . . . . . . . . . . . . . A A A A A A A A A A    
1319	. . . . . . . . . . . . . . . . . . . . . . . . . . . . . . . . . . . . . . . . . . . . . . . . . . . . . . . . . . . . . . . . . . . . G G G G G G G G G G    
1320	. . . . . . . . . . . . . . . . . . . . . . . . . . . . . . . . . . . . . . . . . . . . . . . . . . . . . . . . . . . . . . . . . . . . G G G G G G G G G G    
1321	. . . . . . . . . . . . . . . . . . . . . . . . . . . . . . . . . . . . . . . . . . . . . . . . . . . . . . . . . . . . . . . . . . . . T T T T T T T T T T    
1322	. . . . . . . . . . . . . . . . . . . . . . . . . . . . . . . . . . . . . . . . . . . . . . . . . . . . . . . . . . . . . . . . . . . . A A A A A A A A A A    
1323	. . . . . . . . . . . . . . . . . . . . . . . . . . . . . . . . . . . . . . . . . . . . . . . . . . . . . . . . . . . . . . . . . . . . T T T T T T T T T T    
1324	. . . . . . . . . . . . . . . . . . . . . . . . . . . . . . . . . . . . . . . . . . . . . . . . . . . . . . . . . . . . . . . . . . . . T T T T T T T T T T    
1325	. . . . . . . . . . . . . . . . . . . . . . . . . . . . . . . . . . . . . . . . . . . . . . . . . . . . . . . . . . . . . . . . . . . . T T T T T T T T T T    
1326	. . . . . . . . . . . . . . . . . . . . . . . . . . . . . . . . . . . . . . . . . . . . . . . . . . . . . . . . . . . . . . . . . . . . C C C C C C C C C C    
1327	. . . . . . . . . . . . . . . . . . . . . . . . . . . . . . . . . . . . . . . . . . . . . . . . . . . . . . . . . . . . . . . . . . . . T T T T T T T T T T    
1328	. . . . . . . . . . . . . . . . . . . . . . . . . . . . . . . . . . . . . . . . . . . . . . . . . . . . . . . . . . . . . . . . . . . . C C C C C C C C C C    
1329	. . . . . . . . . . . . . . . . . . . . . . . . . . . . . . . . . . . . . . . . . . . . . . . . . . . . . . . . . . . . . . . . . . . . C C C C C C C C C C    
1330	. . . . . . . . . . . . . . . . . . . . . . . . . . . . . . . . . . . . . . . . . . . . . . . . . . . . . . . . . . . . . . . . . . . . A A A A A A A A A A    
1331	. . . . . . . . . . . . . . . . . . . . . . . . . . . . . . . . . . . . . . . . . . . . . . . . . . . . . . . . . . . . . . . . . . . . G G G G G G G G G G    
1332	. . . . . . . . . . . . . . . . . . . . . . . . . . . . . . . . . . . . . . . . . . . . . . . . . . . . . . . . . . . . . . . . . . . . G G G G G G G G G G    
1333	. . . . . . . . . . . . . . . . . . . . . . . . . . . . . . . . . . . . . . . . . . . . . . . . . . . . . . . . . . . . . . . . . . . . G G G G G G G G G G    
1334	. . . . . . . . . . . . . . . . . . . . . . . . . . . . . . . . . . . . . . . . . . . . . . . . . . . . . . . . . . . . . . . . . . . . A A A A A A A A A A    
1335	. . . . . . . . . . . . . . . . . . . . . . . . . . . . . . . . . . . . . . . . . . . . . . . . . . . . . . . . . . . . . . . . . . . . C C C C C C C C C C    
1336	. . . . . . . . . . . . . . . . . . . . . . . . . . . . . . . . . . . . . . . . . . . . . . . . . . . . . . . . . . . . . . . . . . . . A A A A A A A A A A    
1337	. . . . . . . . . . . . . . . . . . . . . . . . . . . . . . . . . . . . . . . . . . . . . . . . . . . . . . . . . . . . . . . . . . . . C C C C C C C C C C    
1338	. . . . . . . . . . . . . . . . . . . . . . . . . . . . . . . . . . . . . . . . . . . . . . . . . . . . . . . . . . . . . . . . . . . . G G G G G G G G G G    
1339	. . . . . . . . . . . . . . . . . . . . . . . . . . . . . . . . . . . . . . . . . . . . . . . . . . . . . . . . . . . . . . . . . . . . G G G G G G G G G G    
1340	. . . . . . . . . . . . . . . . . . . . . . . . . . . . . . . . . . . . . . . . . . . . . . . . . . . . . . . . . . . . . . . . . . . . T T T T T T T T T T    
1341	. . . . . . . . . . . . . . . . . . . . . . . . . . . . . . . . . . . . . . . . . . . . . . . . . . . . . . . . . . . . . . . . . . . . G G G G G G G G G G    
1342	. . . . . . . . . . . . . . . . . . . . . . . . . . . . . . . . . . . . . . . . . . . . . . . . . . . . . . . . . . . . . . . . . . . . A A A A A A A A A A    
1343	. . . . . . . . . . . . . . . . . . . . . . . . . . . . . . . . . . . . . . . . . . . . . . . . . . . . . . . . . . . . . . . . . . . . G G G G G G G G G G    
1344	. . . . . . . . . . . . . . . . . . . . . . . . . . . . . . . . . . . . . . . . . . . . . . . . . . . . . . . . . . . . . . . . . . . . A A A A A A A A A A    
1345	. . . . . . . . . . . . . . . . . . . . . . . . . . . . . . . . . . . . . . . . . . . . . . . . . . . . . . . . . . . . . . . . . . . . C C C C C C C C C C    
1346	. . . . . . . . . . . . . . . . . . . . . . . . . . . . . . . . . . . . . . . . . . . . . . . . . . . . . . . . . . . . . . . . . . . . T T T T T T T T T T    
1347	. . . . . . . . . . . . . . . . . . . . . . . . . . . . . . . . . . . . . . . . . . . . . . . . . . . . . . . . . . . . . . . . . . . . G G G G G G G G G G    
1348	. . . . . . . . . . . . . . . . . . . . . . . . . . . . . . . . . . . . . . . . . . . . . . . . . . . . . . . . . . . . . . . . . . . . T T T T T T T T T T    
1349	. . . . . . . . . . . . . . . . . . . . . . . . . . . . . . . . . . . . . . . . . . . . . . . . . . . . . . . . . . . . . . . . . . . . G G G G G G G G G G    
1350	. . . . . . . . . . . . . . . . . . . . . . . . . . . . . . . . . . . . . . . . . . . . . . . . . . . . . . . . . . . . . . . . . . . . C C C C C C C C C C    
1351	. . . . . . . . . . . . . . . . . . . . . . . . . . . . . . . . . . . . . . . . . . . . . . . . . . . . . . . . . . . . . . . . . . . . A A A A A A A A A A    
1352	. . . . . . . . . . . . . . . . . . . . . . . . . . . . . . . . . . . . . . . . . . . . . . . . . . . . . . . . . . . . . . . . . . . . G G G G G G G G G G    
1353	. . . . . . . . . . . . . . . . . . . . . . . . . . . . . . . . . . . . . . . . . . . . . . . . . . . . . . . . . . . . . . . . . . . . C C C C C C C C C C    
1354	. . . . . . . . . . . . . . . . . . . . . . . . . . . . . . . . . . . . . . . . . . . . . . . . . . . . . . . . . . . . . . . . . . . . C C C C C C C C C C    
1355	. . . . . . . . . . . . . . . . . . . . . . . . . . . . . . . . . . . . . . . . . . . . . . . . . . . . . . . . . . . . . . . . . . . . G G G G G G G G G G    
1356	. . . . . . . . . . . . . . . . . . . . . . . . . . . . . . . . . . . . . . . . . . . . . . . . . . . . . . . . . . . . . . . . . . . . A A A A A A A A A A    
1357	. . . . . . . . . . . . . . . . . . . . . . . . . . . . . . . . . . . . . . . . . . . . . . . . . . . . . . . . . . . . . . . . . . . . C C C C C C C C C C    
1358	. . . . . . . . . . . . . . . . . . . . . . . . . . . . . . . . . . . . . . . . . . . . . . . . . . . . . . . . . . . . . . . . . . . . A A A A A A A A A A    
1359	. . . . . . . . . . . . . . . . . . . . . . . . . . . . . . . . . . . . . . . . . . . . . . . . . . . . . . . . . . . . . . . . . . . . A A A A A A A A A A    
1360	. . . . . . . . . . . . . . . . . . . . . . . . . . . . . . . . . . . . . . . . . . . . . . . . . . . . . . . . . . . . . . . . . . . . A A A A A A A A A A    
1361	. . . . . . . . . . . . . . . . . . . . . . . . . . . . . . . . . . . . . . . . . . . . . . . . . . . . . . . . . . . . . . . . . . . . G G G G G G G G G G    
1362	. . . . . . . . . . . . . . . . . . . . . . . . . . . . . . . . . . . . . . . . . . . . . . . . . . . . . . . . . . . . . . . . . . . . G G G G G G G G G G    
1363	. . . . . . . . . . . . . . . . . . . . . . . . . . . . . . . . . . . . . . . . . . . . . . . . . . . . . . . . . . . . . . . . . . . . T T T T T T T T T T    
1364	. . . . . . . . . . . . . . . . . . . . . . . . . . . . . . . . . . . . . . . . . . . . . . . . . . . . . . . . . . . . . . . . . . . . G G G G G G G G G G    
1365	. . . . . . . . . . . . . . . . . . . . . . . . . . . . . . . . . . . . . . . . . . . . . . . . . . . . . . . . . . . . . . . . . . . . C C C C C C C C C C    
1366	. . . . . . . . . . . . . . . . . . . . . . . . . . . . . . . . . . . . . . . . . . . . . . . . . . . . . . . . . . . . . . . . . . . . T T T T T T T T T T    
1367	. . . . . . . . . . . . . . . . . . . . . . . . . . . . . . . . . . . . . . . . . . . . . . . . . . . . . . . . . . . . . . . . . . . . G G G G G G G G G G    
1368	. . . . . . . . . . . . . . . . . . . . . . . . . . . . . . . . . . . . . . . . . . . . . . . . . . . . . . . . . . . . . . . . . . . . G G G G G G G G G G    
1369	. . . . . . . . . . . . . . . . . . . . . . . . . . . . . . . . . . . . . . . . . . . . . . . . . . . . . . . . . . . . . . . . . . . . T T T T T T T T T T    
1370	. . . . . . . . . . . . . . . . . . . . . . . . . . . . . . . . . . . . . . . . . . . . . . . . . . . . . . . . . . . . . . . . . . . . T T T T T T T T T T    
1371	. . . . . . . . . . . . . . . . . . . . . . . . . . . . . . . . . . . . . . . . . . . . . . . . . . . . . . . . . . . . . . . . . . . . C C C C C C C C C C    
1372	. . . . . . . . . . . . . . . . . . . . . . . . . . . . . . . . . . . . . . . . . . . . . . . . . . . . . . . . . . . . . . . . . . . . C C C C C C C C C C    
1373	. . . . . . . . . . . . . . . . . . . . . . . . . . . . . . . . . . . . . . . . . . . . . . . . . . . . . . . . . . . . . . . . . . . . C C C C C C C C C C    
1374	. . . . . . . . . . . . . . . . . . . . . . . . . . . . . . . . . . . . . . . . . . . . . . . . . . . . . . . . . . . . . . . . . . . . T T T T T T T T T T    
1375	. . . . . . . . . . . . . . . . . . . . . . . . . . . . . . . . . . . . . . . . . . . . . . . . . . . . . . . . . . . . . . . . . . . . G G G G G G G G G G    
1376	. . . . . . . . . . . . . . . . . . . . . . . . . . . . . . . . . . . . . . . . . . . . . . . . . . . . . . . . . . . . . . . . . . . . G G G G G G G G G G    
1377	. . . . . . . . . . . . . . . . . . . . . . . . . . . . . . . . . . . . . . . . . . . . . . . . . . . . . . . . . . . . . . . . . . . . T G G G G G G G G G    
1378	. . . . . . . . . . . . . . . . . . . . . . . . . . . . . . . . . . . . . . . . . . . . . . . . . . . . . . . . . . . . . . . . . . . . . T T T T T T T T T    
1379	. . . . . . . . . . . . . . . . . . . . . . . . . . . . . . . . . . . . . . . . . . . . . . . . . . . . . . . . . . . . . . . . . . . . . C C C C C C C C C    
1380	. . . . . . . . . . . . . . . . . . . . . . . . . . . . . . . . . . . . . . . . . . . . . . . . . . . . . . . . . . . . . . . . . . . . . C C C C C C C C C    
1381	. . . . . . . . . . . . . . . . . . . . . . . . . . . . . . . . . . . . . . . . . . . . . . . . . . . . . . . . . . . . . . . . . . . . . T T T T T T T T T    
1382	. . . . . . . . . . . . . . . . . . . . . . . . . . . . . . . . . . . . . . . . . . . . . . . . . . . . . . . . . . . . . . . . . . . . . T T T T T T T T T    
1383	. . . . . . . . . . . . . . . . . . . . . . . . . . . . . . . . . . . . . . . . . . . . . . . . . . . . . . . . . . . . . . . . . . . . . C C C C C C C C C    
1384	. . . . . . . . . . . . . . . . . . . . . . . . . . . . . . . . . . . . . . . . . . . . . . . . . . . . . . . . . . . . . . . . . . . . . T T T T T T T T T    
1385	. . . . . . . . . . . . . . . . . . . . . . . . . . . . . . . . . . . . . . . . . . . . . . . . . . . . . . . . . . . . . . . . . . . . . G G G G G G G G G    
1386	. . . . . . . . . . . . . . . . . . . . . . . . . . . . . . . . . . . . . . . . . . . . . . . . . . . . . . . . . . . . . . . . . . . . . G G G G G G G G G    
1387	. . . . . . . . . . . . . . . . . . . . . . . . . . . . . . . . . . . . . . . . . . . . . . . . . . . . . . . . . . . . . . . . . . . . . C C C C C C C C C    
1388	. . . . . . . . . . . . . . . . . . . . . . . . . . . . . . . . . . . . . . . . . . . . . . . . . . . . . . . . . . . . . . . . . . . . . C C C C C C C C C    
1389	. . . . . . . . . . . . . . . . . . . . . . . . . . . . . . . . . . . . . . . . . . . . . . . . . . . . . . . . . . . . . . . . . . . . . A A A A A A A A A    
1390	. . . . . . . . . . . . . . . . . . . . . . . . . . . . . . . . . . . . . . . . . . . . . . . . . . . . . . . . . . . . . . . . . . . . . G G G G G G G G G    
1391	. . . . . . . . . . . . . . . . . . . . . . . . . . . . . . . . . . . . . . . . . . . . . . . . . . . . . . . . . . . . . . . . . . . . . C C C C C C C C C    
1392	. . . . . . . . . . . . . . . . . . . . . . . . . . . . . . . . . . . . . . . . . . . . . . . . . . . . . . . . . . . . . . . . . . . . . . T T T T T T T T    
1393	. . . . . . . . . . . . . . . . . . . . . . . . . . . . . . . . . . . . . . . . . . . . . . . . . . . . . . . . . . . . . . . . . . . . . . C C C C C C C C    
1394	. . . . . . . . . . . . . . . . . . . . . . . . . . . . . . . . . . . . . . . . . . . . . . . . . . . . . . . . . . . . . . . . . . . . . . T T T T T T T T    
1395	. . . . . . . . . . . . . . . . . . . . . . . . . . . . . . . . . . . . . . . . . . . . . . . . . . . . . . . . . . . . . . . . . . . . . . G G G G G G G G    
1396	. . . . . . . . . . . . . . . . . . . . . . . . . . . . . . . . . . . . . . . . . . . . . . . . . . . . . . . . . . . . . . . . . . . . . . G G G G G G G G    
1397	. . . . . . . . . . . . . . . . . . . . . . . . . . . . . . . . . . . . . . . . . . . . . . . . . . . . . . . . . . . . . . . . . . . . . . T T T T T T T T    
1398	. . . . . . . . . . . . . . . . . . . . . . . . . . . . . . . . . . . . . . . . . . . . . . . . . . . . . . . . . . . . . . . . . . . . . . G G G G G G G G    
1399	. . . . . . . . . . . . . . . . . . . . . . . . . . . . . . . . . . . . . . . . . . . . . . . . . . . . . . . . . . . . . . . . . . . . . . G G G G G G G G    
1400	. . . . . . . . . . . . . . . . . . . . . . . . . . . . . . . . . . . . . . . . . . . . . . . . . . . . . . . . . . . . . . . . . . . . . . C C C C C C C C    
1401	. . . . . . . . . . . . . . . . . . . . . . . . . . . . . . . . . . . . . . . . . . . . . . . . . . . . . . . . . . . . . . . . . . . . . . C C C C C C C C    
1402	. . . . . . . . . . . . . . . . . . . . . . . . . . . . . . . . . . . . . . . . . . . . . . . . . . . . . . . . . . . . . . . . . . . . . . C C C C C C C C    
1403	. . . . . . . . . . . . . . . . . . . . . . . . . . . . . . . . . . . . . . . . . . . . . . . . . . . . . . . . . . . . . . . . . . . . . . G G G G G G G G    
1404	. . . . . . . . . . . . . . . . . . . . . . . . . . . . . . . . . . . . . . . . . . . . . . . . . . . . . . . . . . . . . . . . . . . . . . C C C C C C C C    
1405	. . . . . . . . . . . . . . . . . . . . . . . . . . . . . . . . . . . . . . . . . . . . . . . . . . . . . . . . . . . . . . . . . . . . . . T T T T T T T T    
1406	. . . . . . . . . . . . . . . . . . . . . . . . . . . . . . . . . . . . . . . . . . . . . . . . . . . . . . . . . . . . . . . . . . . . . . G G G G G G G G    
1407	. . . . . . . . . . . . . . . . . . . . . . . . . . . . . . . . . . . . . . . . . . . . . . . . . . . . . . . . . . . . . . . . . . . . . . C C C C C C C C    
1408	. . . . . . . . . . . . . . . . . . . . . . . . . . . . . . . . . . . . . . . . . . . . . . . . . . . . . . . . . . . . . . . . . . . . . . C C C C C C C C    
1409	. . . . . . . . . . . . . . . . . . . . . . . . . . . . . . . . . . . . . . . . . . . . . . . . . . . . . . . . . . . . . . . . . . . . . . C C C C C C C C    
1410	. . . . . . . . . . . . . . . . . . . . . . . . . . . . . . . . . . . . . . . . . . . . . . . . . . . . . . . . . . . . . . . . . . . . . . C C C C C C C C    
1411	. . . . . . . . . . . . . . . . . . . . . . . . . . . . . . . . . . . . . . . . . . . . . . . . . . . . . . . . . . . . . . . . . . . . . . C C C C C C C C    
1412	. . . . . . . . . . . . . . . . . . . . . . . . . . . . . . . . . . . . . . . . . . . . . . . . . . . . . . . . . . . . . . . . . . . . . . T T T T T T T T    
1413	. . . . . . . . . . . . . . . . . . . . . . . . . . . . . . . . . . . . . . . . . . . . . . . . . . . . . . . . . . . . . . . . . . . . . . C C C C C C C C    
1414	. . . . . . . . . . . . . . . . . . . . . . . . . . . . . . . . . . . . . . . . . . . . . . . . . . . . . . . . . . . . . . . . . . . . . . T T T T T T T T    
1415	. . . . . . . . . . . . . . . . . . . . . . . . . . . . . . . . . . . . . . . . . . . . . . . . . . . . . . . . . . . . . . . . . . . . . . C C C C C C C C    
1416	. . . . . . . . . . . . . . . . . . . . . . . . . . . . . . . . . . . . . . . . . . . . . . . . . . . . . . . . . . . . . . . . . . . . . . C C C C C C C C    
1417	. . . . . . . . . . . . . . . . . . . . . . . . . . . . . . . . . . . . . . . . . . . . . . . . . . . . . . . . . . . . . . . . . . . . . . A A A A A A A A    
1418	. . . . . . . . . . . . . . . . . . . . . . . . . . . . . . . . . . . . . . . . . . . . . . . . . . . . . . . . . . . . . . . . . . . . . . C C C C C C C C    
1419	. . . . . . . . . . . . . . . . . . . . . . . . . . . . . . . . . . . . . . . . . . . . . . . . . . . . . . . . . . . . . . . . . . . . . . A A A A A A A A    
1420	. . . . . . . . . . . . . . . . . . . . . . . . . . . . . . . . . . . . . . . . . . . . . . . . . . . . . . . . . . . . . . . . . . . . . . T T T T T T T T    
1421	. . . . . . . . . . . . . . . . . . . . . . . . . . . . . . . . . . . . . . . . . . . . . . . . . . . . . . . . . . . . . . . . . . . . . . C C C C C C C C    
1422	. . . . . . . . . . . . . . . . . . . . . . . . . . . . . . . . . . . . . . . . . . . . . . . . . . . . . . . . . . . . . . . . . . . . . . C C C C C C C C    
1423	. . . . . . . . . . . . . . . . . . . . . . . . . . . . . . . . . . . . . . . . . . . . . . . . . . . . . . . . . . . . . . . . . . . . . . C C C C C C C C    
1424	. . . . . . . . . . . . . . . . . . . . . . . . . . . . . . . . . . . . . . . . . . . . . . . . . . . . . . . . . . . . . . . . . . . . . . A A A A A A A A    
1425	. . . . . . . . . . . . . . . . . . . . . . . . . . . . . . . . . . . . . . . . . . . . . . . . . . . . . . . . . . . . . . . . . . . . . . G G G G G G G G    
1426	. . . . . . . . . . . . . . . . . . . . . . . . . . . . . . . . . . . . . . . . . . . . . . . . . . . . . . . . . . . . . . . . . . . . . . G G G G G G G G    
1427	. . . . . . . . . . . . . . . . . . . . . . . . . . . . . . . . . . . . . . . . . . . . . . . . . . . . . . . . . . . . . . . . . . . . . . C C C C C C C C    
1428	. . . . . . . . . . . . . . . . . . . . . . . . . . . . . . . . . . . . . . . . . . . . . . . . . . . . . . . . . . . . . . . . . . . . . . T T T T T T T T    
1429	. . . . . . . . . . . . . . . . . . . . . . . . . . . . . . . . . . . . . . . . . . . . . . . . . . . . . . . . . . . . . . . . . . . . . . C C C C C C C C    
1430	. . . . . . . . . . . . . . . . . . . . . . . . . . . . . . . . . . . . . . . . . . . . . . . . . . . . . . . . . . . . . . . . . . . . . . C C C C C C C C    
1431	. . . . . . . . . . . . . . . . . . . . . . . . . . . . . . . . . . . . . . . . . . . . . . . . . . . . . . . . . . . . . . . . . . . . . . C C C C C C C C    
1432	. . . . . . . . . . . . . . . . . . . . . . . . . . . . . . . . . . . . . . . . . . . . . . . . . . . . . . . . . . . . . . . . . . . . . . A A A A A A A A    
1433	. . . . . . . . . . . . . . . . . . . . . . . . . . . . . . . . . . . . . . . . . . . . . . . . . . . . . . . . . . . . . . . . . . . . . . C C C C C C C C    
1434	. . . . . . . . . . . . . . . . . . . . . . . . . . . . . . . . . . . . . . . . . . . . . . . . . . . . . . . . . . . . . . . . . . . . . . T T T T T T T T    
1435	. . . . . . . . . . . . . . . . . . . . . . . . . . . . . . . . . . . . . . . . . . . . . . . . . . . . . . . . . . . . . . . . . . . . . . A A A A A A A A    
1436	. . . . . . . . . . . . . . . . . . . . . . . . . . . . . . . . . . . . . . . . . . . . . . . . . . . . . . . . . . . . . . . . . . . . . . C C C C C C C C    
1437	. . . . . . . . . . . . . . . . . . . . . . . . . . . . . . . . . . . . . . . . . . . . . . . . . . . . . . . . . . . . . . . . . . . . . . T T T T T T T T    
1438	. . . . . . . . . . . . . . . . . . . . . . . . . . . . . . . . . . . . . . . . . . . . . . . . . . . . . . . . . . . . . . . . . . . . . . G G G G G G G G    
1439	. . . . . . . . . . . . . . . . . . . . . . . . . . . . . . . . . . . . . . . . . . . . . . . . . . . . . . . . . . . . . . . . . . . . . . T T T T T T T T    
1440	. . . . . . . . . . . . . . . . . . . . . . . . . . . . . . . . . . . . . . . . . . . . . . . . . . . . . . . . . . . . . . . . . . . . . . C C C C C C C C    
1441	. . . . . . . . . . . . . . . . . . . . . . . . . . . . . . . . . . . . . . . . . . . . . . . . . . . . . . . . . . . . . . . . . . . . . . T T T T T T T T    
1442	. . . . . . . . . . . . . . . . . . . . . . . . . . . . . . . . . . . . . . . . . . . . . . . . . . . . . . . . . . . . . . . . . . . . . . G G G G G G G G    
1443	. . . . . . . . . . . . . . . . . . . . . . . . . . . . . . . . . . . . . . . . . . . . . . . . . . . . . . . . . . . . . . . . . . . . . . C C C C C C C C    
1444	. . . . . . . . . . . . . . . . . . . . . . . . . . . . . . . . . . . . . . . . . . . . . . . . . . . . . . . . . . . . . . . . . . . . . . A A A A A A A A    
1445	. . . . . . . . . . . . . . . . . . . . . . . . . . . . . . . . . . . . . . . . . . . . . . . . . . . . . . . . . . . . . . . . . . . . . . A G G G G G G G    
1446	. . . . . . . . . . . . . . . . . . . . . . . . . . . . . . . . . . . . . . . . . . . . . . . . . . . . . . . . . . . . . . . . . . . . . . . G G G G G G G    
1447	. . . . . . . . . . . . . . . . . . . . . . . . . . . . . . . . . . . . . . . . . . . . . . . . . . . . . . . . . . . . . . . . . . . . . . . G G G G G G G    
1448	. . . . . . . . . . . . . . . . . . . . . . . . . . . . . . . . . . . . . . . . . . . . . . . . . . . . . . . . . . . . . . . . . . . . . . . C C C C C C C    
1449	. . . . . . . . . . . . . . . . . . . . . . . . . . . . . . . . . . . . . . . . . . . . . . . . . . . . . . . . . . . . . . . . . . . . . . . C C C C C C C    
1450	. . . . . . . . . . . . . . . . . . . . . . . . . . . . . . . . . . . . . . . . . . . . . . . . . . . . . . . . . . . . . . . . . . . . . . . T T T T T T T    
1451	. . . . . . . . . . . . . . . . . . . . . . . . . . . . . . . . . . . . . . . . . . . . . . . . . . . . . . . . . . . . . . . . . . . . . . . T T T T T T T    
1452	. . . . . . . . . . . . . . . . . . . . . . . . . . . . . . . . . . . . . . . . . . . . . . . . . . . . . . . . . . . . . . . . . . . . . . . C C C C C C C    
1453	. . . . . . . . . . . . . . . . . . . . . . . . . . . . . . . . . . . . . . . . . . . . . . . . . . . . . . . . . . . . . . . . . . . . . . . A A A A A A A    
1454	. . . . . . . . . . . . . . . . . . . . . . . . . . . . . . . . . . . . . . . . . . . . . . . . . . . . . . . . . . . . . . . . . . . . . . . G G G G G G G    
1455	. . . . . . . . . . . . . . . . . . . . . . . . . . . . . . . . . . . . . . . . . . . . . . . . . . . . . . . . . . . . . . . . . . . . . . . C C C C C C C    
1456	. . . . . . . . . . . . . . . . . . . . . . . . . . . . . . . . . . . . . . . . . . . . . . . . . . . . . . . . . . . . . . . . . . . . . . . A A A A A A A    
1457	. . . . . . . . . . . . . . . . . . . . . . . . . . . . . . . . . . . . . . . . . . . . . . . . . . . . . . . . . . . . . . . . . . . . . . . G G G G G G G    
1458	. . . . . . . . . . . . . . . . . . . . . . . . . . . . . . . . . . . . . . . . . . . . . . . . . . . . . . . . . . . . . . . . . . . . . . . A A A A A A A    
1459	. . . . . . . . . . . . . . . . . . . . . . . . . . . . . . . . . . . . . . . . . . . . . . . . . . . . . . . . . . . . . . . . . . . . . . . A A A A A A A    
1460	. . . . . . . . . . . . . . . . . . . . . . . . . . . . . . . . . . . . . . . . . . . . . . . . . . . . . . . . . . . . . . . . . . . . . . . G G G G G G G    
1461	. . . . . . . . . . . . . . . . . . . . . . . . . . . . . . . . . . . . . . . . . . . . . . . . . . . . . . . . . . . . . . . . . . . . . . . C C C C C C C    
1462	. . . . . . . . . . . . . . . . . . . . . . . . . . . . . . . . . . . . . . . . . . . . . . . . . . . . . . . . . . . . . . . . . . . . . . . A A A A A A A    
1463	. . . . . . . . . . . . . . . . . . . . . . . . . . . . . . . . . . . . . . . . . . . . . . . . . . . . . . . . . . . . . . . . . . . . . . . G G G G G G G    
1464	. . . . . . . . . . . . . . . . . . . . . . . . . . . . . . . . . . . . . . . . . . . . . . . . . . . . . . . . . . . . . . . . . . . . . . . G G G G G G G    
1465	. . . . . . . . . . . . . . . . . . . . . . . . . . . . . . . . . . . . . . . . . . . . . . . . . . . . . . . . . . . . . . . . . . . . . . . G G G G G G G    
1466	. . . . . . . . . . . . . . . . . . . . . . . . . . . . . . . . . . . . . . . . . . . . . . . . . . . . . . . . . . . . . . . . . . . . . . . C C C C C C C    
1467	. . . . . . . . . . . . . . . . . . . . . . . . . . . . . . . . . . . . . . . . . . . . . . . . . . . . . . . . . . . . . . . . . . . . . . . T T T T T T T    
1468	. . . . . . . . . . . . . . . . . . . . . . . . . . . . . . . . . . . . . . . . . . . . . . . . . . . . . . . . . . . . . . . . . . . . . . . C C C C C C C    
1469	. . . . . . . . . . . . . . . . . . . . . . . . . . . . . . . . . . . . . . . . . . . . . . . . . . . . . . . . . . . . . . . . . . . . . . . G G G G G G G    
1470	. . . . . . . . . . . . . . . . . . . . . . . . . . . . . . . . . . . . . . . . . . . . . . . . . . . . . . . . . . . . . . . . . . . . . . . G G G G G G G    
1471	. . . . . . . . . . . . . . . . . . . . . . . . . . . . . . . . . . . . . . . . . . . . . . . . . . . . . . . . . . . . . . . . . . . . . . . T T T T T T T    
1472	. . . . . . . . . . . . . . . . . . . . . . . . . . . . . . . . . . . . . . . . . . . . . . . . . . . . . . . . . . . . . . . . . . . . . . . A A A A A A A    
1473	. . . . . . . . . . . . . . . . . . . . . . . . . . . . . . . . . . . . . . . . . . . . . . . . . . . . . . . . . . . . . . . . . . . . . . . C C C C C C C    
1474	. . . . . . . . . . . . . . . . . . . . . . . . . . . . . . . . . . . . . . . . . . . . . . . . . . . . . . . . . . . . . . . . . . . . . . . T T T T T T T    
1475	. . . . . . . . . . . . . . . . . . . . . . . . . . . . . . . . . . . . . . . . . . . . . . . . . . . . . . . . . . . . . . . . . . . . . . . G G G G G G G    
1476	. . . . . . . . . . . . . . . . . . . . . . . . . . . . . . . . . . . . . . . . . . . . . . . . . . . . . . . . . . . . . . . . . . . . . . . C C C C C C C    
1477	. . . . . . . . . . . . . . . . . . . . . . . . . . . . . . . . . . . . . . . . . . . . . . . . . . . . . . . . . . . . . . . . . . . . . . . G G G G G G G    
1478	. . . . . . . . . . . . . . . . . . . . . . . . . . . . . . . . . . . . . . . . . . . . . . . . . . . . . . . . . . . . . . . . . . . . . . . T T T T T T T    
1479	. . . . . . . . . . . . . . . . . . . . . . . . . . . . . . . . . . . . . . . . . . . . . . . . . . . . . . . . . . . . . . . . . . . . . . . C C C C C C C    
1480	. . . . . . . . . . . . . . . . . . . . . . . . . . . . . . . . . . . . . . . . . . . . . . . . . . . . . . . . . . . . . . . . . . . . . . . T T T T T T T    
1481	. . . . . . . . . . . . . . . . . . . . . . . . . . . . . . . . . . . . . . . . . . . . . . . . . . . . . . . . . . . . . . . . . . . . . . . C C C C C C C    
1482	. . . . . . . . . . . . . . . . . . . . . . . . . . . . . . . . . . . . . . . . . . . . . . . . . . . . . . . . . . . . . . . . . . . . . . . A A A A A A A    
1483	. . . . . . . . . . . . . . . . . . . . . . . . . . . . . . . . . . . . . . . . . . . . . . . . . . . . . . . . . . . . . . . . . . . . . . . C C C C C C C    
1484	. . . . . . . . . . . . . . . . . . . . . . . . . . . . . . . . . . . . . . . . . . . . . . . . . . . . . . . . . . . . . . . . . . . . . . . T T T T T T T    
1485	. . . . . . . . . . . . . . . . . . . . . . . . . . . . . . . . . . . . . . . . . . . . . . . . . . . . . . . . . . . . . . . . . . . . . . . G G G G G G G    
1486	. . . . . . . . . . . . . . . . . . . . . . . . . . . . . . . . . . . . . . . . . . . . . . . . . . . . . . . . . . . . . . . . . . . . . . . G G G G G G G    
1487	. . . . . . . . . . . . . . . . . . . . . . . . . . . . . . . . . . . . . . . . . . . . . . . . . . . . . . . . . . . . . . . . . . . . . . . G G G G G G G    
1488	. . . . . . . . . . . . . . . . . . . . . . . . . . . . . . . . . . . . . . . . . . . . . . . . . . . . . . . . . . . . . . . . . . . . . . . G G G G G G G    
1489	. . . . . . . . . . . . . . . . . . . . . . . . . . . . . . . . . . . . . . . . . . . . . . . . . . . . . . . . . . . . . . . . . . . . . . . C C C C C C C    
1490	. . . . . . . . . . . . . . . . . . . . . . . . . . . . . . . . . . . . . . . . . . . . . . . . . . . . . . . . . . . . . . . . . . . . . . . T T T T T T T    
1491	. . . . . . . . . . . . . . . . . . . . . . . . . . . . . . . . . . . . . . . . . . . . . . . . . . . . . . . . . . . . . . . . . . . . . . . C C C C C C C    
1492	. . . . . . . . . . . . . . . . . . . . . . . . . . . . . . . . . . . . . . . . . . . . . . . . . . . . . . . . . . . . . . . . . . . . . . . C C C C C C C    
1493	. . . . . . . . . . . . . . . . . . . . . . . . . . . . . . . . . . . . . . . . . . . . . . . . . . . . . . . . . . . . . . . . . . . . . . . C C C C C C C    
1494	. . . . . . . . . . . . . . . . . . . . . . . . . . . . . . . . . . . . . . . . . . . . . . . . . . . . . . . . . . . . . . . . . . . . . . . A A A A A A A    
1495	. . . . . . . . . . . . . . . . . . . . . . . . . . . . . . . . . . . . . . . . . . . . . . . . . . . . . . . . . . . . . . . . . . . . . . . A A A A A A A    
1496	. . . . . . . . . . . . . . . . . . . . . . . . . . . . . . . . . . . . . . . . . . . . . . . . . . . . . . . . . . . . . . . . . . . . . . . C C C C C C C    
1497	. . . . . . . . . . . . . . . . . . . . . . . . . . . . . . . . . . . . . . . . . . . . . . . . . . . . . . . . . . . . . . . . . . . . . . . A A A A A A A    
1498	. . . . . . . . . . . . . . . . . . . . . . . . . . . . . . . . . . . . . . . . . . . . . . . . . . . . . . . . . . . . . . . . . . . . . . . C C C C C C C    
1499	. . . . . . . . . . . . . . . . . . . . . . . . . . . . . . . . . . . . . . . . . . . . . . . . . . . . . . . . . . . . . . . . . . . . . . . C C C C C C C    
1500	. . . . . . . . . . . . . . . . . . . . . . . . . . . . . . . . . . . . . . . . . . . . . . . . . . . . . . . . . . . . . . . . . . . . . . . T T T T T T T    
1501	. . . . . . . . . . . . . . . . . . . . . . . . . . . . . . . . . . . . . . . . . . . . . . . . . . . . . . . . . . . . . . . . . . . . . . . G G G G G G G    
1502	. . . . . . . . . . . . . . . . . . . . . . . . . . . . . . . . . . . . . . . . . . . . . . . . . . . . . . . . . . . . . . . . . . . . . . . C C C C C C C    
1503	. . . . . . . . . . . . . . . . . . . . . . . . . . . . . . . . . . . . . . . . . . . . . . . . . . . . . . . . . . . . . . . . . . . . . . . T T T T T T T    
1504	. . . . . . . . . . . . . . . . . . . . . . . . . . . . . . . . . . . . . . . . . . . . . . . . . . . . . . . . . . . . . . . . . . . . . . . G G G G G G G    
1505	. . . . . . . . . . . . . . . . . . . . . . . . . . . . . . . . . . . . . . . . . . . . . . . . . . . . . . . . . . . . . . . . . . . . . . . G G G G G G G    
1506	. . . . . . . . . . . . . . . . . . . . . . . . . . . . . . . . . . . . . . . . . . . . . . . . . . . . . . . . . . . . . . . . . . . . . . . T T T T T T T    
1507	. . . . . . . . . . . . . . . . . . . . . . . . . . . . . . . . . . . . . . . . . . . . . . . . . . . . . . . . . . . . . . . . . . . . . . . G G G G G G G    
1508	. . . . . . . . . . . . . . . . . . . . . . . . . . . . . . . . . . . . . . . . . . . . . . . . . . . . . . . . . . . . . . . . . . . . . . . C C C C C C C    
1509	. . . . . . . . . . . . . . . . . . . . . . . . . . . . . . . . . . . . . . . . . . . . . . . . . . . . . . . . . . . . . . . . . . . . . . . T T T T T T T    
1510	. . . . . . . . . . . . . . . . . . . . . . . . . . . . . . . . . . . . . . . . . . . . . . . . . . . . . . . . . . . . . . . . . . . . . . . T T T T T T T    
1511	. . . . . . . . . . . . . . . . . . . . . . . . . . . . . . . . . . . . . . . . . . . . . . . . . . . . . . . . . . . . . . . . . . . . . . . C C C C C C C    
1512	. . . . . . . . . . . . . . . . . . . . . . . . . . . . . . . . . . . . . . . . . . . . . . . . . . . . . . . . . . . . . . . . . . . . . . . C C C C C C C    
1513	. . . . . . . . . . . . . . . . . . . . . . . . . . . . . . . . . . . . . . . . . . . . . . . . . . . . . . . . . . . . . . . . . . . . . . . T T T T T T T    
1514	. . . . . . . . . . . . . . . . . . . . . . . . . . . . . . . . . . . . . . . . . . . . . . . . . . . . . . . . . . . . . . . . . . . . . . . C C C C C C C    
1515	. . . . . . . . . . . . . . . . . . . . . . . . . . . . . . . . . . . . . . . . . . . . . . . . . . . . . . . . . . . . . . . . . . . . . . . G G G G G G G    
1516	. . . . . . . . . . . . . . . . . . . . . . . . . . . . . . . . . . . . . . . . . . . . . . . . . . . . . . . . . . . . . . . . . . . . . . . C C C C C C C    
1517	. . . . . . . . . . . . . . . . . . . . . . . . . . . . . . . . . . . . . . . . . . . . . . . . . . . . . . . . . . . . . . . . . . . . . . . C C C C C C C    
1518	. . . . . . . . . . . . . . . . . . . . . . . . . . . . . . . . . . . . . . . . . . . . . . . . . . . . . . . . . . . . . . . . . . . . . . . C C C C C C C    
1519	. . . . . . . . . . . . . . . . . . . . . . . . . . . . . . . . . . . . . . . . . . . . . . . . . . . . . . . . . . . . . . . . . . . . . . . T T T T T T T    
1520	. . . . . . . . . . . . . . . . . . . . . . . . . . . . . . . . . . . . . . . . . . . . . . . . . . . . . . . . . . . . . . . . . . . . . . . C C C C C C C    
1521	. . . . . . . . . . . . . . . . . . . . . . . . . . . . . . . . . . . . . . . . . . . . . . . . . . . . . . . . . . . . . . . . . . . . . . . T T T T T T T    
1522	. . . . . . . . . . . . . . . . . . . . . . . . . . . . . . . . . . . . . . . . . . . . . . . . . . . . . . . . . . . . . . . . . . . . . . . T T T T T T T    
1523	. . . . . . . . . . . . . . . . . . . . . . . . . . . . . . . . . . . . . . . . . . . . . . . . . . . . . . . . . . . . . . . . . . . . . . . T T T T T T T    
1524	. . . . . . . . . . . . . . . . . . . . . . . . . . . . . . . . . . . . . . . . . . . . . . . . . . . . . . . . . . . . . . . . . . . . . . . A A A A A A A    
1525	. . . . . . . . . . . . . . . . . . . . . . . . . . . . . . . . . . . . . . . . . . . . . . . . . . . . . . . . . . . . . . . . . . . . . . . G G G G G G G    
1526	. . . . . . . . . . . . . . . . . . . . . . . . . . . . . . . . . . . . . . . . . . . . . . . . . . . . . . . . . . . . . . . . . . . . . . . G G G G G G G    
1527	. . . . . . . . . . . . . . . . . . . . . . . . . . . . . . . . . . . . . . . . . . . . . . . . . . . . . . . . . . . . . . . . . . . . . . . A A A A A A A    
1528	. . . . . . . . . . . . . . . . . . . . . . . . . . . . . . . . . . . . . . . . . . . . . . . . . . . . . . . . . . . . . . . . . . . . . . . A A A A A A A    
1529	. . . . . . . . . . . . . . . . . . . . . . . . . . . . . . . . . . . . . . . . . . . . . . . . . . . . . . . . . . . . . . . . . . . . . . . G G G G G G G    
1530	. . . . . . . . . . . . . . . . . . . . . . . . . . . . . . . . . . . . . . . . . . . . . . . . . . . . . . . . . . . . . . . . . . . . . . . G G G G G G G    
1531	. . . . . . . . . . . . . . . . . . . . . . . . . . . . . . . . . . . . . . . . . . . . . . . . . . . . . . . . . . . . . . . . . . . . . . . A A A A A A A    
1532	. . . . . . . . . . . . . . . . . . . . . . . . . . . . . . . . . . . . . . . . . . . . . . . . . . . . . . . . . . . . . . . . . . . . . . . C C C C C C C    
1533	. . . . . . . . . . . . . . . . . . . . . . . . . . . . . . . . . . . . . . . . . . . . . . . . . . . . . . . . . . . . . . . . . . . . . . . A A A A A A A    
1534	. . . . . . . . . . . . . . . . . . . . . . . . . . . . . . . . . . . . . . . . . . . . . . . . . . . . . . . . . . . . . . . . . . . . . . . C C C C C C C    
1535	. . . . . . . . . . . . . . . . . . . . . . . . . . . . . . . . . . . . . . . . . . . . . . . . . . . . . . . . . . . . . . . . . . . . . . . T T T T T T T    
1536	. . . . . . . . . . . . . . . . . . . . . . . . . . . . . . . . . . . . . . . . . . . . . . . . . . . . . . . . . . . . . . . . . . . . . . . G G G G G G G    
1537	. . . . . . . . . . . . . . . . . . . . . . . . . . . . . . . . . . . . . . . . . . . . . . . . . . . . . . . . . . . . . . . . . . . . . . . A A A A A A A    
1538	. . . . . . . . . . . . . . . . . . . . . . . . . . . . . . . . . . . . . . . . . . . . . . . . . . . . . . . . . . . . . . . . . . . . . . . G G G G G G G    
1539	. . . . . . . . . . . . . . . . . . . . . . . . . . . . . . . . . . . . . . . . . . . . . . . . . . . . . . . . . . . . . . . . . . . . . . . T T T T T T T    
1540	. . . . . . . . . . . . . . . . . . . . . . . . . . . . . . . . . . . . . . . . . . . . . . . . . . . . . . . . . . . . . . . . . . . . . . . T T T T T T T    
1541	. . . . . . . . . . . . . . . . . . . . . . . . . . . . . . . . . . . . . . . . . . . . . . . . . . . . . . . . . . . . . . . . . . . . . . . C C C C C C C    
1542	. . . . . . . . . . . . . . . . . . . . . . . . . . . . . . . . . . . . . . . . . . . . . . . . . . . . . . . . . . . . . . . . . . . . . . . C C C C C C C    
1543	. . . . . . . . . . . . . . . . . . . . . . . . . . . . . . . . . . . . . . . . . . . . . . . . . . . . . . . . . . . . . . . . . . . . . . . T T T T T T T    
1544	. . . . . . . . . . . . . . . . . . . . . . . . . . . . . . . . . . . . . . . . . . . . . . . . . . . . . . . . . . . . . . . . . . . . . . . G G G G G G G    
1545	. . . . . . . . . . . . . . . . . . . . . . . . . . . . . . . . . . . . . . . . . . . . . . . . . . . . . . . . . . . . . . . . . . . . . . . G G G G G G G    
1546	. . . . . . . . . . . . . . . . . . . . . . . . . . . . . . . . . . . . . . . . . . . . . . . . . . . . . . . . . . . . . . . . . . . . . . . A A A A A A A    
1547	. . . . . . . . . . . . . . . . . . . . . . . . . . . . . . . . . . . . . . . . . . . . . . . . . . . . . . . . . . . . . . . . . . . . . . . G G G G G G G    
1548	. . . . . . . . . . . . . . . . . . . . . . . . . . . . . . . . . . . . . . . . . . . . . . . . . . . . . . . . . . . . . . . . . . . . . . . T T T T T T T    
1549	. . . . . . . . . . . . . . . . . . . . . . . . . . . . . . . . . . . . . . . . . . . . . . . . . . . . . . . . . . . . . . . . . . . . . . . C C C C C C C    
1550	. . . . . . . . . . . . . . . . . . . . . . . . . . . . . . . . . . . . . . . . . . . . . . . . . . . . . . . . . . . . . . . . . . . . . . . T T T T T T T    
1551	. . . . . . . . . . . . . . . . . . . . . . . . . . . . . . . . . . . . . . . . . . . . . . . . . . . . . . . . . . . . . . . . . . . . . . . . C C C C C C    
1552	. . . . . . . . . . . . . . . . . . . . . . . . . . . . . . . . . . . . . . . . . . . . . . . . . . . . . . . . . . . . . . . . . . . . . . . . C C C C C C    
1553	. . . . . . . . . . . . . . . . . . . . . . . . . . . . . . . . . . . . . . . . . . . . . . . . . . . . . . . . . . . . . . . . . . . . . . . . T T T T T T    
1554	. . . . . . . . . . . . . . . . . . . . . . . . . . . . . . . . . . . . . . . . . . . . . . . . . . . . . . . . . . . . . . . . . . . . . . . . C C C C C C    
1555	. . . . . . . . . . . . . . . . . . . . . . . . . . . . . . . . . . . . . . . . . . . . . . . . . . . . . . . . . . . . . . . . . . . . . . . . A A A A A A    
1556	. . . . . . . . . . . . . . . . . . . . . . . . . . . . . . . . . . . . . . . . . . . . . . . . . . . . . . . . . . . . . . . . . . . . . . . . G G G G G G    
1557	. . . . . . . . . . . . . . . . . . . . . . . . . . . . . . . . . . . . . . . . . . . . . . . . . . . . . . . . . . . . . . . . . . . . . . . . T T T T T T    
1558	. . . . . . . . . . . . . . . . . . . . . . . . . . . . . . . . . . . . . . . . . . . . . . . . . . . . . . . . . . . . . . . . . . . . . . . . C C C C C C    
1559	. . . . . . . . . . . . . . . . . . . . . . . . . . . . . . . . . . . . . . . . . . . . . . . . . . . . . . . . . . . . . . . . . . . . . . . . T T T T T T    
1560	. . . . . . . . . . . . . . . . . . . . . . . . . . . . . . . . . . . . . . . . . . . . . . . . . . . . . . . . . . . . . . . . . . . . . . . . G G G G G G    
1561	. . . . . . . . . . . . . . . . . . . . . . . . . . . . . . . . . . . . . . . . . . . . . . . . . . . . . . . . . . . . . . . . . . . . . . . . T T T T T T    
1562	. . . . . . . . . . . . . . . . . . . . . . . . . . . . . . . . . . . . . . . . . . . . . . . . . . . . . . . . . . . . . . . . . . . . . . . . C C C C C C    
1563	. . . . . . . . . . . . . . . . . . . . . . . . . . . . . . . . . . . . . . . . . . . . . . . . . . . . . . . . . . . . . . . . . . . . . . . . C . C C C C    
1564	. . . . . . . . . . . . . . . . . . . . . . . . . . . . . . . . . . . . . . . . . . . . . . . . . . . . . . . . . . . . . . . . . . . . . . . . A . A A A A    
1565	. . . . . . . . . . . . . . . . . . . . . . . . . . . . . . . . . . . . . . . . . . . . . . . . . . . . . . . . . . . . . . . . . . . . . . . . T . T T T T    
1566	. . . . . . . . . . . . . . . . . . . . . . . . . . . . . . . . . . . . . . . . . . . . . . . . . . . . . . . . . . . . . . . . . . . . . . . . C . C G C C    
1567	. . . . . . . . . . . . . . . . . . . . . . . . . . . . . . . . . . . . . . . . . . . . . . . . . . . . . . . . . . . . . . . . . . . . . . . . C . C C C C    
1568	. . . . . . . . . . . . . . . . . . . . . . . . . . . . . . . . . . . . . . . . . . . . . . . . . . . . . . . . . . . . . . . . . . . . . . . . C . C C C C    
1569	. . . . . . . . . . . . . . . . . . . . . . . . . . . . . . . . . . . . . . . . . . . . . . . . . . . . . . . . . . . . . . . . . . . . . . . . T . T T T T    
1570	. . . . . . . . . . . . . . . . . . . . . . . . . . . . . . . . . . . . . . . . . . . . . . . . . . . . . . . . . . . . . . . . . . . . . . . . G . G G G G    
1571	. . . . . . . . . . . . . . . . . . . . . . . . . . . . . . . . . . . . . . . . . . . . . . . . . . . . . . . . . . . . . . . . . . . . . . . . C . C C C C    
1572	. . . . . . . . . . . . . . . . . . . . . . . . . . . . . . . . . . . . . . . . . . . . . . . . . . . . . . . . . . . . . . . . . . . . . . . . T . T T T T    
1573	. . . . . . . . . . . . . . . . . . . . . . . . . . . . . . . . . . . . . . . . . . . . . . . . . . . . . . . . . . . . . . . . . . . . . . . . T . T T T T    
1574	. . . . . . . . . . . . . . . . . . . . . . . . . . . . . . . . . . . . . . . . . . . . . . . . . . . . . . . . . . . . . . . . . . . . . . . . T . T T T T    
1575	. . . . . . . . . . . . . . . . . . . . . . . . . . . . . . . . . . . . . . . . . . . . . . . . . . . . . . . . . . . . . . . . . . . . . . . . G . G G G G    
1576	. . . . . . . . . . . . . . . . . . . . . . . . . . . . . . . . . . . . . . . . . . . . . . . . . . . . . . . . . . . . . . . . . . . . . . . . C . C C C C    
1577	. . . . . . . . . . . . . . . . . . . . . . . . . . . . . . . . . . . . . . . . . . . . . . . . . . . . . . . . . . . . . . . . . . . . . . . . C . C C C C    
1578	. . . . . . . . . . . . . . . . . . . . . . . . . . . . . . . . . . . . . . . . . . . . . . . . . . . . . . . . . . . . . . . . . . . . . . . . C . C C C C    
1579	. . . . . . . . . . . . . . . . . . . . . . . . . . . . . . . . . . . . . . . . . . . . . . . . . . . . . . . . . . . . . . . . . . . . . . . . A . A A A A    
1580	. . . . . . . . . . . . . . . . . . . . . . . . . . . . . . . . . . . . . . . . . . . . . . . . . . . . . . . . . . . . . . . . . . . . . . . . C . C C C C    
1581	. . . . . . . . . . . . . . . . . . . . . . . . . . . . . . . . . . . . . . . . . . . . . . . . . . . . . . . . . . . . . . . . . . . . . . . . C . C C C C    
1582	. . . . . . . . . . . . . . . . . . . . . . . . . . . . . . . . . . . . . . . . . . . . . . . . . . . . . . . . . . . . . . . . . . . . . . . . . . T T T T    
1583	. . . . . . . . . . . . . . . . . . . . . . . . . . . . . . . . . . . . . . . . . . . . . . . . . . . . . . . . . . . . . . . . . . . . . . . . . . C C C C    
1584	. . . . . . . . . . . . . . . . . . . . . . . . . . . . . . . . . . . . . . . . . . . . . . . . . . . . . . . . . . . . . . . . . . . . . . . . . . C C C C    
1585	. . . . . . . . . . . . . . . . . . . . . . . . . . . . . . . . . . . . . . . . . . . . . . . . . . . . . . . . . . . . . . . . . . . . . . . . . . T T T T    
1586	. . . . . . . . . . . . . . . . . . . . . . . . . . . . . . . . . . . . . . . . . . . . . . . . . . . . . . . . . . . . . . . . . . . . . . . . . . C C C C    
1587	. . . . . . . . . . . . . . . . . . . . . . . . . . . . . . . . . . . . . . . . . . . . . . . . . . . . . . . . . . . . . . . . . . . . . . . . . . C C C C    
1588	. . . . . . . . . . . . . . . . . . . . . . . . . . . . . . . . . . . . . . . . . . . . . . . . . . . . . . . . . . . . . . . . . . . . . . . . . . G G G G    
1589	. . . . . . . . . . . . . . . . . . . . . . . . . . . . . . . . . . . . . . . . . . . . . . . . . . . . . . . . . . . . . . . . . . . . . . . . . . C C C C    
1590	. . . . . . . . . . . . . . . . . . . . . . . . . . . . . . . . . . . . . . . . . . . . . . . . . . . . . . . . . . . . . . . . . . . . . . . . . . C C C C    
1591	. . . . . . . . . . . . . . . . . . . . . . . . . . . . . . . . . . . . . . . . . . . . . . . . . . . . . . . . . . . . . . . . . . . . . . . . . . C C C C    
1592	. . . . . . . . . . . . . . . . . . . . . . . . . . . . . . . . . . . . . . . . . . . . . . . . . . . . . . . . . . . . . . . . . . . . . . . . . . T T T T    
1593	. . . . . . . . . . . . . . . . . . . . . . . . . . . . . . . . . . . . . . . . . . . . . . . . . . . . . . . . . . . . . . . . . . . . . . . . . . C C C C    
1594	. . . . . . . . . . . . . . . . . . . . . . . . . . . . . . . . . . . . . . . . . . . . . . . . . . . . . . . . . . . . . . . . . . . . . . . . . . A A A A    
1595	. . . . . . . . . . . . . . . . . . . . . . . . . . . . . . . . . . . . . . . . . . . . . . . . . . . . . . . . . . . . . . . . . . . . . . . . . . T T T T    
1596	. . . . . . . . . . . . . . . . . . . . . . . . . . . . . . . . . . . . . . . . . . . . . . . . . . . . . . . . . . . . . . . . . . . . . . . . . . C C C C    
1597	. . . . . . . . . . . . . . . . . . . . . . . . . . . . . . . . . . . . . . . . . . . . . . . . . . . . . . . . . . . . . . . . . . . . . . . . . . T T T T    
1598	. . . . . . . . . . . . . . . . . . . . . . . . . . . . . . . . . . . . . . . . . . . . . . . . . . . . . . . . . . . . . . . . . . . . . . . . . . G G G G    
1599	. . . . . . . . . . . . . . . . . . . . . . . . . . . . . . . . . . . . . . . . . . . . . . . . . . . . . . . . . . . . . . . . . . . . . . . . . . A A A A    
1600	. . . . . . . . . . . . . . . . . . . . . . . . . . . . . . . . . . . . . . . . . . . . . . . . . . . . . . . . . . . . . . . . . . . . . . . . . . A A A A    
1601	. . . . . . . . . . . . . . . . . . . . . . . . . . . . . . . . . . . . . . . . . . . . . . . . . . . . . . . . . . . . . . . . . . . . . . . . . . C C C C    
1602	. . . . . . . . . . . . . . . . . . . . . . . . . . . . . . . . . . . . . . . . . . . . . . . . . . . . . . . . . . . . . . . . . . . . . . . . . . C C C C    
1603	. . . . . . . . . . . . . . . . . . . . . . . . . . . . . . . . . . . . . . . . . . . . . . . . . . . . . . . . . . . . . . . . . . . . . . . . . . A A A A    
1604	. . . . . . . . . . . . . . . . . . . . . . . . . . . . . . . . . . . . . . . . . . . . . . . . . . . . . . . . . . . . . . . . . . . . . . . . . . G G G G    
1605	. . . . . . . . . . . . . . . . . . . . . . . . . . . . . . . . . . . . . . . . . . . . . . . . . . . . . . . . . . . . . . . . . . . . . . . . . . A A A A    
1606	. . . . . . . . . . . . . . . . . . . . . . . . . . . . . . . . . . . . . . . . . . . . . . . . . . . . . . . . . . . . . . . . . . . . . . . . . . A A A A    
1607	. . . . . . . . . . . . . . . . . . . . . . . . . . . . . . . . . . . . . . . . . . . . . . . . . . . . . . . . . . . . . . . . . . . . . . . . . . C C C C    
1608	. . . . . . . . . . . . . . . . . . . . . . . . . . . . . . . . . . . . . . . . . . . . . . . . . . . . . . . . . . . . . . . . . . . . . . . . . . C C C C    
1609	. . . . . . . . . . . . . . . . . . . . . . . . . . . . . . . . . . . . . . . . . . . . . . . . . . . . . . . . . . . . . . . . . . . . . . . . . . T T T T    
1610	. . . . . . . . . . . . . . . . . . . . . . . . . . . . . . . . . . . . . . . . . . . . . . . . . . . . . . . . . . . . . . . . . . . . . . . . . . C C C C    
1611	. . . . . . . . . . . . . . . . . . . . . . . . . . . . . . . . . . . . . . . . . . . . . . . . . . . . . . . . . . . . . . . . . . . . . . . . . . C C C C    
1612	. . . . . . . . . . . . . . . . . . . . . . . . . . . . . . . . . . . . . . . . . . . . . . . . . . . . . . . . . . . . . . . . . . . . . . . . . . T T T T    
1613	. . . . . . . . . . . . . . . . . . . . . . . . . . . . . . . . . . . . . . . . . . . . . . . . . . . . . . . . . . . . . . . . . . . . . . . . . . C C C C    
1614	. . . . . . . . . . . . . . . . . . . . . . . . . . . . . . . . . . . . . . . . . . . . . . . . . . . . . . . . . . . . . . . . . . . . . . . . . . C C C C    
1615	. . . . . . . . . . . . . . . . . . . . . . . . . . . . . . . . . . . . . . . . . . . . . . . . . . . . . . . . . . . . . . . . . . . . . . . . . . A A A A    
1616	. . . . . . . . . . . . . . . . . . . . . . . . . . . . . . . . . . . . . . . . . . . . . . . . . . . . . . . . . . . . . . . . . . . . . . . . . . G G G G    
1617	. . . . . . . . . . . . . . . . . . . . . . . . . . . . . . . . . . . . . . . . . . . . . . . . . . . . . . . . . . . . . . . . . . . . . . . . . . C C C C    
1618	. . . . . . . . . . . . . . . . . . . . . . . . . . . . . . . . . . . . . . . . . . . . . . . . . . . . . . . . . . . . . . . . . . . . . . . . . . C C C C    
1619	. . . . . . . . . . . . . . . . . . . . . . . . . . . . . . . . . . . . . . . . . . . . . . . . . . . . . . . . . . . . . . . . . . . . . . . . . . T T T T    
1620	. . . . . . . . . . . . . . . . . . . . . . . . . . . . . . . . . . . . . . . . . . . . . . . . . . . . . . . . . . . . . . . . . . . . . . . . . . C C C C    
1621	. . . . . . . . . . . . . . . . . . . . . . . . . . . . . . . . . . . . . . . . . . . . . . . . . . . . . . . . . . . . . . . . . . . . . . . . . . T T T T    
1622	. . . . . . . . . . . . . . . . . . . . . . . . . . . . . . . . . . . . . . . . . . . . . . . . . . . . . . . . . . . . . . . . . . . . . . . . . . G G G G    
1623	. . . . . . . . . . . . . . . . . . . . . . . . . . . . . . . . . . . . . . . . . . . . . . . . . . . . . . . . . . . . . . . . . . . . . . . . . . C C C C    
1624	. . . . . . . . . . . . . . . . . . . . . . . . . . . . . . . . . . . . . . . . . . . . . . . . . . . . . . . . . . . . . . . . . . . . . . . . . . C C C C    
1625	. . . . . . . . . . . . . . . . . . . . . . . . . . . . . . . . . . . . . . . . . . . . . . . . . . . . . . . . . . . . . . . . . . . . . . . . . . T T T T    
1626	. . . . . . . . . . . . . . . . . . . . . . . . . . . . . . . . . . . . . . . . . . . . . . . . . . . . . . . . . . . . . . . . . . . . . . . . . . G G G G    
1627	. . . . . . . . . . . . . . . . . . . . . . . . . . . . . . . . . . . . . . . . . . . . . . . . . . . . . . . . . . . . . . . . . . . . . . . . . . A A A A    
1628	. . . . . . . . . . . . . . . . . . . . . . . . . . . . . . . . . . . . . . . . . . . . . . . . . . . . . . . . . . . . . . . . . . . . . . . . . . G . G G    
1629	. . . . . . . . . . . . . . . . . . . . . . . . . . . . . . . . . . . . . . . . . . . . . . . . . . . . . . . . . . . . . . . . . . . . . . . . . . C . C C    
1630	. . . . . . . . . . . . . . . . . . . . . . . . . . . . . . . . . . . . . . . . . . . . . . . . . . . . . . . . . . . . . . . . . . . . . . . . . . A . A A    
1631	. . . . . . . . . . . . . . . . . . . . . . . . . . . . . . . . . . . . . . . . . . . . . . . . . . . . . . . . . . . . . . . . . . . . . . . . . . C . C C    
1632	. . . . . . . . . . . . . . . . . . . . . . . . . . . . . . . . . . . . . . . . . . . . . . . . . . . . . . . . . . . . . . . . . . . . . . . . . . C . C C    
1633	. . . . . . . . . . . . . . . . . . . . . . . . . . . . . . . . . . . . . . . . . . . . . . . . . . . . . . . . . . . . . . . . . . . . . . . . . . T . T T    
1634	. . . . . . . . . . . . . . . . . . . . . . . . . . . . . . . . . . . . . . . . . . . . . . . . . . . . . . . . . . . . . . . . . . . . . . . . . . . . C C    
1635	. . . . . . . . . . . . . . . . . . . . . . . . . . . . . . . . . . . . . . . . . . . . . . . . . . . . . . . . . . . . . . . . . . . . . . . . . . . . C C    
1636	. . . . . . . . . . . . . . . . . . . . . . . . . . . . . . . . . . . . . . . . . . . . . . . . . . . . . . . . . . . . . . . . . . . . . . . . . . . . A A    
1637	. . . . . . . . . . . . . . . . . . . . . . . . . . . . . . . . . . . . . . . . . . . . . . . . . . . . . . . . . . . . . . . . . . . . . . . . . . . . G G    
1638	. . . . . . . . . . . . . . . . . . . . . . . . . . . . . . . . . . . . . . . . . . . . . . . . . . . . . . . . . . . . . . . . . . . . . . . . . . . . T T    
1639	. . . . . . . . . . . . . . . . . . . . . . . . . . . . . . . . . . . . . . . . . . . . . . . . . . . . . . . . . . . . . . . . . . . . . . . . . . . . A A    
1640	. . . . . . . . . . . . . . . . . . . . . . . . . . . . . . . . . . . . . . . . . . . . . . . . . . . . . . . . . . . . . . . . . . . . . . . . . . . . G G    
1641	. . . . . . . . . . . . . . . . . . . . . . . . . . . . . . . . . . . . . . . . . . . . . . . . . . . . . . . . . . . . . . . . . . . . . . . . . . . . G G    
1642	. . . . . . . . . . . . . . . . . . . . . . . . . . . . . . . . . . . . . . . . . . . . . . . . . . . . . . . . . . . . . . . . . . . . . . . . . . . . G G    
1643	. . . . . . . . . . . . . . . . . . . . . . . . . . . . . . . . . . . . . . . . . . . . . . . . . . . . . . . . . . . . . . . . . . . . . . . . . . . . C C    
1644	. . . . . . . . . . . . . . . . . . . . . . . . . . . . . . . . . . . . . . . . . . . . . . . . . . . . . . . . . . . . . . . . . . . . . . . . . . . . G G    
1645	. . . . . . . . . . . . . . . . . . . . . . . . . . . . . . . . . . . . . . . . . . . . . . . . . . . . . . . . . . . . . . . . . . . . . . . . . . . . G G    
1646	. . . . . . . . . . . . . . . . . . . . . . . . . . . . . . . . . . . . . . . . . . . . . . . . . . . . . . . . . . . . . . . . . . . . . . . . . . . . G G    
1647	. . . . . . . . . . . . . . . . . . . . . . . . . . . . . . . . . . . . . . . . . . . . . . . . . . . . . . . . . . . . . . . . . . . . . . . . . . . . C C    
1648	. . . . . . . . . . . . . . . . . . . . . . . . . . . . . . . . . . . . . . . . . . . . . . . . . . . . . . . . . . . . . . . . . . . . . . . . . . . . T T    
1649	. . . . . . . . . . . . . . . . . . . . . . . . . . . . . . . . . . . . . . . . . . . . . . . . . . . . . . . . . . . . . . . . . . . . . . . . . . . . G G    
1650	. . . . . . . . . . . . . . . . . . . . . . . . . . . . . . . . . . . . . . . . . . . . . . . . . . . . . . . . . . . . . . . . . . . . . . . . . . . . C C    
1651	. . . . . . . . . . . . . . . . . . . . . . . . . . . . . . . . . . . . . . . . . . . . . . . . . . . . . . . . . . . . . . . . . . . . . . . . . . . . A A    
1652	. . . . . . . . . . . . . . . . . . . . . . . . . . . . . . . . . . . . . . . . . . . . . . . . . . . . . . . . . . . . . . . . . . . . . . . . . . . . G G    
1653	. . . . . . . . . . . . . . . . . . . . . . . . . . . . . . . . . . . . . . . . . . . . . . . . . . . . . . . . . . . . . . . . . . . . . . . . . . . . G G    
1654	. . . . . . . . . . . . . . . . . . . . . . . . . . . . . . . . . . . . . . . . . . . . . . . . . . . . . . . . . . . . . . . . . . . . . . . . . . . . G G    
1655	. . . . . . . . . . . . . . . . . . . . . . . . . . . . . . . . . . . . . . . . . . . . . . . . . . . . . . . . . . . . . . . . . . . . . . . . . . . . A A    
1656	. . . . . . . . . . . . . . . . . . . . . . . . . . . . . . . . . . . . . . . . . . . . . . . . . . . . . . . . . . . . . . . . . . . . . . . . . . . . C C    
1657	. . . . . . . . . . . . . . . . . . . . . . . . . . . . . . . . . . . . . . . . . . . . . . . . . . . . . . . . . . . . . . . . . . . . . . . . . . . . A A    
1658	. . . . . . . . . . . . . . . . . . . . . . . . . . . . . . . . . . . . . . . . . . . . . . . . . . . . . . . . . . . . . . . . . . . . . . . . . . . . G G    
1659	. . . . . . . . . . . . . . . . . . . . . . . . . . . . . . . . . . . . . . . . . . . . . . . . . . . . . . . . . . . . . . . . . . . . . . . . . . . . C C    
1660	. . . . . . . . . . . . . . . . . . . . . . . . . . . . . . . . . . . . . . . . . . . . . . . . . . . . . . . . . . . . . . . . . . . . . . . . . . . . T T    
1661	. . . . . . . . . . . . . . . . . . . . . . . . . . . . . . . . . . . . . . . . . . . . . . . . . . . . . . . . . . . . . . . . . . . . . . . . . . . . C C    
1662	. . . . . . . . . . . . . . . . . . . . . . . . . . . . . . . . . . . . . . . . . . . . . . . . . . . . . . . . . . . . . . . . . . . . . . . . . . . . C C    
1663	. . . . . . . . . . . . . . . . . . . . . . . . . . . . . . . . . . . . . . . . . . . . . . . . . . . . . . . . . . . . . . . . . . . . . . . . . . . . C C    
1664	. . . . . . . . . . . . . . . . . . . . . . . . . . . . . . . . . . . . . . . . . . . . . . . . . . . . . . . . . . . . . . . . . . . . . . . . . . . . C C    
1665	. . . . . . . . . . . . . . . . . . . . . . . . . . . . . . . . . . . . . . . . . . . . . . . . . . . . . . . . . . . . . . . . . . . . . . . . . . . . G G    
1666	. . . . . . . . . . . . . . . . . . . . . . . . . . . . . . . . . . . . . . . . . . . . . . . . . . . . . . . . . . . . . . . . . . . . . . . . . . . . T T    
1667	. . . . . . . . . . . . . . . . . . . . . . . . . . . . . . . . . . . . . . . . . . . . . . . . . . . . . . . . . . . . . . . . . . . . . . . . . . . . G G    
1668	. . . . . . . . . . . . . . . . . . . . . . . . . . . . . . . . . . . . . . . . . . . . . . . . . . . . . . . . . . . . . . . . . . . . . . . . . . . . A A    
1669	. . . . . . . . . . . . . . . . . . . . . . . . . . . . . . . . . . . . . . . . . . . . . . . . . . . . . . . . . . . . . . . . . . . . . . . . . . . . T T    
1670	. . . . . . . . . . . . . . . . . . . . . . . . . . . . . . . . . . . . . . . . . . . . . . . . . . . . . . . . . . . . . . . . . . . . . . . . . . . . G G    
1671	. . . . . . . . . . . . . . . . . . . . . . . . . . . . . . . . . . . . . . . . . . . . . . . . . . . . . . . . . . . . . . . . . . . . . . . . . . . . C C    
1672	. . . . . . . . . . . . . . . . . . . . . . . . . . . . . . . . . . . . . . . . . . . . . . . . . . . . . . . . . . . . . . . . . . . . . . . . . . . . C C    
1673	. . . . . . . . . . . . . . . . . . . . . . . . . . . . . . . . . . . . . . . . . . . . . . . . . . . . . . . . . . . . . . . . . . . . . . . . . . . . C C    
1674	. . . . . . . . . . . . . . . . . . . . . . . . . . . . . . . . . . . . . . . . . . . . . . . . . . . . . . . . . . . . . . . . . . . . . . . . . . . . C C    
1675	. . . . . . . . . . . . . . . . . . . . . . . . . . . . . . . . . . . . . . . . . . . . . . . . . . . . . . . . . . . . . . . . . . . . . . . . . . . . T T    
1676	. . . . . . . . . . . . . . . . . . . . . . . . . . . . . . . . . . . . . . . . . . . . . . . . . . . . . . . . . . . . . . . . . . . . . . . . . . . . G G    
1677	. . . . . . . . . . . . . . . . . . . . . . . . . . . . . . . . . . . . . . . . . . . . . . . . . . . . . . . . . . . . . . . . . . . . . . . . . . . . G G    
1678	. . . . . . . . . . . . . . . . . . . . . . . . . . . . . . . . . . . . . . . . . . . . . . . . . . . . . . . . . . . . . . . . . . . . . . . . . . . . A A    
1679	. . . . . . . . . . . . . . . . . . . . . . . . . . . . . . . . . . . . . . . . . . . . . . . . . . . . . . . . . . . . . . . . . . . . . . . . . . . . G G    
1680	. . . . . . . . . . . . . . . . . . . . . . . . . . . . . . . . . . . . . . . . . . . . . . . . . . . . . . . . . . . . . . . . . . . . . . . . . . . . C C    
1681	. . . . . . . . . . . . . . . . . . . . . . . . . . . . . . . . . . . . . . . . . . . . . . . . . . . . . . . . . . . . . . . . . . . . . . . . . . . . A A    
1682	. . . . . . . . . . . . . . . . . . . . . . . . . . . . . . . . . . . . . . . . . . . . . . . . . . . . . . . . . . . . . . . . . . . . . . . . . . . . C C    
1683	. . . . . . . . . . . . . . . . . . . . . . . . . . . . . . . . . . . . . . . . . . . . . . . . . . . . . . . . . . . . . . . . . . . . . . . . . . . . A A    
1684	. . . . . . . . . . . . . . . . . . . . . . . . . . . . . . . . . . . . . . . . . . . . . . . . . . . . . . . . . . . . . . . . . . . . . . . . . . . . G G    
1685	. . . . . . . . . . . . . . . . . . . . . . . . . . . . . . . . . . . . . . . . . . . . . . . . . . . . . . . . . . . . . . . . . . . . . . . . . . . . A A    
1686	. . . . . . . . . . . . . . . . . . . . . . . . . . . . . . . . . . . . . . . . . . . . . . . . . . . . . . . . . . . . . . . . . . . . . . . . . . . . G G    
1687	. . . . . . . . . . . . . . . . . . . . . . . . . . . . . . . . . . . . . . . . . . . . . . . . . . . . . . . . . . . . . . . . . . . . . . . . . . . . C C    
1688	. . . . . . . . . . . . . . . . . . . . . . . . . . . . . . . . . . . . . . . . . . . . . . . . . . . . . . . . . . . . . . . . . . . . . . . . . . . . C C    
1689	. . . . . . . . . . . . . . . . . . . . . . . . . . . . . . . . . . . . . . . . . . . . . . . . . . . . . . . . . . . . . . . . . . . . . . . . . . . . C C    
1690	. . . . . . . . . . . . . . . . . . . . . . . . . . . . . . . . . . . . . . . . . . . . . . . . . . . . . . . . . . . . . . . . . . . . . . . . . . . . T T    
1691	. . . . . . . . . . . . . . . . . . . . . . . . . . . . . . . . . . . . . . . . . . . . . . . . . . . . . . . . . . . . . . . . . . . . . . . . . . . . G G    
1692	. . . . . . . . . . . . . . . . . . . . . . . . . . . . . . . . . . . . . . . . . . . . . . . . . . . . . . . . . . . . . . . . . . . . . . . . . . . . T T    
1693	. . . . . . . . . . . . . . . . . . . . . . . . . . . . . . . . . . . . . . . . . . . . . . . . . . . . . . . . . . . . . . . . . . . . . . . . . . . . G G    
1694	. . . . . . . . . . . . . . . . . . . . . . . . . . . . . . . . . . . . . . . . . . . . . . . . . . . . . . . . . . . . . . . . . . . . . . . . . . . . T T    
1695	. . . . . . . . . . . . . . . . . . . . . . . . . . . . . . . . . . . . . . . . . . . . . . . . . . . . . . . . . . . . . . . . . . . . . . . . . . . . G G    
1696	. . . . . . . . . . . . . . . . . . . . . . . . . . . . . . . . . . . . . . . . . . . . . . . . . . . . . . . . . . . . . . . . . . . . . . . . . . . . G G    
1697	. . . . . . . . . . . . . . . . . . . . . . . . . . . . . . . . . . . . . . . . . . . . . . . . . . . . . . . . . . . . . . . . . . . . . . . . . . . . G G    
1698	. . . . . . . . . . . . . . . . . . . . . . . . . . . . . . . . . . . . . . . . . . . . . . . . . . . . . . . . . . . . . . . . . . . . . . . . . . . . . G    
1699	. . . . . . . . . . . . . . . . . . . . . . . . . . . . . . . . . . . . . . . . . . . . . . . . . . . . . . . . . . . . . . . . . . . . . . . . . . . . . A    
1700	. . . . . . . . . . . . . . . . . . . . . . . . . . . . . . . . . . . . . . . . . . . . . . . . . . . . . . . . . . . . . . . . . . . . . . . . . . . . . A    
1701	. . . . . . . . . . . . . . . . . . . . . . . . . . . . . . . . . . . . . . . . . . . . . . . . . . . . . . . . . . . . . . . . . . . . . . . . . . . . . A    
1702	. . . . . . . . . . . . . . . . . . . . . . . . . . . . . . . . . . . . . . . . . . . . . . . . . . . . . . . . . . . . . . . . . . . . . . . . . . . . . G    
1703	. . . . . . . . . . . . . . . . . . . . . . . . . . . . . . . . . . . . . . . . . . . . . . . . . . . . . . . . . . . . . . . . . . . . . . . . . . . . . A    
1704	. . . . . . . . . . . . . . . . . . . . . . . . . . . . . . . . . . . . . . . . . . . . . . . . . . . . . . . . . . . . . . . . . . . . . . . . . . . . . G    
1705	. . . . . . . . . . . . . . . . . . . . . . . . . . . . . . . . . . . . . . . . . . . . . . . . . . . . . . . . . . . . . . . . . . . . . . . . . . . . . A    
1706	. . . . . . . . . . . . . . . . . . . . . . . . . . . . . . . . . . . . . . . . . . . . . . . . . . . . . . . . . . . . . . . . . . . . . . . . . . . . . A    
1707	. . . . . . . . . . . . . . . . . . . . . . . . . . . . . . . . . . . . . . . . . . . . . . . . . . . . . . . . . . . . . . . . . . . . . . . . . . . . . C    
1708	. . . . . . . . . . . . . . . . . . . . . . . . . . . . . . . . . . . . . . . . . . . . . . . . . . . . . . . . . . . . . . . . . . . . . . . . . . . . . A    
1709	. . . . . . . . . . . . . . . . . . . . . . . . . . . . . . . . . . . . . . . . . . . . . . . . . . . . . . . . . . . . . . . . . . . . . . . . . . . . . G    
1710	. . . . . . . . . . . . . . . . . . . . . . . . . . . . . . . . . . . . . . . . . . . . . . . . . . . . . . . . . . . . . . . . . . . . . . . . . . . . . G    
1711	. . . . . . . . . . . . . . . . . . . . . . . . . . . . . . . . . . . . . . . . . . . . . . . . . . . . . . . . . . . . . . . . . . . . . . . . . . . . . G    
1712	. . . . . . . . . . . . . . . . . . . . . . . . . . . . . . . . . . . . . . . . . . . . . . . . . . . . . . . . . . . . . . . . . . . . . . . . . . . . . T    
1713	. . . . . . . . . . . . . . . . . . . . . . . . . . . . . . . . . . . . . . . . . . . . . . . . . . . . . . . . . . . . . . . . . . . . . . . . . . . . . G    
1714	. . . . . . . . . . . . . . . . . . . . . . . . . . . . . . . . . . . . . . . . . . . . . . . . . . . . . . . . . . . . . . . . . . . . . . . . . . . . . G    
1715	. . . . . . . . . . . . . . . . . . . . . . . . . . . . . . . . . . . . . . . . . . . . . . . . . . . . . . . . . . . . . . . . . . . . . . . . . . . . . G    
1716	. . . . . . . . . . . . . . . . . . . . . . . . . . . . . . . . . . . . . . . . . . . . . . . . . . . . . . . . . . . . . . . . . . . . . . . . . . . . . A    
1717	. . . . . . . . . . . . . . . . . . . . . . . . . . . . . . . . . . . . . . . . . . . . . . . . . . . . . . . . . . . . . . . . . . . . . . . . . . . . . G    
1718	. . . . . . . . . . . . . . . . . . . . . . . . . . . . . . . . . . . . . . . . . . . . . . . . . . . . . . . . . . . . . . . . . . . . . . . . . . . . . C    
1719	. . . . . . . . . . . . . . . . . . . . . . . . . . . . . . . . . . . . . . . . . . . . . . . . . . . . . . . . . . . . . . . . . . . . . . . . . . . . . T    
1720	. . . . . . . . . . . . . . . . . . . . . . . . . . . . . . . . . . . . . . . . . . . . . . . . . . . . . . . . . . . . . . . . . . . . . . . . . . . . . G    
1721	. . . . . . . . . . . . . . . . . . . . . . . . . . . . . . . . . . . . . . . . . . . . . . . . . . . . . . . . . . . . . . . . . . . . . . . . . . . . . A    
1722	. . . . . . . . . . . . . . . . . . . . . . . . . . . . . . . . . . . . . . . . . . . . . . . . . . . . . . . . . . . . . . . . . . . . . . . . . . . . . G    
1723	. . . . . . . . . . . . . . . . . . . . . . . . . . . . . . . . . . . . . . . . . . . . . . . . . . . . . . . . . . . . . . . . . . . . . . . . . . . . . G    
1724	. . . . . . . . . . . . . . . . . . . . . . . . . . . . . . . . . . . . . . . . . . . . . . . . . . . . . . . . . . . . . . . . . . . . . . . . . . . . . A    
1725	. . . . . . . . . . . . . . . . . . . . . . . . . . . . . . . . . . . . . . . . . . . . . . . . . . . . . . . . . . . . . . . . . . . . . . . . . . . . . G    
1726	. . . . . . . . . . . . . . . . . . . . . . . . . . . . . . . . . . . . . . . . . . . . . . . . . . . . . . . . . . . . . . . . . . . . . . . . . . . . . T    
1727	. . . . . . . . . . . . . . . . . . . . . . . . . . . . . . . . . . . . . . . . . . . . . . . . . . . . . . . . . . . . . . . . . . . . . . . . . . . . . T    
1728	. . . . . . . . . . . . . . . . . . . . . . . . . . . . . . . . . . . . . . . . . . . . . . . . . . . . . . . . . . . . . . . . . . . . . . . . . . . . . G    
1729	. . . . . . . . . . . . . . . . . . . . . . . . . . . . . . . . . . . . . . . . . . . . . . . . . . . . . . . . . . . . . . . . . . . . . . . . . . . . . A    
1730	. . . . . . . . . . . . . . . . . . . . . . . . . . . . . . . . . . . . . . . . . . . . . . . . . . . . . . . . . . . . . . . . . . . . . . . . . . . . . G    
1731	. . . . . . . . . . . . . . . . . . . . . . . . . . . . . . . . . . . . . . . . . . . . . . . . . . . . . . . . . . . . . . . . . . . . . . . . . . . . . C    
1732	. . . . . . . . . . . . . . . . . . . . . . . . . . . . . . . . . . . . . . . . . . . . . . . . . . . . . . . . . . . . . . . . . . . . . . . . . . . . . A    
1733	. . . . . . . . . . . . . . . . . . . . . . . . . . . . . . . . . . . . . . . . . . . . . . . . . . . . . . . . . . . . . . . . . . . . . . . . . . . . . A    
1734	. . . . . . . . . . . . . . . . . . . . . . . . . . . . . . . . . . . . . . . . . . . . . . . . . . . . . . . . . . . . . . . . . . . . . . . . . . . . . C    
1735	. . . . . . . . . . . . . . . . . . . . . . . . . . . . . . . . . . . . . . . . . . . . . . . . . . . . . . . . . . . . . . . . . . . . . . . . . . . . . C    
1736	. . . . . . . . . . . . . . . . . . . . . . . . . . . . . . . . . . . . . . . . . . . . . . . . . . . . . . . . . . . . . . . . . . . . . . . . . . . . . T    
1737	. . . . . . . . . . . . . . . . . . . . . . . . . . . . . . . . . . . . . . . . . . . . . . . . . . . . . . . . . . . . . . . . . . . . . . . . . . . . . G    
1738	. . . . . . . . . . . . . . . . . . . . . . . . . . . . . . . . . . . . . . . . . . . . . . . . . . . . . . . . . . . . . . . . . . . . . . . . . . . . . G    
1739	. . . . . . . . . . . . . . . . . . . . . . . . . . . . . . . . . . . . . . . . . . . . . . . . . . . . . . . . . . . . . . . . . . . . . . . . . . . . . C    
1740	. . . . . . . . . . . . . . . . . . . . . . . . . . . . . . . . . . . . . . . . . . . . . . . . . . . . . . . . . . . . . . . . . . . . . . . . . . . . . A    
1741	. . . . . . . . . . . . . . . . . . . . . . . . . . . . . . . . . . . . . . . . . . . . . . . . . . . . . . . . . . . . . . . . . . . . . . . . . . . . . A    
1742	. . . . . . . . . . . . . . . . . . . . . . . . . . . . . . . . . . . . . . . . . . . . . . . . . . . . . . . . . . . . . . . . . . . . . . . . . . . . . G    
1743	. . . . . . . . . . . . . . . . . . . . . . . . . . . . . . . . . . . . . . . . . . . . . . . . . . . . . . . . . . . . . . . . . . . . . . . . . . . . . G    
1744	. . . . . . . . . . . . . . . . . . . . . . . . . . . . . . . . . . . . . . . . . . . . . . . . . . . . . . . . . . . . . . . . . . . . . . . . . . . . . A    
1745	. . . . . . . . . . . . . . . . . . . . . . . . . . . . . . . . . . . . . . . . . . . . . . . . . . . . . . . . . . . . . . . . . . . . . . . . . . . . . G    
1746	. . . . . . . . . . . . . . . . . . . . . . . . . . . . . . . . . . . . . . . . . . . . . . . . . . . . . . . . . . . . . . . . . . . . . . . . . . . . . C    
1747	. . . . . . . . . . . . . . . . . . . . . . . . . . . . . . . . . . . . . . . . . . . . . . . . . . . . . . . . . . . . . . . . . . . . . . . . . . . . . G    
1748	. . . . . . . . . . . . . . . . . . . . . . . . . . . . . . . . . . . . . . . . . . . . . . . . . . . . . . . . . . . . . . . . . . . . . . . . . . . . . T    
1749	. . . . . . . . . . . . . . . . . . . . . . . . . . . . . . . . . . . . . . . . . . . . . . . . . . . . . . . . . . . . . . . . . . . . . . . . . . . . . C    
1750	. . . . . . . . . . . . . . . . . . . . . . . . . . . . . . . . . . . . . . . . . . . . . . . . . . . . . . . . . . . . . . . . . . . . . . . . . . . . . T    
1751	. . . . . . . . . . . . . . . . . . . . . . . . . . . . . . . . . . . . . . . . . . . . . . . . . . . . . . . . . . . . . . . . . . . . . . . . . . . . . C    
1752	. . . . . . . . . . . . . . . . . . . . . . . . . . . . . . . . . . . . . . . . . . . . . . . . . . . . . . . . . . . . . . . . . . . . . . . . . . . . . A    
1753	. . . . . . . . . . . . . . . . . . . . . . . . . . . . . . . . . . . . . . . . . . . . . . . . . . . . . . . . . . . . . . . . . . . . . . . . . . . . . C
```
